# A nutrient-responsive AMPK/TBK1 circuit restricts adipocyte catabolism

**DOI:** 10.1172/jci.insight.200168

**Published:** 2026-05-08

**Authors:** Churaibhon Wisessaowapak, Yuliya Skorobogatko, Hyeonhui Kim, Xue Feng, Seunghwan Son, Haipeng Fu, Sitao Zhang, Pichaya Lertvilai, Lina Chang, Annie Hoang, Hetty Chen, Sarah Bedsted, Joseph Valentine, Jin Young Huh, Peng Zhao, Shannon M. Reilly, Piyajit Watcharasit, Maryam Ahmadian, Alan R. Saltiel

**Affiliations:** 1 Division of Endocrinology and Metabolism, Department of Medicine and Pharmacology;; 2 Department of Cellular and Molecular Medicine; and; 3 Marine Physical Lab, Scripps Institution of Oceanography, UCSD, San Diego, California, USA.; 4 Center for Nano Materials, Department of Life Science, Sogang University, Seoul, Korea.; 5 Department of Biochemistry and Structural Biology, University of Texas Health Science Center at San Antonio, San Antonio, Texas, USA.; 6 Weill Center for Metabolic Health, Division of Endocrinology, Diabetes and Metabolism, Department of Medicine, Weill Cornell Medicine, New York, New York, USA.; 7 Laboratory of Pharmacology, Chulabhorn Research Institute, Bangkok, Thailand.

**Keywords:** Endocrinology, Metabolism, Adipose tissue, Obesity

## Abstract

Metabolic adaptation to both caloric excess and restriction promotes energy conservation by suppressing catabolic pathways via feedback mechanisms that remain incompletely defined. We identified TANK binding kinase 1 (TBK1) as a nutrient- and inflammation-responsive brake on AMPK signaling in adipocytes. Fasting or pharmacological AMPK activation induced *Tbk1* transcription via a PGC1α/nuclear respiratory factor 1 axis, which, in turn, limited AMPK activity through a phosphorylation cascade to conserve energy. In obesity, this AMPK/TBK1 axis was disrupted due to chronically elevated basal TBK1, thereby restricting energy expenditure during fasting. Adipocyte-specific TBK1 deletion enhanced fasting-induced AMPK activation, mitochondrial function, and lipolytic gene expression in both lean and obese mice. Pharmacological TBK1 inhibition with amlexanox recapitulated these effects. Combined treatment of mice with amlexanox and the AMPK activator AICAR enhanced weight loss, improved glucose tolerance and insulin sensitivity, and suppressed inflammatory and lipogenic programs in adipose tissue, as well as fibrotic gene expression in the liver. Building on prior clinical observations linking TBK1 inhibition to metabolic health, these findings defined a nutrient-sensitive AMPK/TBK1 feedback loop that limited adipocyte catabolism and suggested that dual targeting of TBK1 and AMPK may help counteract metabolic adaptation and enhance the durability of obesity therapies.

## Abstract 2

TBK1 acts as a brake on fat burning during fasting. Combining TBK1 inhibitor with AMPK activator reverses obesity and improves metabolic health in mice.

## Graphical abstract

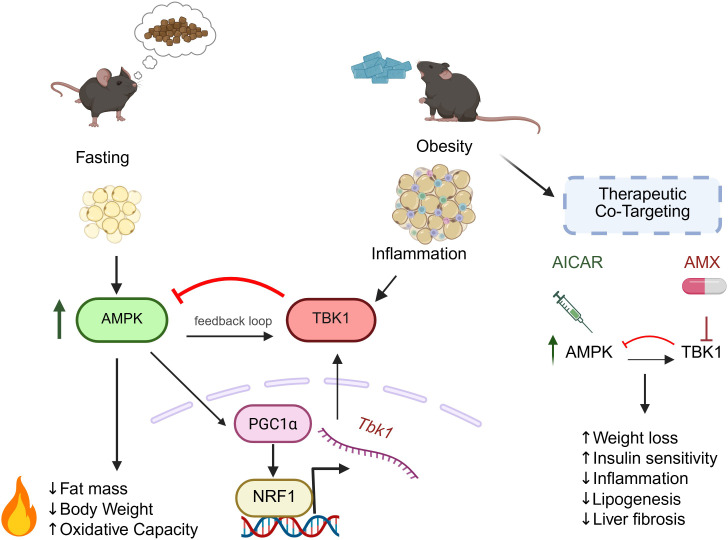

## Introduction

Obesity is a major driver of type 2 diabetes, metabolic dysfunction–associated steatotic liver disease (formerly NAFLD), cardiovascular disease, and premature mortality worldwide (1). Although caloric restriction alone through lifestyle modification or pharmacological therapies can produce weight loss, sustaining this effect is difficult due to metabolic adaptation resulting from the suppression of energy expenditure during both caloric excess and caloric restriction (2). This adaptive response reflects a strong evolutionary bias toward energy conservation and contributes to the difficulty of maintaining long-term weight loss (3). Adipose tissue is central to this adaptation, acting not only as a site of energy storage, but also as an active endocrine and metabolic organ that integrates nutrient, hormonal, and inflammatory cues (4). In obesity, chronic overnutrition drives a coordinated repression of energy-dissipating processes in adipose tissue, including reduced catecholamine sensitivity, diminished mitochondrial biogenesis, and lowered activity of AMPK (4, 5). Similar reductions in energy expenditure occur during prolonged caloric restriction, suggesting that both nutrient extremes engage shared suppressive mechanisms in adipocytes to defend energy stores.

AMPK is a heterotrimeric serine/threonine kinase that is activated by phosphorylation at Thr172 in response to an increased AMP/ATP ratio or calcium signaling via upstream kinases such as LKB1 (6). Once activated, AMPK promotes catabolic metabolism by inhibiting lipogenesis, stimulating fatty acid oxidation, and enhancing mitochondrial biogenesis through the transcriptional coactivator PGC1α (6, 7). In obesity, AMPK activity is markedly reduced, contributing to lipid accumulation, metabolic inflexibility, inflammation, and insulin resistance (8–10). Although diminished upstream activation, hyperinsulinemia, inflammation, and nutrient oversupply explain part of this defect, accumulating evidence suggests that active feedback mechanisms also constrain AMPK activity during sustained energy stress (4, 11).

TANK binding kinase 1 (TBK1) is a serine/threonine kinase best known for its role in innate immunity and inflammation (12). In metabolic tissues, TBK1 expression and activity are elevated in obesity (11, 13–17), where it reduces energy expenditure in part by suppressing AMPK signaling (11). Pharmacological or genetic inhibition of TBK1 in adipocytes increases lipid oxidation, mitochondrial oxidative capacity, and thermogenic gene expression, improving metabolic health in obese mice and, as reported in clinical trials, in humans (11, 13, 15). Notably, *Tbk1* mRNA is also induced during fasting, a state of robust AMPK activation (11, 14), suggesting that TBK1 may function as a nutrient-responsive brake that limits prolonged catabolic signaling under both nutrient excess and deprivation.

AMPK shapes transcriptional programs through coactivators such as PGC1α, which works with nuclear transcription factors like nuclear respiratory factor 1 (NRF1) to orchestrate mitochondrial oxidative metabolism (18). Although transcriptional programs downstream of AMPK activation are known to increase energy-burning gene networks (19), the regulation of *Tbk1* expression during fasting and how this control is altered in obesity remain unresolved. The functional role of fasting-induced TBK1 upregulation in adipocytes, particularly in relation to lipid mobilization and mitochondrial oxidative capacity, is also poorly understood, as is its potential as a therapeutic target alongside AMPK activation.

Here, we dissect the transcriptional control and functional role of TBK1 in adipocytes under nutrient stress. We show that fasting and pharmacological AMPK activation induce *Tbk1* via a PGC1α/NRF1 axis and that this induction is blunted in obesity. We hypothesize that once induced, TBK1 restrains AMPK signaling, mitochondrial oxidative capacity, and lipid mobilization during fasting. Finally, pharmacological TBK1 inhibition and AMPK activation act in concert to restore metabolic flexibility and improve whole-body metabolic health in obese mice, revealing a cotargeting strategy with potential to overcome the entrenched metabolic adaptation that undermines obesity treatment with fasting or anorexic drugs.

## Results

### Fasting induces Tbk1 expression in adipose tissue through AMPK activation.

**Figure 1:**
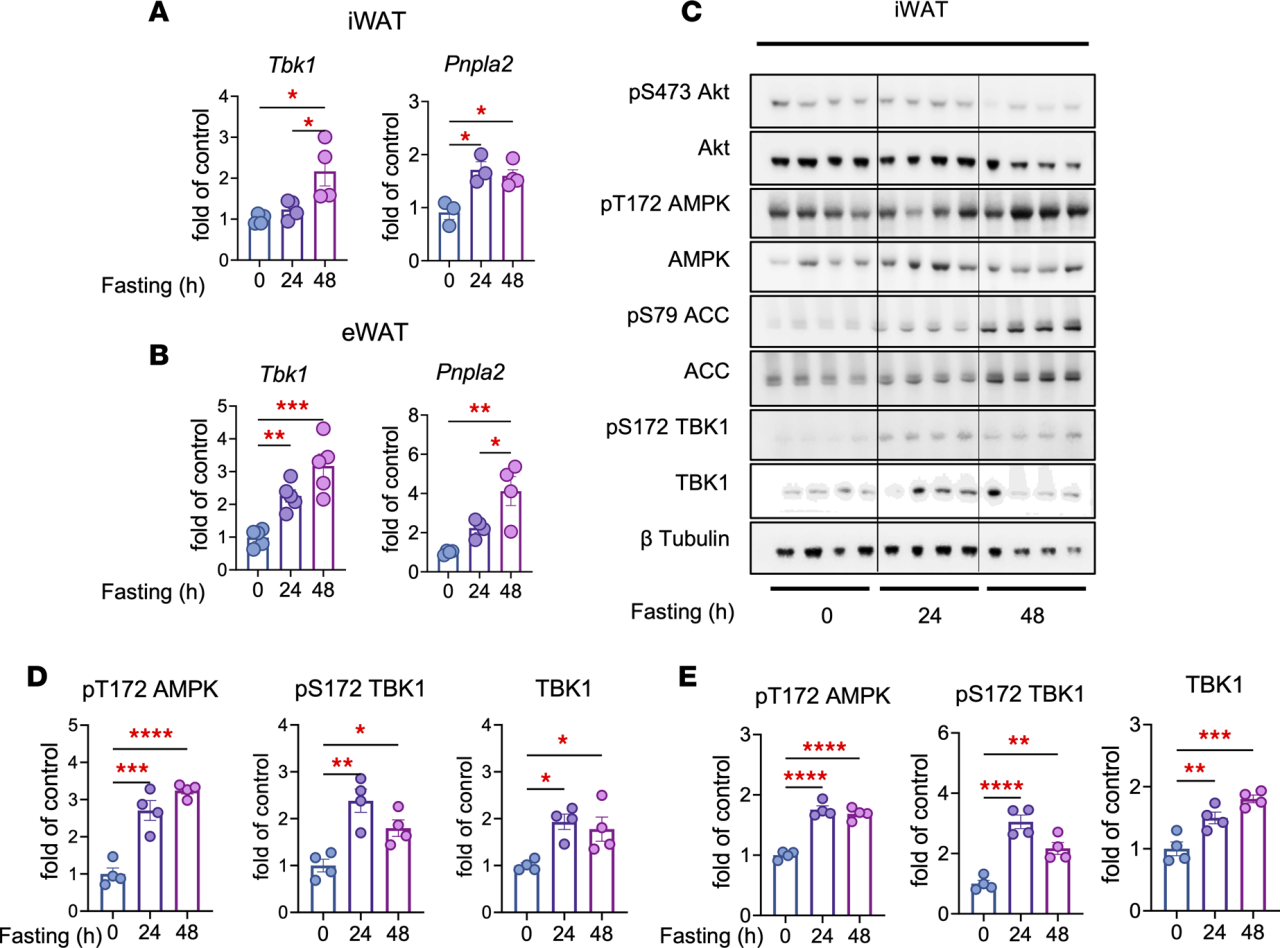
Fasting induces TBK1 at both the transcriptional and protein levels. (**A**) qPCR analysis of *Tbk1* and *Pnpla2* mRNA in iWAT from mice fed a normal diet (ND) or an HFD and fasted for 24 or 48 hours. *n* = 3–4, 1-way ANOVA with Tukey’s multiple-comparison test. (**B**) qPCR analysis of *Tbk1* and *Pnpla2* mRNA in eWAT from ND and HFD mice fasted for 24 or 48 hours. *n* = 4, 1-way ANOVA with Tukey’s multiple-comparison test. (**C**) Immunoblot analysis of iWAT from ND mice fasted for 0, 24, or 48 hours, probed for pS473 Akt, Akt, pT172 AMPK, AMPK, pS79 ACC, ACC, pS172 TBK1, TBK1, and β-tubulin. (**D** and **E**) Quantification of immunoblots from iWAT (**D**) and eWAT (**E**). *n* = 4, 1-way ANOVA with Tukey’s multiple-comparison test. Data are presented as mean ± SEM; each dot represents a biological replicate. *P < 0.05, **P < 0.01, ***P < 0.001, ****P < 0.0001.

Obesity-induced inflammation increases TBK1 expression and activity in adipocytes, which suppresses energy expenditure and lipid mobilization (11). Interestingly, fasting also induces *Tbk1* in adipose tissue and the liver (11, 14), suggesting that TBK1 operates at both ends of the metabolic stress spectrum. To test whether fasting regulates *Tbk1* expression in adipose tissue, we measured its mRNA levels in inguinal white adipose tissue (iWAT) and epididymal white adipose tissue (eWAT) after 24–48 hours of fasting. *Tbk1* expression was significantly increased in both depots, accompanied by induction of the lipolysis gene *Pnpla2* (Figure 1, A and B). Immunoblot analysis further confirmed that fasting increased TBK1 phosphorylation at Ser172 and total TBK1 protein levels, together with the expected increase in AMPK phosphorylation at Thr172 (Figure 1, C–E, and Supplemental Figure 1A; supplemental material available online with this article; https://doi.org/10.1172/jci.insight.200168DS1).

**Figure 2:**
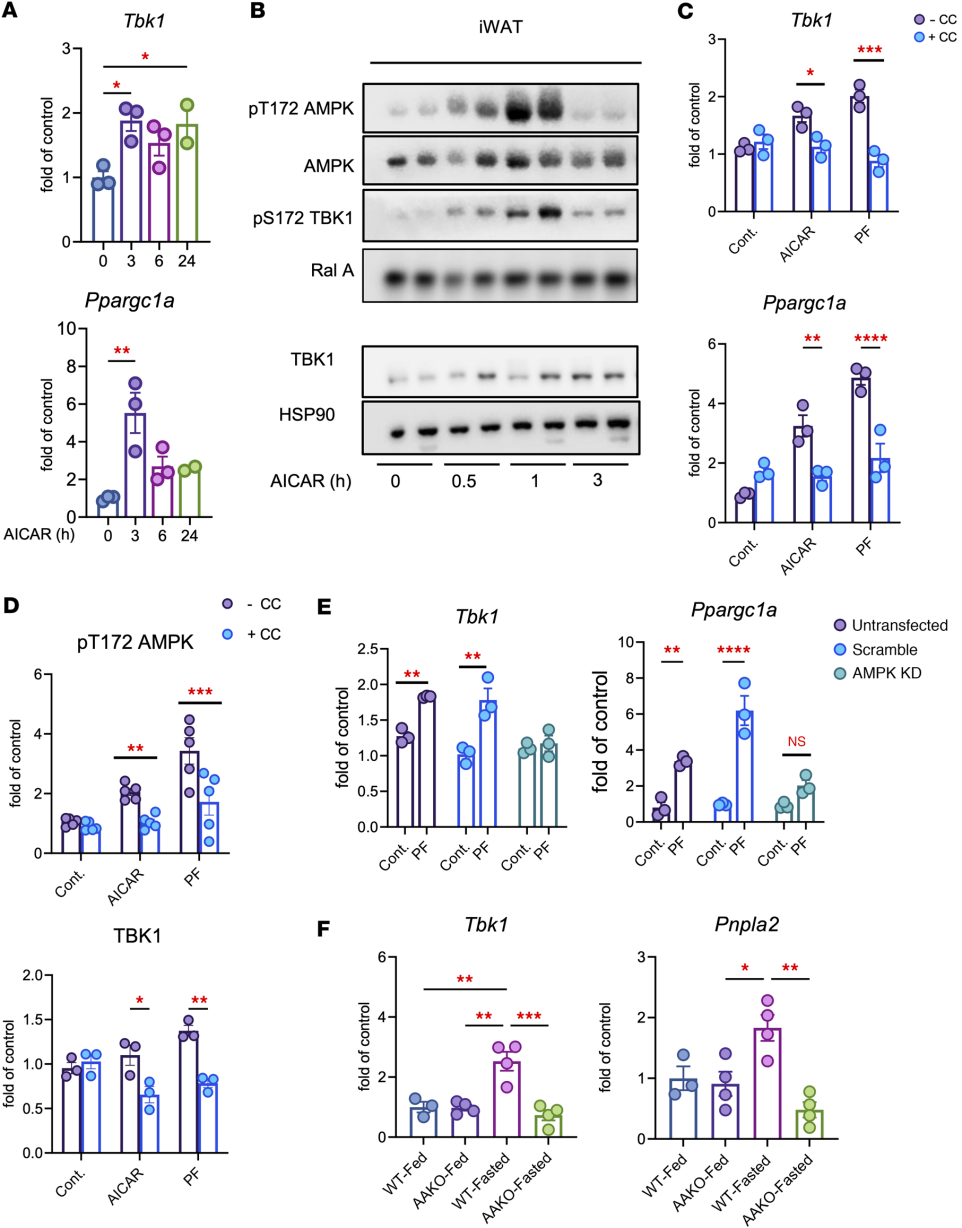
Fasting induces *Tbk1* in adipose tissue through AMPK. (**A**) qPCR analysis of *Tbk1* and *Ppargc1a* mRNA in iWAT from ND mice treated with 500 mg/kg AICAR (i.p.) for 0, 3, 6, or 24 hours. *n* = 2–3, 1-way ANOVA with Tukey’s multiple-comparison test. (**B**) Immunoblot analysis of iWAT from ND mice treated with 500 mg/kg AICAR (i.p.) for 0.5, 1, or 3 hours, probed for pS79 ACC, ACC, pT172 AMPK, AMPK, pS172 TBK1, TBK1, and HSP90. (**C** and **D**) Differentiated 3T3-L1 adipocytes were pretreated with 10 μM Compound C for 30 minutes followed by 10 μM PF-739 or 500 μM AICAR for 6 hours. qPCR analysis of *Tbk1* and *Ppargc1a* (**C**) and quantification of pT172 AMPK/AMPK and TBK1/HSP90 (**D**) are shown. *n* = 3, 2-way ANOVA with Tukey’s multiple-comparison test. (**E**) Differentiated 3T3-L1 adipocytes were transfected with siRNA targeting AMPKα1 and α2 for 3 days, then stimulated with 10 μM PF-739 for 6 hours. qPCR analysis of *Tbk1* and *Ppargc1a* is shown. *n* = 3, 2-way ANOVA with Tukey’s multiple-comparison test. (**F**) iWAT from *Prkaa1/2^AKO^* (AAKO) mice fasted for 48 hours was analyzed for *Tbk1* and *Pnpla2* mRNA expression. *n* = 3–4, 1-way ANOVA with Tukey’s multiple-comparison test. Data are presented as mean ± SEM; each dot represents a biological replicate. *P < 0.05, **P < 0.01, ***P < 0.001, ****P < 0.0001.

To determine whether AMPK activation contributes to fasting-induced *Tbk1*, we treated mice with the AMPK agonist AICAR. AICAR administration markedly increased *Tbk1* and *Ppargc1a* mRNA in iWAT and induced phosphorylation of both AMPK and TBK1 (Figure 2, A and B). In differentiated 3T3-L1 adipocytes, pharmacological AMPK activation with PF-739 or nutrient deprivation also increased *Tbk1* expression in a time-dependent manner (Supplemental Figure 1B). Immunoblot analysis of PF-739–treated adipocytes confirmed these findings, showing increased AMPK phosphorylation at Thr172; phosphorylation of its downstream targets pS79 ACC, pS555 ULK1, and pS792 raptor; and elevated TBK1 phosphorylation and protein levels (Supplemental Figure 1, C and D). Next, we utilized primary preadipocytes differentiated in vitro from WT and *Tbk1*^AKO^ mice treated with or without the AMPK activator PF-739 for 6 hours. AMPK activation led to a time-dependent increase in *Tbk1* expression and TBK1 protein levels in WT cells. Consistent with its role as a nutrient-responsive brake, the genetic deletion of TBK1 in adipocytes enhanced and sustained AMPK signaling. *Tbk1*^AKO^ adipocytes exhibited markedly higher levels of pT172 AMPK and its direct downstream targets, pS79 ACC and pS792 raptor, at multiple time points compared with WT controls (Supplemental Figure 1, E and F).

In 3T3-L1 adipocytes, pharmacological AMPK activation by either AICAR or PF-739 increased *Tbk1* and *Ppargc1a* expression, whereas pretreatment with the AMPK inhibitor Compound C abolished these effects (Figure 2, C and D, and Supplemental Figure 2, A and B). Consistently, AMPKα1/α2 knockdown by siRNA prevented PF-739–induced *Tbk1* expression (Figure 2E and Supplemental Figure 2C). Finally, fasting failed to induce *Tbk1* or *Pnpla2* expression in iWAT from adipocyte-specific AMPK-KO (*Prkaa1/2^AKO^*) mice (Supplemental Figure 2D), demonstrating that AMPK is required for fasting-mediated induction of Tbk1 (Figure 2F). Together, these results demonstrate that fasting upregulates *Tbk1* expression in adipose tissue and that this response is dependent on AMPK activation.

### The PGC1α/NRF1 axis mediates AMPK-dependent Tbk1 transcription during nutrient stress.

**Figure 3:**
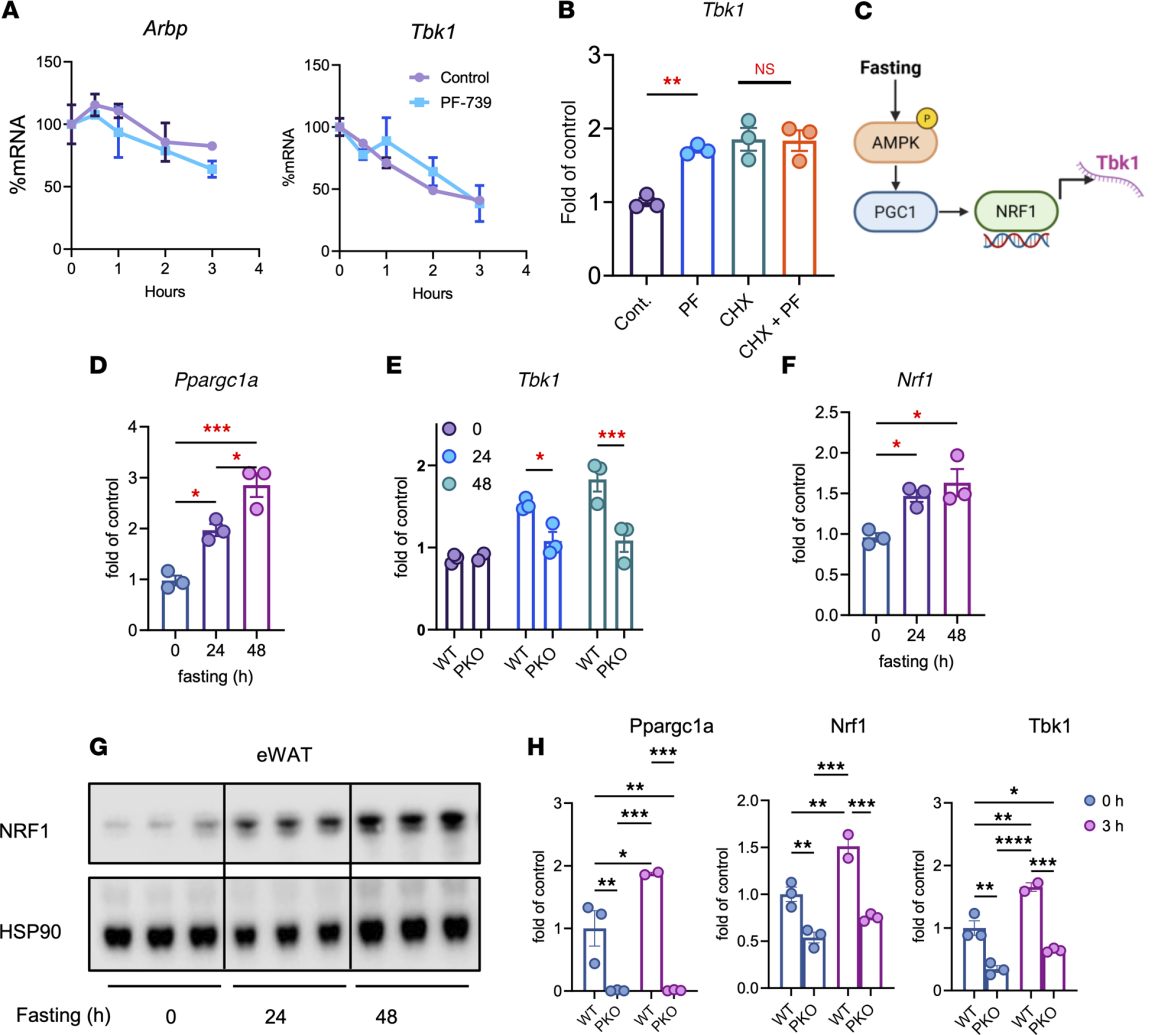
PGC1α is required for the transcriptional induction of *Tbk1* during fasting. (**A**) mRNA stability assay of *Arbp* and *Tbk1* in 3T3-L1 adipocytes treated with actinomycin D in the presence or absence of 10 μM PF-739. (**B**) qPCR analysis of *Tbk1* mRNA in 3T3-L1 adipocytes treated with cycloheximide (CHX) or PF-739 for 6 hours to assess the requirement for de novo protein synthesis. *n* = 3, 1-way ANOVA with Tukey’s multiple-comparison test. (**C**) Schematic model of fasting-induced *Tbk1* transcription via the AMPK/PGC1α/NRF1 signaling axis. (**D**) qPCR analysis of *Ppargc1a* in eWAT from lean mice fasted for 24–48 hours. *n* = 3, 1-way ANOVA with Tukey’s multiple-comparison test. (**E**) qPCR analysis of *Tbk1* mRNA in eWAT from WT and adipocyte-specific *Ppargc1a*-KO (*Ppargc1a*^AKO^; PKO) mice fasted for 24–48 hours. *n* = 3, 2-way ANOVA with Tukey’s multiple-comparison test. (**F** and **G**) qPCR (**F**) and immunoblot analysis (**G**) of NRF1 in eWAT from mice fasted for 24–48 hours. *n* = 3, 1-way ANOVA with Tukey’s multiple-comparison test. (**H**) qPCR analysis of *Ppargc1a*, *Nrf1*, and *Tbk1* mRNA in iWAT from WT and *Ppargc1a*^AKO^ mice 3 hours after a single acute injection of AICAR (500 mg/kg, i.p.). *n* = 2–3, 2-way ANOVA with Tukey’s multiple-comparison test. Data are presented as mean ± SEM; each dot represents a biological replicate. *P < 0.05, **P < 0.01, ***P < 0.001, ****P < 0.0001.

To explore the mechanism by which AMPK activation increases *Tbk1* mRNA levels, we first examined mRNA stability in isolated adipocytes. PF-739 did not alter the half-life of *Tbk1* mRNA, suggesting that AMPK regulates transcription of *Tbk1* mRNA rather than its stability (Figure 3A). Treatment of cells with cycloheximide blocked PF-739–induced *Tbk1* expression, indicating that de novo protein synthesis is required (Figure 3B). These findings led us to hypothesize that AMPK controls *Tbk1* transcription through a PGC1α/NRF1 module (Figure 3C). Under the same conditions that induced *Tbk1*, both PF-739 and nutrient deprivation also increased *Ppargc1a* expression in a time-dependent manner in 3T3-L1 adipocytes (Supplemental Figure 3, A and B), while fasting elevated *Ppargc1a* expression in eWAT (Figure 3D). To explore the importance of PGC1a in *Tbk1* expression, we created adipocyte-specific PGC1a-KO (*Ppargc1a^AKO^)* mice. *Tbk1* mRNA levels were not elevated in response to fasting in these mice compared with WT controls (Figure 3E). Similarly, PF-739 treatment did not increase *Tbk1* expression in *Ppargc1a^AKO^* primary preadipocytes differentiated in vitro, confirming that PGC1α is required for AMPK-dependent *Tbk1* transcription (Supplemental Figure 3C).

NRF1 is a known binding partner of PGC1α and responds to a higher AMP/ATP ratio (20). In silico promoter analysis revealed a putative NRF1 binding motif within the *Tbk1* locus (Supplemental Figure 3D). Public ChIP-seq data (NCBI Gene Expression Omnibus GSM1232709, NCBI Sequence Read Archive SRX352245) further showed NRF1 occupancy at this site in 3T3-L1 adipocytes (21) (Supplemental Figure 3E). In mice, fasting increased Nrf1 mRNA and protein in eWAT (Figure 3, F and G, and Supplemental Figure 3F). Although acute AICAR treatment (500 mg/kg, i.p.) induced *Ppargc1a*, *Nrf1*, and *Tbk1* expression in iWAT from WT mice, this response was markedly attenuated in *Ppargc1a^AKO^* mice (Figure 3H). These results demonstrate that PGC1α is required for the transcriptional induction of *Tbk1* during nutrient deprivation.

**Figure 4:**
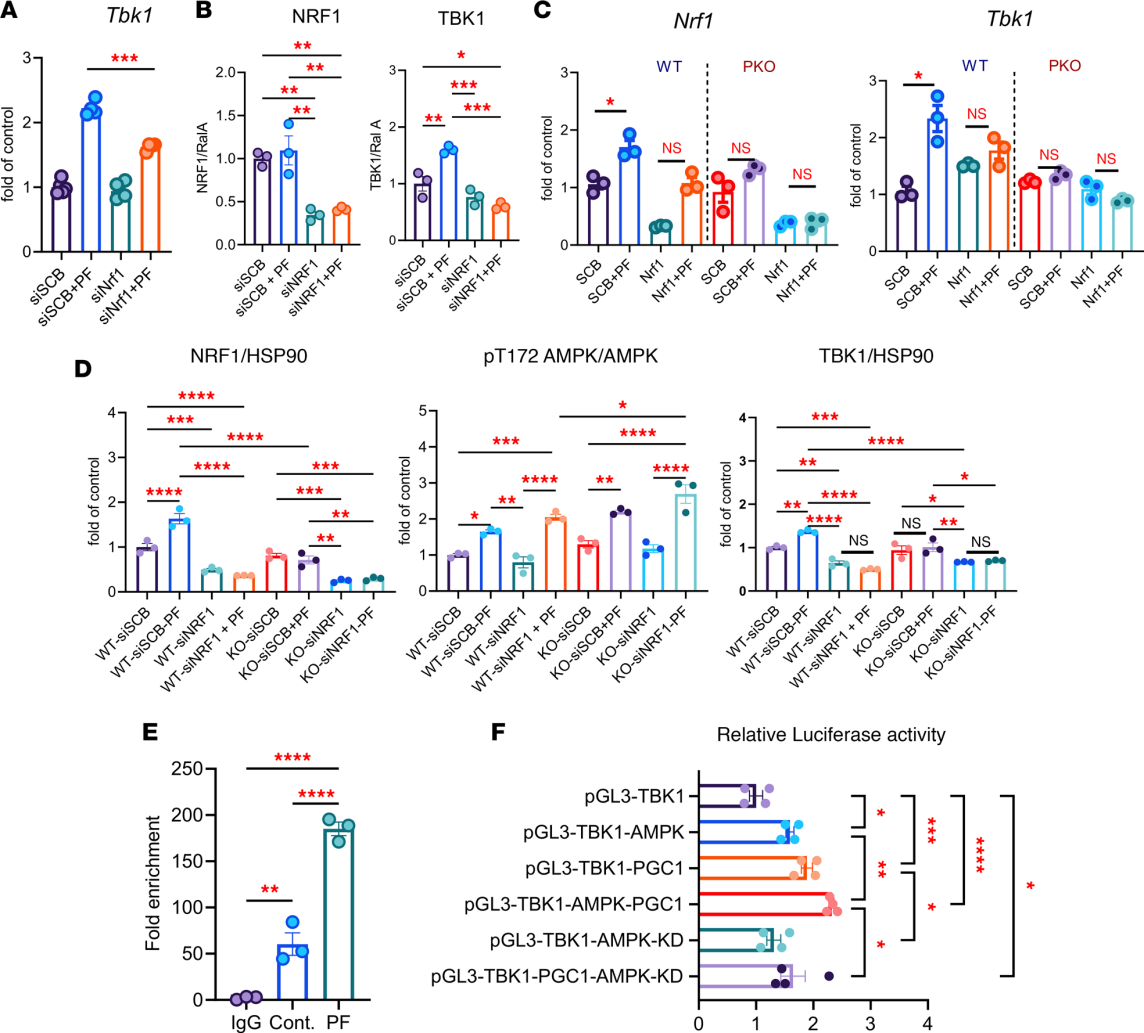
NRF1 acts as the downstream transcriptional effector of AMPK and PGC1α to activate *Tbk1* in adipocytes. (**A**) qPCR analysis of *Tbk1* mRNA (*n* = 4–5) and (**B**) quantitative Western blot analysis of NRF1 and TBK1 (*n* = 3) from 3T3-L1 adipocytes transfected with scramble or *Nrf1* siRNA and treated with 10 μM PF-739 for 6 hours. One-way ANOVA with Tukey’s multiple-comparison test. (**C**) qPCR analysis of *Nrf1* and *Tbk1* mRNA (*n* = 3) (**D**) and quantitative immunoblot analysis of NRF1, pT172 AMPK, and TBK1 protein (L) in PPDIVs from WT and PKO mice transfected with *Nrf1* siRNA and treated with 10 μM PF-739. *n* = 3, 1-way ANOVA with Tukey’s multiple-comparison test. (**E**) ChIP-qPCR analysis of NRF1 binding to the *Tbk1* promoter region containing the predicted NRF1 motif in 3T3-L1 adipocytes. *n* = 3*,* 1-way ANOVA with Tukey’s multiple-comparison test. (**F**) Dual-luciferase reporter assay in HEK293T cells transfected with a reporter plasmid containing the WT *Tbk1* promoter (incorporating the NRF1-binding site) and cotransfected with plasmids encoding AMPK, kinase-dead AMPK (KD), and/or PGC1α. *n* = 4, 1-way ANOVA with Tukey’s multiple-comparison test. Data are presented as mean ± SEM; each dot represents a biological replicate. *P < 0.05, **P < 0.01, ***P < 0.001, ****P < 0.0001.

To test whether NRF1 is necessary for *Tbk1* induction, we silenced *Nrf1* in 3T3-L1 adipocytes. *Nrf1* knockdown reduced PF-739–induced *Tbk1* mRNA and TBK1 protein expression without affecting *Ppargc1a* levels (Figure 4, A and B, and Supplemental Figure 3G). Furthermore, NRF1 depletion decreased TBK1 protein levels and increased pS79 ACC, consistent with reduced TBK1-mediated feedback inhibition of AMPK (Supplemental Figure 3H). *Nrf1* silencing blunted *Tbk1* induction in WT cells and completely abolished it in PPDIVs from *Ppargc1a^AKO^* mice treated with PF-739, demonstrating that both PGC1α and NRF1 are required for AMPK-dependent *Tbk1* transcription (Figure 4C and Supplemental Figure 3I). Immunoblot analysis further confirmed that TBK1 protein induction was absent in *Ppargc1a^AKO^* PPDIVs, *Nrf1*-silenced PPDIVs, and *Ppargc1a^AKO^* PPDIVs transfected with *siNrf1* (Figure 4D and Supplemental Figure 3J). When TBK1 was suppressed, pT172 AMPK and pS79 ACC were increased (Figure 4D and Supplemental Figure 3J), indicating a reciprocal feedback loop between AMPK and TBK1 operating at both the transcriptional and translational levels.

ChIP-qPCR analysis confirmed NRF1 occupancy at the predicted motif within the *Tbk1* promoter, which increased after nutrient deprivation (Figure 4E). To establish the functional relevance of this binding, the *Tbk1* promoter region containing the NRF1-binding site was cloned into a pGL3-basic luciferase reporter vector (pGL3-TBK1) and cotransfected into HEK293T cells with plasmids encoding WT AMPK, kinase-dead AMPK, and/or PGC1α. Overexpression of WT AMPK and PGC1α increased luciferase activity, whereas coexpression of the AMPK–kinase-dead mutant abolished this induction (Figure 4F). These results identify a nutrient-sensitive AMPK/PGC1α/NRF1 transcriptional axis as the primary mechanism driving *Tbk1* expression in adipocytes during energy deprivation.

### Obesity disrupts fasting-induced AMPK/TBK1 signaling in adipose tissue.

**Figure 5:**
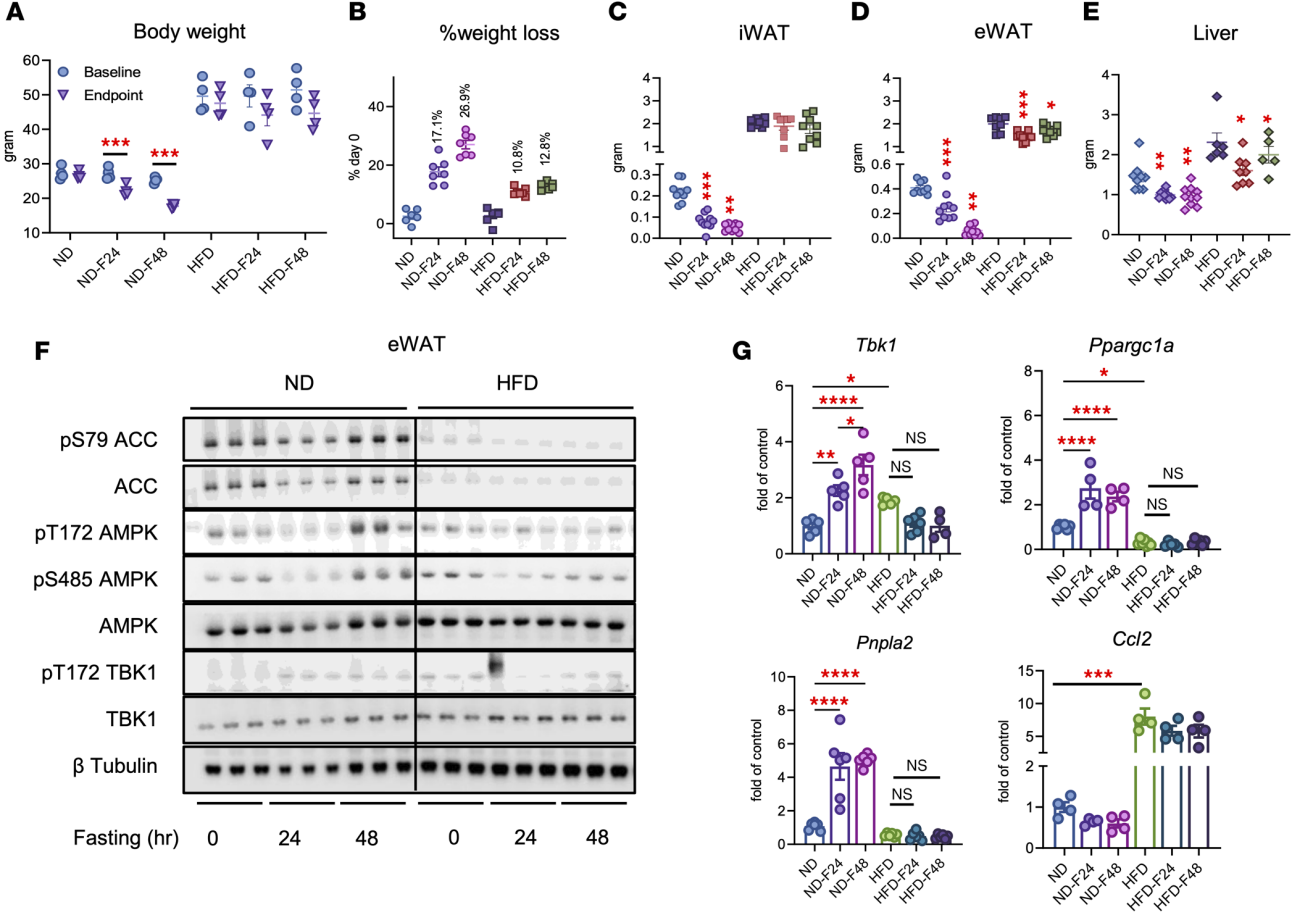
Obesity blunts the AMPK/TBK1 feedback loop during fasting. (**A** and **B**) Body weight (**A**) and percentage weight loss (**B**) of ND and HFD mice after 24–48 hours fasting. *n* = 4–7, 1-way ANOVA with Tukey’s multiple-comparison test. (**C**–**E**) iWAT (**C**), eWAT (**D**), and liver (**E**) weights from the same cohort. *n* = 5–10, 1-way ANOVA with Tukey’s multiple-comparison test. (**F**) Immunoblot analysis of iWAT from ND and HFD mice fasted for 0, 24, or 48 hours, probed for pS79 ACC, ACC, pT172 AMPK, pS485 AMPK, AMPK, pS172 TBK1, TBK1, and β-tubulin. (**G**) qPCR analysis of *Tbk1*, *Ppargc1a*, *Pnpla2*, and *Ccl2* mRNA in eWAT from ND and HFD mice fasted for 24–48 hours. *n* = 4–6, 1-way ANOVA with Tukey’s multiple-comparison test. Data are presented as mean ± SEM; each dot represents a biological replicate. *P < 0.05, **P < 0.01, ***P < 0.001, ****P < 0.0001.

To test whether obesity alters fasting responses in adipose tissue, we compared mice fed a normal or high-fat diet (HFD) and then subjected them to 24–48 hours of fasting. Lean mice showed a pronounced reduction in body weight during fasting, whereas obese mice exhibited only a modest decline (Figure 5, A and B). iWAT, eWAT, and liver mass decreased in both groups, but the extent of loss was consistently greater in lean mice (Figure 5, C–E).

**Figure 6:**
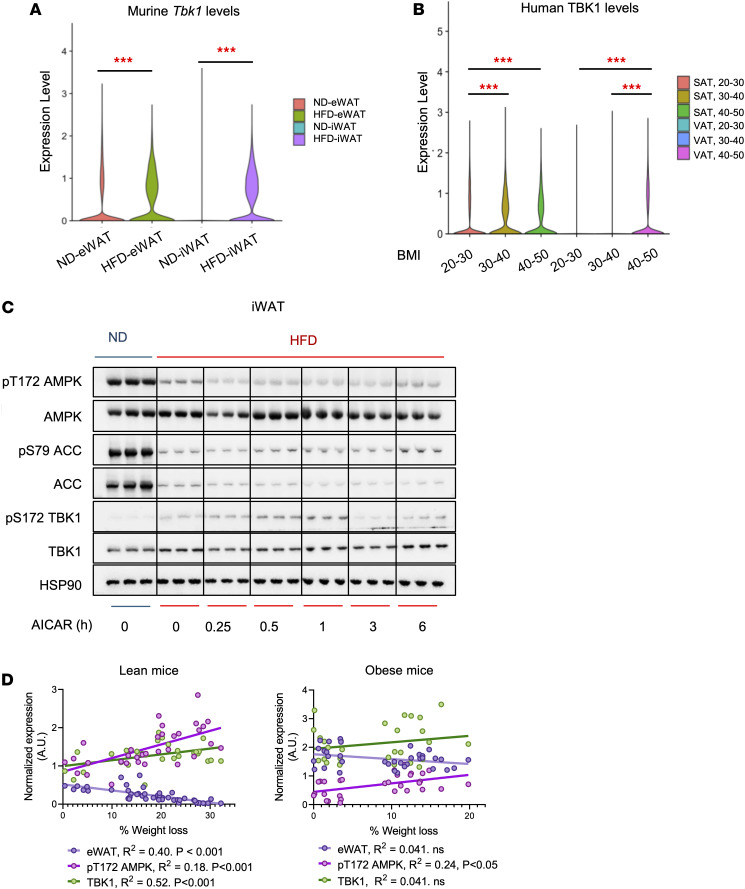
*Tbk1* expression in murine and human adipocytes correlates with obesity and metabolic status. (**A**) Single-cell RNA-seq analysis of mature adipocytes isolated from eWAT and iWAT of ND- and HFD-fed mice showing murine *Tbk1* expression. Statistical significance was determined using the Wilcoxon rank-sum test with FDR adjustment. (**B**) Single-cell RNA-seq analysis of human adipocytes from subcutaneous adipose tissue (SAT) and visceral adipose tissue (VAT) across individuals with varying BMI (20–30, 30–40, and 40–50). Statistical significance was determined using the Wilcoxon rank-sum test with FDR adjustment. (**C**) Immunoblot analysis of iWAT from ND and HFD mice treated with AICAR for the indicated times, probed for pT172 AMPK, AMPK, pS79 ACC, ACC, pS172 TBK1, TBK1, and HSP90. (**D**) Correlation of pT172 AMPK, TBK1, and eWAT mass with percentage weight loss in lean and obese mice. Expression levels of pT172 AMPK and TBK1 were quantified by Western blot. Each data point represents an individual mouse. The *R*^2^ and *P* values were determined using simple linear regression. Data are presented as mean ± SEM; each dot represents a biological replicate ***P < 0.001.

We next examined the signaling pathways underlying these differences. Fasting robustly activated AMPK in eWAT from lean mice, as indicated by increased pT172 AMPK and pS79 ACC, and simultaneously induced TBK1 phosphorylation and protein expression (Figure 5F). In contrast, fasting failed to fully activate AMPK in obese mice, while basal pS172 TBK1 and TBK1 protein remained elevated, suggesting a shift in the AMPK-TBK1 balance (Figure 5F). Consistent with these findings, qPCR analysis showed that fasting induced *Tbk1*, *Ppargc1a*, and *Pnpla2* in lean but not obese mice, although obese adipose tissue displayed higher basal *Tbk1*, in line with inflammation-associated induction. Notably, the inflammatory chemokine *Ccl2* was suppressed by fasting in lean mice but remained unchanged in obese mice (Figure 5G). Single-cell RNA-seq of murine mature adipocytes confirmed this observation, revealing elevated murine *Tbk1* expression in adipocytes from the eWAT of obese mice (Figure 6A). We next examined human *TBK1* expression in adipocytes from subcutaneous and visceral adipose tissue across individuals with varying BMI. Consistent with findings in HFD-fed mice, human *TBK1* expression was increased in individuals with higher BMI in both subcutaneous and visceral adipose tissue (Figure 6B). These results suggest that chronic inflammation associated with obesity sustains TBK1 expression while suppressing the transcriptional program normally activated by fasting.

To assess whether this impaired response reflected a loss of AMPK function, we treated mice with AICAR (500 mg/kg, i.p.). Lean mice showed strong activation of AMPK signaling, whereas obese mice exhibited attenuated responses, with reduced pT172 AMPK and pS79 ACC but persistently high basal pS172 TBK1 and TBK1 (Figure 6C). These findings indicate that chronic TBK1 elevation in obesity antagonizes AMPK activity and limits the catabolic adaptation to fasting.

Finally, correlational analyses revealed functional consequences of the disrupted feedback loop in this imbalance. In lean mice, pT172 AMPK strongly correlated with the magnitude of body mass reduction and inversely with eWAT mass. TBK1 expression also increased in proportion to weight loss, in line with a feedback loop engaged during fasting (Figure 6D). In obese mice, pT172 AMPK correlated with weight loss, but to a lesser extent than in lean mice. Moreover, the relationship between eWAT mass loss and TBK1 expression was diminished in obesity (Figure 6D), suggesting that inflammation-driven TBK1 elevation and impaired AMPK activation contribute to the limited reduction in adiposity during fasting.

### TBK1 restrains AMPK activation and lipid mobilization during fasting.

We previously identified a feedback loop in which TBK1 restrains AMPK activity in adipose tissue under nutrient excess (11). To extend this model, we asked whether TBK1 also constrains adaptive responses to caloric restriction. Because fasting strongly activates AMPK, we hypothesized that TBK1 restrains fasting-induced lipid mobilization, and that inhibiting TBK1 would amplify AMPK signaling and promote fat catabolism. To test this, we first treated 3T3-L1 adipocytes with the TBK1 inhibitor amlexanox (13) during nutrient deprivation. Starvation increased pT172 AMPK and its target pS79 ACC, together with pS172 TBK1, in line with AMPK and TBK1 activation during energy stress. Amlexanox further enhanced pT172 AMPK and pS79 ACC while reducing pS172 TBK1, indicating that TBK1 inhibition augments AMPK signaling under starvation (Supplemental Figure 4, A and B).

**Figure 7:**
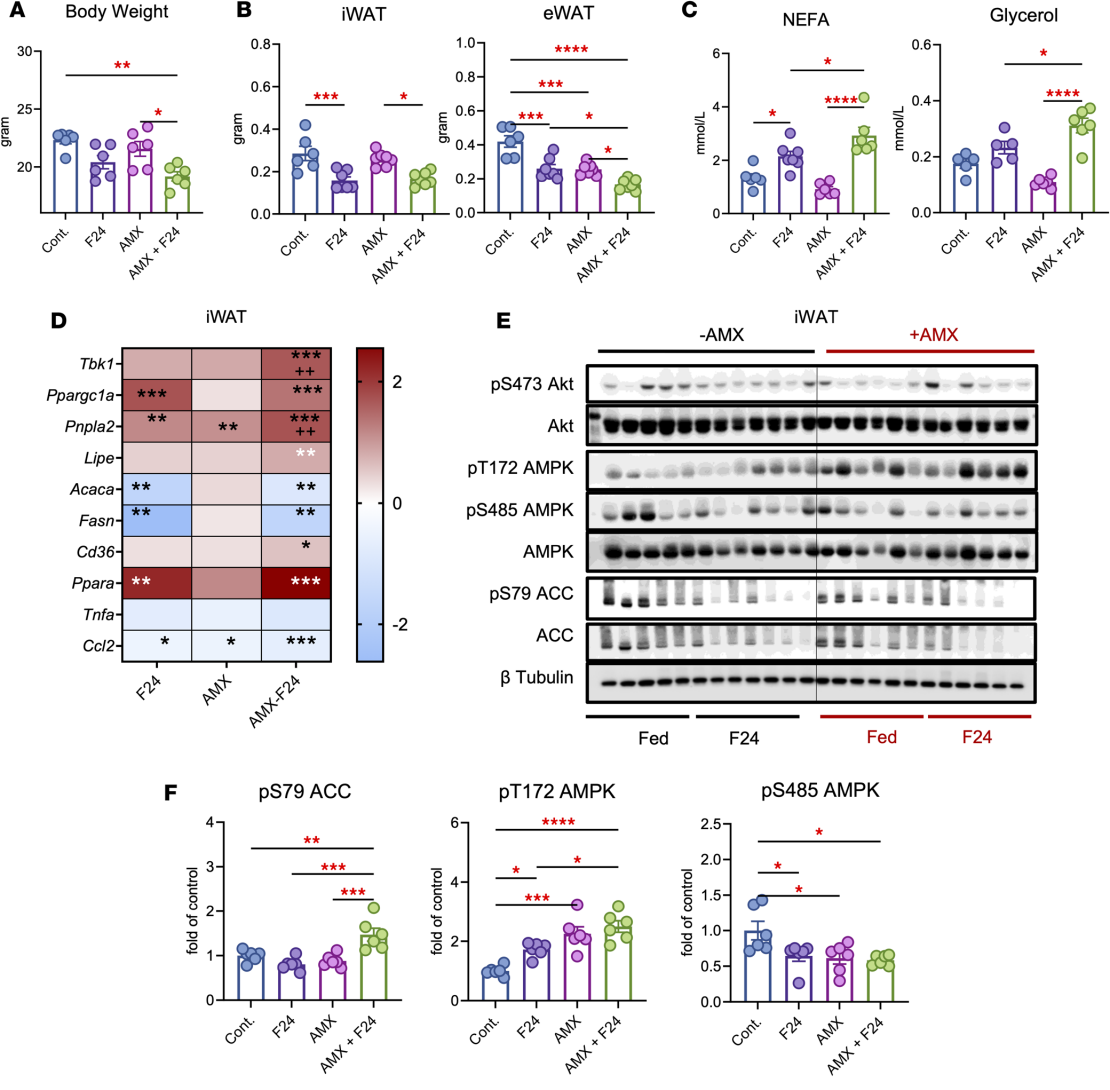
Inhibition of TBK1 by amlexanox promotes fat mass loss during fasting. (**A** and **B**) Body weight (**A**) and iWAT and eWAT mass (**B**) of lean mice treated with 25 mg/kg amlexanox (AMX) for 2 weeks with or without 24 hours of fasting. *n* = 6, 1-way ANOVA with Tukey’s multiple-comparison test. (**C**) Plasma non-esterified fatty acid (NEFA) and glycerol levels in the same cohort. *n* = 6, 1-way ANOVA with Tukey’s multiple-comparison test. (**D**) Heatmap of mRNA expression in iWAT of mice treated with 25 mg/kg AMX and subjected to 24 hours of fasting. Data are presented as log_2_ fold-change relative to control. *n* = 6, 1-way ANOVA with Tukey’s multiple-comparison test. (**E**) Immunoblot analysis of iWAT from lean mice treated with vehicle or AMX for 2 weeks and subjected to 24 hours of fasting, probed for pS473 Akt, Akt, pT172 AMPK, pS485 AMPK, AMPK, pS79 ACC, ACC, pS172 TBK1, TBK1, and β-tubulin. (**F**) Quantification of pS79 ACC/ACC, pT172 AMPK/AMPK, and pS485 AMPK/AMPK from **E**. *n* = 6, 1-way ANOVA with Tukey’s multiple-comparison test. Data are presented as mean ± SEM; each dot represents a biological replicate. *P < 0.05, **P < 0.01, ***P < 0.001, ****P < 0.0001.

We next evaluated these effects in vivo using lean mice treated with a suboptimal amlexanox dose at 25 mg/kg for 2 weeks. To exclude anorectic effects, pair-fed mice were included. Despite matched food intake, amlexanox-treated mice exhibited greater body weight reduction by day 6 (Supplemental Figure 5, A and B). After a 24-hour fast, amlexanox-treated mice lost more body weight and had lower iWAT and eWAT mass compared with controls (Figure 7, A and B). Under fed conditions, amlexanox lowered circulating NEFA and glycerol, together with suppression of basal lipolysis by AMPK. After fasting, however, both metabolites rose sharply, with amlexanox-treated mice showing the highest levels, indicating amplified lipolysis during energy stress (Figure 7C). Fasting induced *Tbk1*, *Pnpla2*, and *Lipe* in iWAT*,* with further induction in the amlexanox plus fasting group. Lipogenic genes *Acaca* and *Fasn*, and inflammatory markers *Tnfα* and *Ccl2*, were suppressed by fasting and further reduced with amlexanox. Meanwhile, *Ppargc1a*, and *Pparα* were strongly upregulated (Figure 7D and Supplemental Figure 5C). Western blotting confirmed that amlexanox enhanced fasting-induced AMPK activation, with higher pT172 AMPK, lower inhibitory pS485 AMPK, and increased pS79 ACC (Figure 7, E and F), consistent with reduced inflammatory signaling. Similar changes were observed in eWAT (Supplemental Figure 5, D and E).

**Figure 8:**
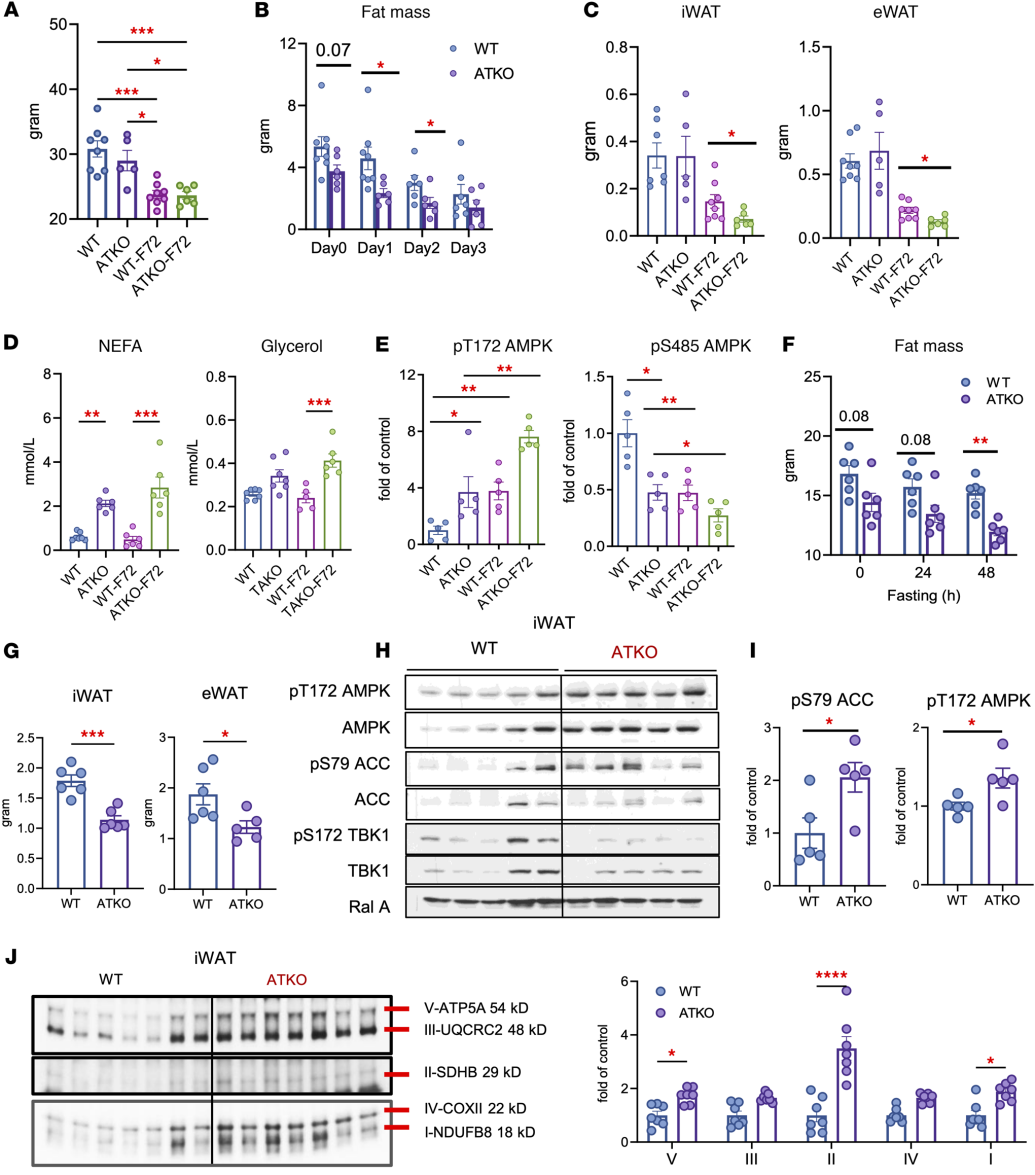
TBK1 loss in adipocytes promotes AMPK activation and mitochondrial OXPHOS expression under nutrient stress. (**A**–**C**) WT or ATKO mice subjected to prolonged fasting for 72 hours. Body weight (**A**), fat mass over 3 days of fasting (**B**), and iWAT/eWAT weights (**C**) are shown. *n* = 5–8, 1-way or 2-way ANOVA with Tukey’s multiple-comparison test. (**D**) Plasma NEFA and glycerol levels in WT and ATKO mice after 72 hours fasting. *n* = 5–8, 1-way ANOVA with Tukey’s multiple-comparison test. (**E**) Quantification of pT172 AMPK/AMPK and pS485 AMPK/AMPK in iWAT from fasted WT and ATKO mice. *n* = 5–8, 1-way ANOVA with Tukey’s multiple-comparison test. (**F** and **G**) WT and ATKO mice fed a 60% kcal HFD for 12 weeks and subjected to 48 hours of fasting. Fat mass was measured by EchoMRI (**F**), and iWAT/eWAT weights are shown (**G**). *n* = 6, 1-way ANOVA with Tukey’s multiple-comparison test. (**H**) Immunoblot analysis of iWAT from WT and ATKO mice fasted for 48 hours, probed for pS79 ACC, ACC, pT172 AMPK, AMPK, pS172 TBK1, TBK1, and RalA. (**I**) Quantification of pS79 ACC/ACC and pT172 AMPK/AMPK from **H**. *n* = 5, 1-way ANOVA with Tukey’s multiple-comparison test. (**J**) Immunoblot analysis and quantification of OXPHOS complexes (V–I) in iWAT from WT and ATKO mice fasted for 48 hours. *n* = 7*,* 1-way ANOVA with Tukey’s multiple-comparison test. Data are presented as mean ± SEM; each dot represents a biological replicate. **P < 0.01, ***P < 0.001, ****P < 0.0001. ATKO; *Tbk1^AKO^* mice.

To directly test the role of TBK1 in fasting adaptation, we subjected *Tbk1^AKO^* mice to prolonged fasting for 72 hours. Both WT and *Tbk1^AKO^* mice displayed comparable trends of body weight decline and maintained similar blood glucose levels (Figure 8A and Supplemental Figure 6, A and B). However, *Tbk1^AKO^* mice preserved lean mass but lost more fat mass than WT mice (Figure 8B and Supplemental Figure 6C), with reduced iWAT and eWAT depots and elevated NEFA and glycerol (Figure 8, C and D). *Tbk1^AKO^* mice also showed a decrease in liver mass and hepatic triglyceride accumulation during fasting (Supplemental Figure 6, D and E), indicating enhanced lipid mobilization and reduced hepatic lipid accumulation.

In WT mice, 72-hour fasting increased Tbk1 mRNA and protein, whereas *Tbk1^AKO^* mice showed no induction (Supplemental Figure 6F). *Tbk1^AKO^* mice exhibited higher basal pT172 AMPK, lower pS485 AMPK, and elevated pS79 ACC, and these differences were more pronounced after fasting (Figure 8E and Supplemental Figure 6, G and H). Under HFD conditions, *Tbk1^AKO^* mice fasted for 48 hours lost more body weight, iWAT, and eWAT, with only a modest reduction in lean mass compared with WT littermates (Figure 8, F and G, and Supplemental Figure 6, I and J). Immunoblot analysis of iWAT further confirmed that obese *Tbk1^AKO^* mice displayed higher pT172 AMPK accompanied by increased pS79 ACC (Figure 8, H and I), consistent with enhanced AMPK activity. In addition, fasted *Tbk1^AKO^* mice showed an increase in mitochondrial oxidative phosphorylation proteins, particularly Complex IV and V (Figure 8J), suggesting augmented oxidative capacity during fasting. These findings establish that TBK1 functions as a brake on AMPK during nutrient stress, restraining lipid mobilization and mitochondrial remodeling in adipose tissue to limit the depth of fasting-induced catabolism.

### Dual TBK1 inhibition and AMPK activation improve glucose metabolism and promote fat loss in obese mice.

TBK1 lies at the intersection of inflammatory and metabolic signaling; the kinase paradoxically promotes anabolic and antiinflammatory responses that suppress basal metabolism (11). Since AMPK activation alone often triggers compensatory mechanisms that limit sustained energy expenditure, we hypothesized that TBK1 inhibition may relieve this brake and synergize with AMPK to enhance catabolic programs. To test this, we first treated 3T3-L1 adipocytes with the TBK1 inhibitor amlexanox and the AMPK activator PF-739. Cotreatment enhanced the expression of AMPK target genes compared with either drug alone (Supplemental Figure 7A), suggesting that TBK1 inhibition potentiates AMPK-driven transcriptional responses. Immunoblot analysis confirmed that PF-739 increased AMPK phosphorylation at T172 in a time-dependent manner, and this effect was further amplified by amlexanox (Supplemental Figure 7, B and C), indicating TBK1 inhibition enhances AMPK activation under energy stress.

**Figure 9:**
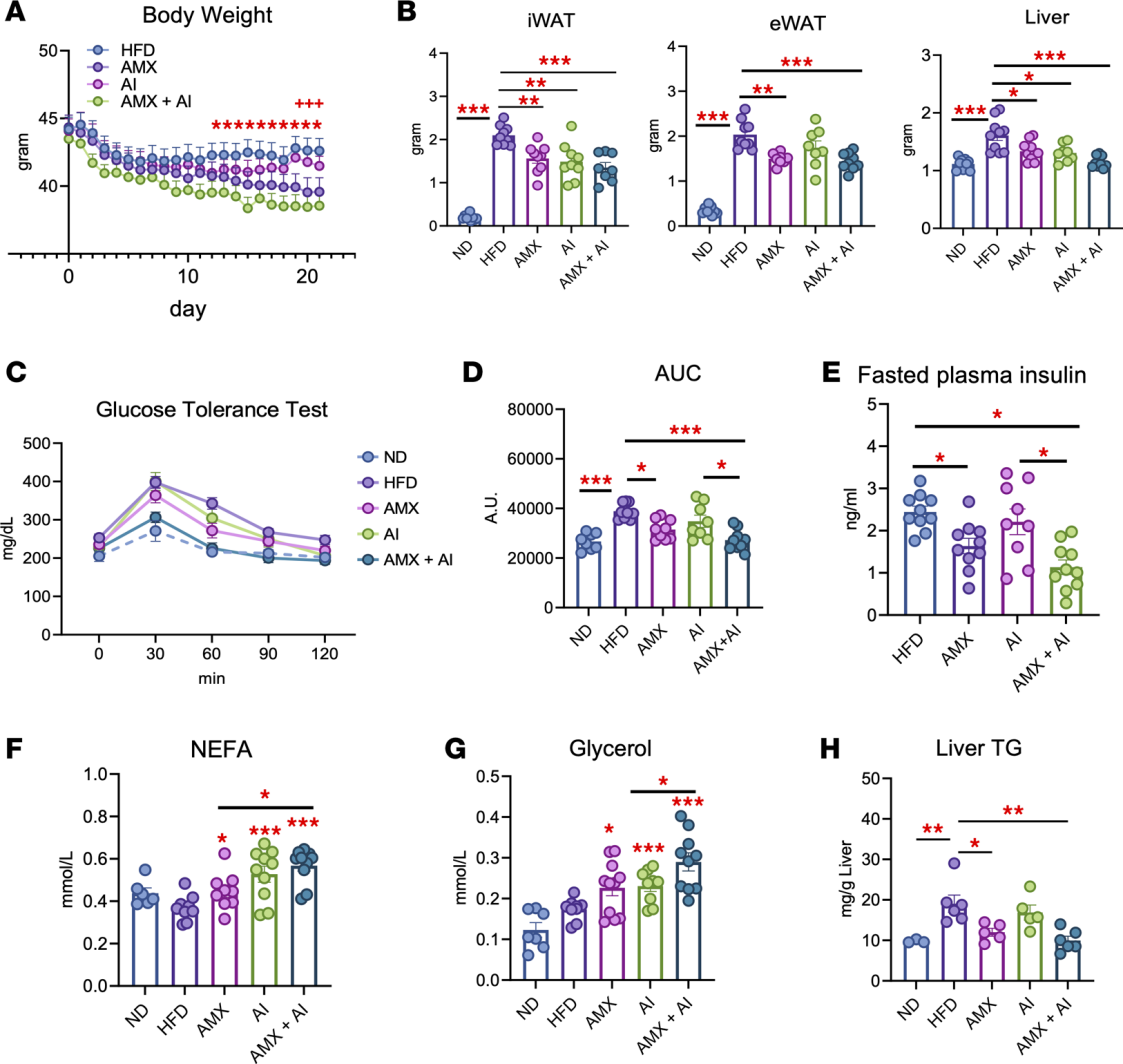
Combined TBK1 inhibition and AMPK activation improve metabolic health in obese mice. (**A**) HFD-fed mice treated with 25 mg/kg amlexanox (AMX) daily, 100 mg/kg AICAR (AI) i.p. every other day, or both for 21 days. Asterisks designate comparison with HFD-fed mice; plus signs designate comparison with AMX-treated mice. *n* = 8–10, 1-way ANOVA with Tukey’s multiple-comparison test. *P < 0.05 and ^+^*P* < 0.05. The plus signs indicate the same *P* value thresholds as the asterisks but represent the comparison against the AMX-treated group. (**B**) iWAT, eWAT, and liver weights at endpoint. *n* = 8–10*,* 1-way ANOVA with Tukey’s multiple-comparison test. (**C** and **D**) Glucose tolerance test (**C**) and corresponding AUC (**D**) in HFD-fed mice, showing improved glucose tolerance with AMX and most strongly with AMX plus AICAR. *n* = 8–12, 1-way ANOVA with Tukey’s multiple-comparison test. (**E**) Fasted plasma insulin measured by ELISA, reduced by AMX and further by AMX plus AICAR. *n* = 9–10*,* 1-way ANOVA with Tukey’s multiple-comparison test. (**F** and **G**) Plasma NEFA and glycerol levels, with greatest glycerol release in AMX plus AICAR. *n* = 7–10, 1-way ANOVA with Tukey’s multiple-comparison test. (**H**) Liver triglyceride content decreased by AMX and further reduced with AMX plus AICAR. *n* = 3–6, 1-way ANOVA with Tukey’s multiple-comparison test. Data are presented as mean ± SEM; each dot represents a biological replicate. **P < 0.01, ***P < 0.001, ****P < 0.0001.

To test this interaction in vivo, obese mice were treated with suboptimal doses of amlexanox and AICAR to reveal potential cooperative effects. Amlexanox was administered daily by oral gavage at 25 mg/kg, and AICAR was given every other day by i.p. injection at 100 mg/kg. To control for the confounding effect of food intake, non-amlexanox groups were pair-fed to match the reduced intake observed in amlexanox-treated mice, as shown in Supplemental Figure 8A. Daily weight monitoring showed that the combination-treated group began to lose weight earlier, starting at day 14, and exhibited more pronounced weight loss compared with single-agent treatments. Amlexanox alone led to weight reduction by day 18, whereas AICAR at this dose had minimal impact on body weight over the 21-day period (Figure 9A and Supplemental Figure 8B).

Tissue mass was measured at the study endpoint. Amlexanox plus AICAR reduced iWAT, eWAT, and liver mass compared with HFD controls, with stronger effects than amlexanox or AICAR alone (Figure 9B), indicating a potentiated reduction in adiposity and ectopic lipid accumulation. Glucose tolerance was modestly improved with amlexanox but markedly enhanced with the combination therapy (Figure 9C), accompanied by reduced AUC and fasting plasma insulin levels (Figure 9, D and E), suggesting improved systemic insulin sensitivity. Circulating NEFA and glycerol levels were measured to assess lipid mobilization. NEFA levels were moderately elevated with each treatment, and glycerol levels were highest in the amlexanox with AICAR group (Figure 9, F and G), indicating augmented lipolysis with combination therapy. Liver triglyceride content was also reduced by amlexanox and further decreased with the dual treatment (Figure 9H), consistent with improved hepatic lipid handling.

**Figure 10:**
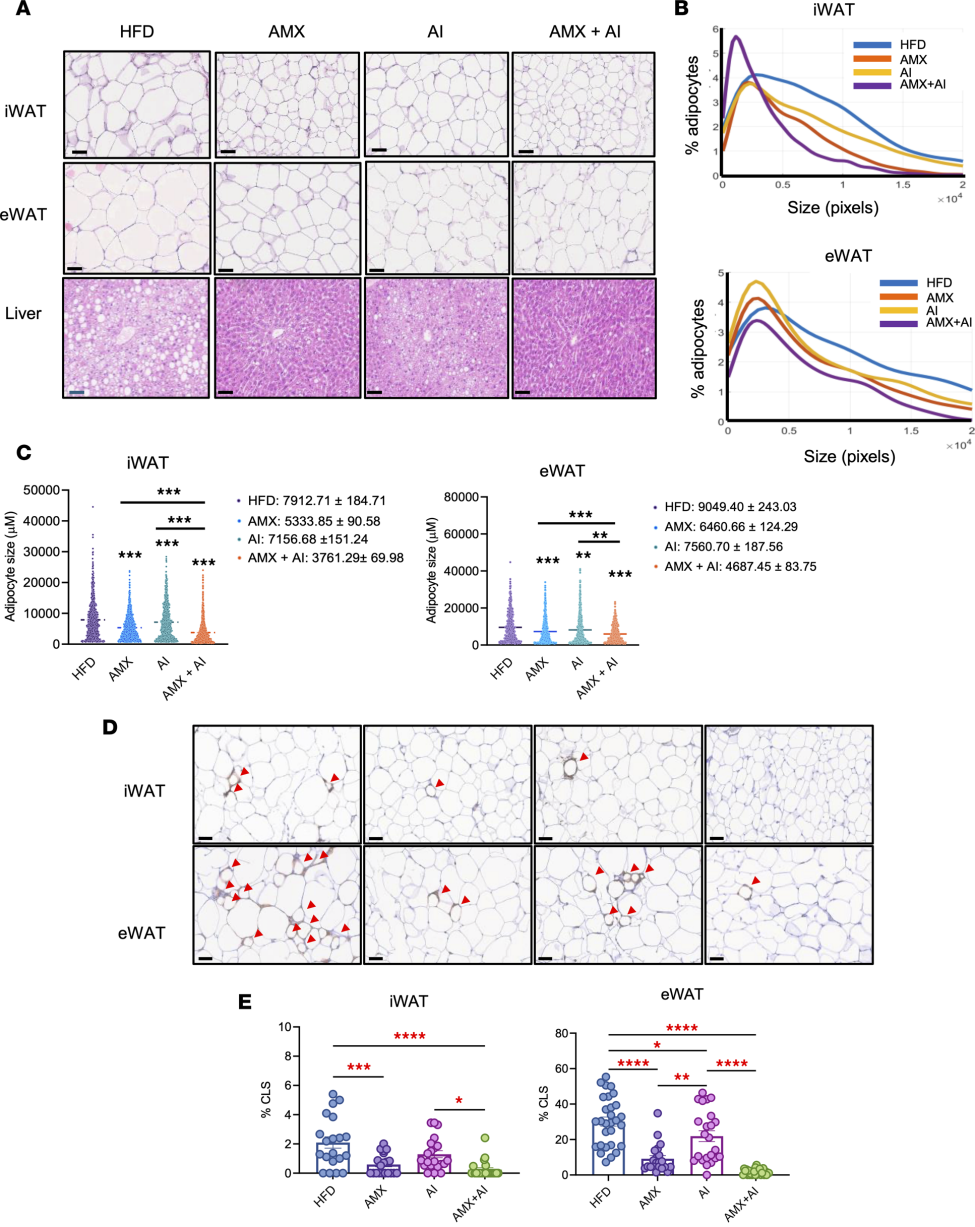
Combination therapy with amlexanox and AICAR reduces adipocyte hypertrophy and adipose tissue inflammation in obese mice. (**A**) Representative H&E staining of iWAT, eWAT, and liver. Scale bars: 60 μm. (**B**) Adipocyte size distribution shifted toward smaller adipocytes in AMX and AI, which is most prominent with AMX plus AICAR (*n* = 5). (**C**) Quantification of mean adipocyte size (μm). Adipocyte area was determined from at least 10 independent fields per animal (*n* = 6 mice per group). Numerical values for mean and SEM are indicated to the right of the distribution plots. (**D**) Representative images of iWAT and eWAT sections stained with anti-F4/80 antibody. Crown-like structures (CLSs) indicated by red arrowheads. Scale bars: 60 μm. (**E**) Quantification of the percentage of CLSs per field in iWAT and eWAT. CLSs were identified by F4/80-positive macrophage rings surrounding adipocytes and quantified in a blinded fashion across multiple sections per animal (*n* = 3 per group). At least 10 independent fields were analyzed per mouse. Data are presented as mean ± SEM. Statistical significance in **C** and **E** was determined by 1-way ANOVA with Tukey’s multiple-comparison test. *P < 0.05, **P < 0.01, ***P < 0.001, ****P < 0.0001.

Histological analysis revealed that HFD-fed mice exhibited adipocyte hypertrophy in WAT and steatosis in the liver (Figure 10A). Both amlexanox and AICAR reduced adipocyte size in iWAT and eWAT, with the greatest reduction observed in the combination group (Figure 10, B and C). Adipocyte size distribution shifted leftward, reflecting an increased population of smaller, metabolically healthy adipocytes (Figure 10B). In the liver, HFD feeding caused hepatocyte ballooning, lipid accumulation, and immune cell infiltration. Both amlexanox and AICAR partially improved hepatic morphology, and the combination treatment most effectively restored liver architecture, with fewer lipid droplets and preserved hepatocyte structure (Figure 10A), consistent with reduced liver triglycerides (Figure 9H).

Because chronic low-grade inflammation is a key feature of obesity-associated adipose dysfunction (22), we assessed macrophage infiltration in iWAT and eWAT by F4/80 IHC. HFD-fed mice exhibited numerous crown-like structures (CLSs), particularly in eWAT, reflecting high macrophage burden (Figure 10D). Amlexanox and AICAR each reduced CLS formation, and combination treatment almost eliminated CLSs in both iWAT and eWAT. Quantification confirmed reductions in the percentage of CLSs in all treatment groups, with the greatest suppression observed in the dual treatment group (Figure 10E), indicating cooperative antiinflammatory effects of TBK1 inhibition and AMPK activation. Together, these findings demonstrate that TBK1 inhibition potentiates AMPK activation to promote adipose tissue remodeling, enhance lipid mobilization, and improve systemic glucose and liver metabolic parameters in obese mice.

### Dual TBK1 inhibition and AMPK activation remodel metabolic signaling and suppress inflammation in adipose tissue and liver.

**Figure 11:**
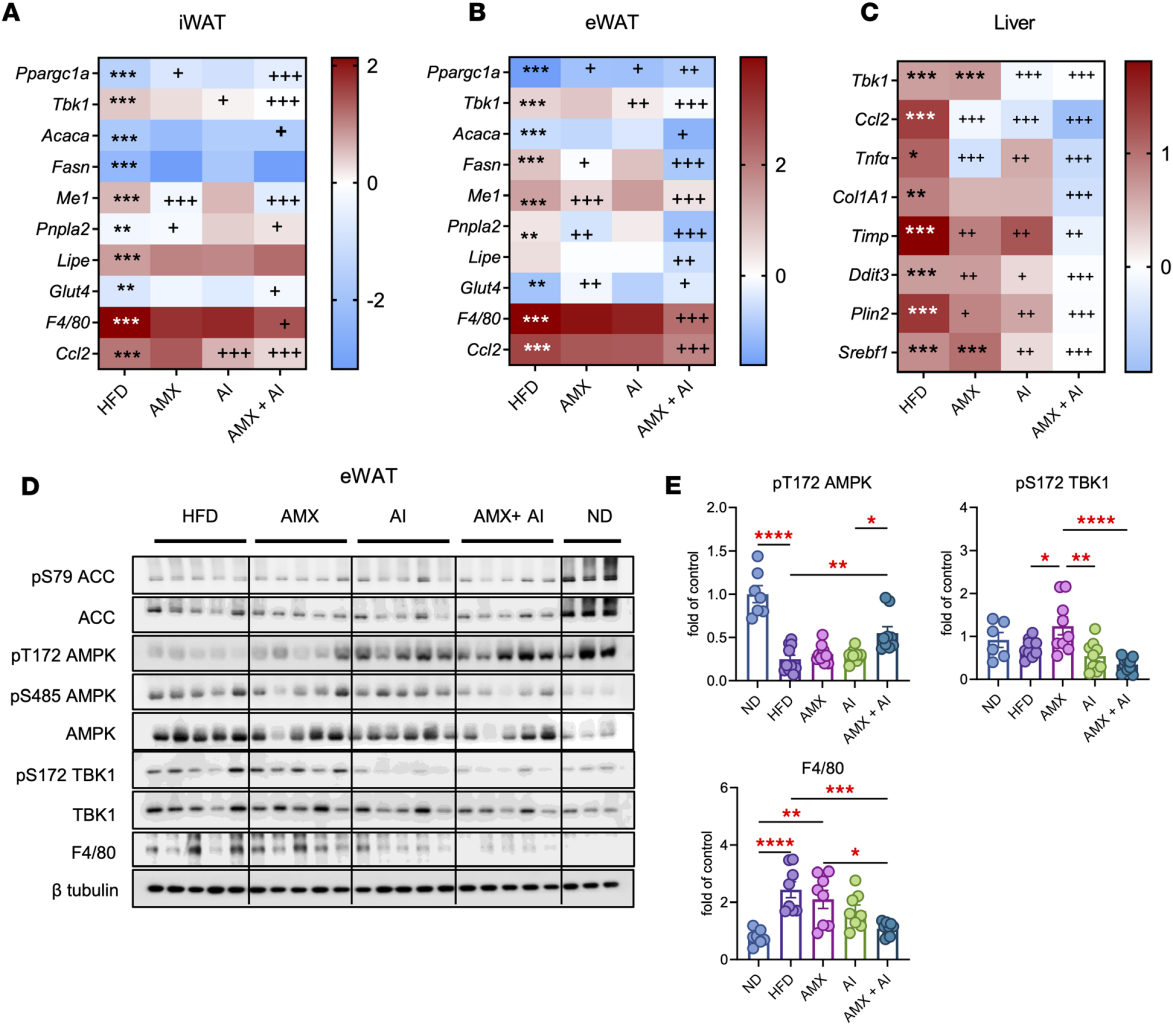
Amlexanox and AICAR combination treatment reprograms adipose and hepatic gene expression to reverse obesity-induced metabolic dysfunction. (**A** and **B**) Heatmap of gene expression in iWAT (**A**) and eWAT (**B**) from HFD-fed mice treated with 25 mg/kg amlexanox (AMX) daily, 100 mg/kg AICAR (AI) i.p. every other day, or both for 21 days. Genes include *Ppargc1a*, *Tbk1*, *Acaca*, *Fasn*, *Me1*, *Pnpla2*, *Lipe*, *Glut4*, *F4/80*, and *Ccl2*. Data are presented as log_2_ fold-change relative to lean mice. *n* = 8–10, 1-way ANOVA with Tukey’s multiple-comparison test. (**C**) Heatmap of liver genes related to inflammation (Ccl2, Tnfα), TBK1 signaling (Tbk1), fibrosis (Col1a1, Timp1), ER stress (Ddit3), lipid accumulation (Plin1, Srebf1), and metabolic regulation. Data are presented as log_2_ fold-change relative to lean mice. *n* = 8–10*,* 1-way ANOVA with Tukey’s multiple-comparison test. (**D** and **E**) Immunoblots and quantification of pS79 ACC/ACC, pT172 AMPK/AMPK, pS485 AMPK/AMPK, pS172 TBK1/TBK1, and F4/80 in eWAT. *n* = 6–10, 1-way ANOVA with Tukey’s multiple-comparison test. Data are presented as mean ± SEM; *P < 0.05, **P < 0.01, ***P < 0.001, ****P < 0.0001; ^+^*P* < 0.05, ^++^*P* < 0.01, ^+++^*P* < 0.001. Asterisks designate comparison with ND-fed mice, and plus signs designate comparison with HFD-fed mice.

We next investigated the molecular pathways underlying the metabolic benefits of combined TBK1 inhibition and AMPK activation by examining gene expression related to mitochondrial biogenesis, fatty acid metabolism, glucose transport, and inflammation in white adipose tissue and the liver. HFD feeding suppressed *Ppargc1a* expression in iWAT. Treatment with AICAR or amlexanox alone led to moderate increases in *Ppargc1a*, and the combination produced the strongest induction, implying that TBK1 inhibition amplifies AMPK-driven transcriptional activation. *Tbk1* levels were elevated by HFD and suppressed by both treatments, with the greatest reduction observed in the dual treatment group. HFD also increased *Me1*, a lipogenic gene, which was most effectively downregulated by the combination. Expression of the lipolytic gene *Pnpla2* rose with dual treatment, and *Lipe* showed a similar trend, mirroring elevated serum NEFA and glycerol. Moreover, *Glut4* expression, which was reduced by HFD, was restored by dual therapy. Inflammatory markers *F4/80* and *Ccl2* were markedly increased in obese mice and substantially reduced by the combination, indicating a shift toward a more antiinflammatory adipose tissue environment (Figure 11A and Supplemental Figure 8C).

A similar transcriptional response was observed in eWAT. *Ppargc1a* and *Tbk1* mRNA levels were upregulated by either AICAR or amlexanox, with further elevation upon dual treatment. Fatty acid synthesis genes (*Acaca*, *Fasn*, *Me1*) were broadly repressed by combination therapy, and *Glut4* expression increased beyond the effect of single agents. Inflammatory markers were also most suppressed in the dual treatment group, reflecting both improved metabolic activation and reduced inflammation (Figure 11B and Supplemental Figure 8D).

In the liver, *Tbk1* expression rose in response to HFD but was reduced by all treatments, most notably with the combination. Lipid-associated genes (*Plin1*, *Srebf1*) were elevated in obese mice and decreased with dual therapy, reflecting improvements in hepatic lipid handling. Markers of fibrosis (*Col1a1*, *Timp1*) and ER stress (*Ddit3*) were also attenuated, suggesting broader improvements in hepatic health. Inflammatory transcripts (*Ccl2*, *Tnfα*) were sharply increased by HFD and most effectively suppressed by the combined intervention, indicating robust antiinflammatory effects in the liver as well (Figure 11C and Supplemental Figure 9A).

To validate these transcriptional changes at the protein level, we analyzed signaling pathways in eWAT. HFD-fed mice exhibited reduced phosphorylation of AMPK at Thr172 and of ACC at Ser79, together with elevated inhibitory phosphorylation of AMPK at Ser485 and increased pS172 TBK1. AICAR or amlexanox partially corrected these defects, and the combination fully restored AMPK activation and suppressed TBK1 phosphorylation to near-normal levels. F4/80 protein levels, elevated under HFD, were also most reduced in the dual treatment group, mirroring transcript-level changes and confirming reduced tissue inflammation (Figure 11, D and E).

**Figure 12:**
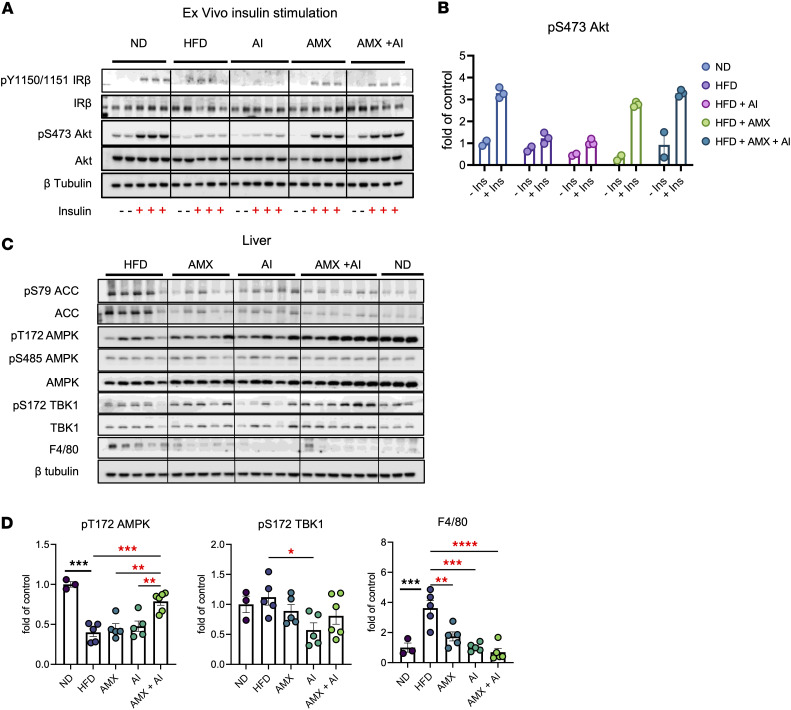
Combined TBK1 inhibition and AMPK activation restores insulin sensitivity in adipose tissue and alleviates hepatic inflammation in obese mice. (**A**) Ex vivo insulin stimulation of eWAT explants from the same study. The eWAT explants were challenged with 100 ng/mL insulin for 30 minutes. Immunoblots of pY1150/1151 IRβ, IRβ, pS473 Akt, Akt, and β tubulin are shown. (**B**) Quantification of pS473 Akt/Akt from the ex vivo insulin stimulation. (**C** and **D**) Immunoblots (**C**) and quantification (**D**) of pS79 ACC/ACC, pT172 AMPK/AMPK, pS485 AMPK/AMPK, pS172 TBK1/TBK1, and F4/80 in liver. *n* = 6–10, 1-way ANOVA with Tukey’s multiple-comparison test. Data are presented as mean ± SEM. *P < 0.05, **P < 0.01, ***P < 0.001, ****P < 0.0001; ^+++^*P* < 0.001.

Given the improvements in glucose tolerance and insulin levels, we next examined insulin signaling in eWAT. Total IRβ and IRS1 protein levels remained stable across conditions, but pS473 Akt was diminished by HFD, indicating impaired insulin response. This was partially improved by AICAR and most effectively by dual therapy (Supplemental Figure 9, B and C). Insulin stimulation of eWAT explants confirmed that HFD blunted insulin-stimulated pY1150/1151 IRβ and pS473 Akt, while amlexanox and, especially, the combination treatment restored both to near-normal levels, showing enhanced insulin responsiveness in adipose tissue (Figure 12, A and B).

In the liver, HFD also reduced pT172 AMPK and pS79 ACC while elevating pS485 AMPK. These changes were partially reversed by single agents and fully corrected by combination treatment. F4/80 protein was also most reduced in the dual therapy group, reflecting diminished hepatic inflammation (Figure 12, C and D). Notably, hepatic insulin signaling, as measured by pS473 Akt, was impaired by an HFD and improved by both treatments, with the combination yielding the most complete restoration (Supplemental Figure 9, D and E), highlighting systemic metabolic benefits.

## Discussion

The signaling pathways that control energy metabolism are subject to numerous feedback loops to ensure homeostasis. This study identifies TBK1 as a nutrient-sensitive checkpoint in adipocytes. Fasting-induced AMPK activation promotes *Tbk1* transcription via a transcriptional pathway involving the PGC1α/NRF1 axis. TBK1 in turn acts to restrain AMPK activity, in the process reducing mitochondrial oxidative capacity and lipid mobilization, thus limiting overall energy expenditure to conserve energy storage. Indeed, both conditional KO of TBK1 in adipocytes and treatment of mice or isolated cells with the specific TBK1/IKKε inhibitor amlexanox restore sensitivity to AMPK activation, either through fasting or a pharmacological activator. Interestingly, although overall levels and activity of TBK1 in adipocytes are elevated in obesity due to increased inflammatory pathways, the further induction of *Tbk1* expression during fasting is repressed in obesity due to the reduced activation of AMPK. These findings build upon previous work implicating TBK1 in obesity (11, 13, 14, 23) and expand its role to acute energy stress, positioning TBK1 as a modulator of catabolic capacity under both nutrient excess and deprivation.

Although previous studies have linked TBK1 to metabolic stress, the precise transcriptional mechanism governing its induction in adipocytes remained elusive. Our data establish a direct regulatory link in which the AMPK/PGC1α/NRF1 signaling axis drives *Tbk1* expression. By utilizing luciferase reporter assays and ChIP-qPCR, we demonstrated that NRF1 binds to the *Tbk1* promoter and is essential for its induction after pharmacological AMPK activation or nutrient deprivation. The attenuation of this response in *Ppargc1a*^AKO^ mice further confirms that PGC1α acts as the requisite coactivator for this transcriptional program. This identifies TBK1 as a primary transcriptional target of the energy-sensing machinery in adipose tissue, rather than a secondary consequence of metabolic flux.

AMPK acts as a central sensor of nutrient deficit, triggering catabolic programs that mobilize energy stores through increased lipid oxidation, mitochondrial remodeling, and inhibition of anabolic processes (24, 25). However, this activation is tightly regulated to prevent excessive energy depletion. Our findings identify TBK1 as a key component of this regulatory checkpoint. It is transcriptionally induced by AMPK via the PGC1α/NRF1 axis and feeds back to suppress AMPK activity. This negative feedback likely evolved as a self-limiting mechanism to preserve a minimal energy reserve during periods of fasting or stress. In lean mice, fasting activates AMPK and induces *Tbk1* to eventually limit catabolic signaling. In obesity, this feedback loop is desensitized; TBK1 expression is already elevated due to chronic inflammation, while AMPK activity is suppressed, blunting the transcriptional response to fasting. This desensitization appears to represent an adaptive mechanism in obesity, allowing adipocytes to preserve energy storage even under nutrient-limited conditions.

TBK1 occupies a unique regulatory position in metabolic control, acting as a brake on energy expenditure under both nutrient excess and nutrient deprivation. Our findings reveal that TBK1 is upregulated in response to nutrient stress. This dual regulation positions TBK1 as a bidirectional suppressor of catabolism. Notably, this distinguishes TBK1 from classical inflammatory kinases, which primarily respond to immune signals (26, 27). Instead, TBK1 integrates nutrient and inflammatory cues to tune the intensity of catabolic programs (4), making it a rare example of a kinase that restrains energy expenditure across divergent metabolic states. Importantly, while TBK1 loss enhanced local adipocyte AMPK signaling and lipolysis, it did not alter systemic β-hydroxybutyrate levels (data not shown), suggesting its primary role is the regulation of local cellular energy sensing within the adipose organ rather than a systemic shift in whole-body ketogenic flux.

In this study, we found that therapeutic potentiation between TBK1 inhibition and AMPK activation offers a strategy to overcome the metabolic inflexibility that characterizes obesity (28). AMPK activators such as AICAR stimulate catabolic pathways (29), whereas obesity impairs AMPK responsiveness in adipose tissue. Elevated TBK1 activity, along with increased expression of its paralog IKKε, further contributes to resistance against energy expenditure–increasing agents such as β-adrenergic agonists (23).

The immediate response to fasting (0–12 hours) is dominated by the rapid, posttranslational activation of catabolic pathways like AMPK to mobilize fuel, whereas our findings suggest that TBK1 functions as a delayed, adaptive checkpoint. The transcriptional induction of *Tbk1* observed at 24 and 48 hours indicates that this pathway is not an immediate fasting sensor, but rather an energy-conservation mechanism that evolves during more significant nutrient scarcity to prevent runaway energy depletion. This compensatory loop appears disrupted in obesity, where chronic inflammation maintains high basal TBK1, effectively locking the “brake” in the on position and contributing to metabolic stagnation.

In addition, our data shows that pharmacological TBK1 inhibition restores sensitivity to AMPK signaling, even in the obese state, enhancing downstream effects on lipid mobilization and insulin responsiveness. When combined, amlexanox and AICAR produced additive improvements in weight loss, glucose tolerance, and adipose inflammation, independent of food intake. We recognize that amlexanox also blocks IKKε activity, and it remains possible that IKKε controls signaling events beyond AMPK to control metabolism. Moreover, we also recognize that both compounds may affect other tissues that influence energy metabolism.

This strategy may also have utility in combination with anorexigenic agents such as GLP-1 receptor agonists, which promote food restriction but are limited by compensatory reductions in basal energy expenditure (30, 31). This metabolic adaptation persists well beyond the active treatment period and contributes to weight regain in most patients (32). By releasing the TBK1-mediated brake on energy expenditure, cotreatment with TBK1 inhibitors may prevent or reverse this adaptive suppression of catabolism, enhancing the durability of weight loss. In addition to GLP-1–based therapies, TBK1 inhibition may enhance the efficacy of energy expenditure–increasing agents, such as AMPK activators and β-adrenergic agonists (33–35). As multiple clinical therapies increasingly target nutrient-sensing pathways to treat obesity, the addition of TBK1 inhibitors may offer a complementary strategy to sustain energy expenditure, restore metabolic flexibility, and improve long-term treatment outcomes.

In summary, TBK1 is a transcriptional target of the AMPK/PGC1α/NRF1 axis and functions as a nutrient- and inflammation-responsive regulator of adipocyte metabolism. Although this feedback loop likely evolved to protect energy reserves during stress, its chronic activation in obesity becomes maladaptive. By targeting this node, TBK1 inhibition restores responsiveness to AMPK signaling, enabling fat oxidation and overcoming metabolic rigidity.

## Methods

### Sex as a biological variable.

Our study examined male mice because male animals exhibited less variability in phenotype.

### Animal studies.

C57BL/6J WT mice were purchased from The Jackson Laboratory, adipocyte-specific AMPKα1/α2-KO (*Prkaa1/2^AKO^*), TBK1-KO (*Tbk1^AKO^*), and PGC1α-KO (*Ppargc1a^AKO^*) mice were generated in our laboratory (UCSD), as previously described (11, 36). Male mice were housed on a 12-hour light/12-hour dark cycle with free access to food and water in accordance with the UCSD IACUC protocol. Animals were maintained on either standard chow or an HFD (60% kcal from fat; D12492, Research Diets) for the indicated durations. For fasting studies, mice were singly housed with continuous access to water for 24–72 hours. Acute AMPK activation was achieved by i.p. injection of 500 mg/kg AICAR (Sigma-Aldrich), and animals were euthanized 0.5–6 hours later. Amlexanox (Sigma-Aldrich) was administered at 25 mg/kg by daily oral gavage for the specified treatment period. For combination therapy, mice received AICAR (100 mg/kg i.p., every other day) together with amlexanox (25 mg/kg by oral gavage, daily) for 21 days. Pair-feeding controls were established by recording daily food intake in amlexanox-treated cages and providing the same total amount per mouse to control cages the following day.

### Cell culture.

We purchased 3T3-L1 preadipocytes (CL-173) and HEK293T cells (CRL-3216) from ATCC. Stromal vascular fraction–derived preadipocytes were isolated from mouse iWAT and cultured as previously described (37). Briefly, cells isolated from mouse iWAT were maintained in DMEM or DMEM/F12 supplemented with 10% newborn calf serum (Gibco) or FBS (Corning) and 1% penicillin–streptomycin (Gibco). Differentiation was induced 2 days after reaching confluence, and fully differentiated adipocytes (day 8–10) were used for experiments. For drug treatments, cells were incubated with 500 μM AICAR, 10 μM PF-739, 10 μM Compound C, or 50 μM amlexanox for the indicated times.

### siRNA transfection.

Differentiated 3T3-L1 adipocytes were transfected with siRNA targeting AMPK α1/α2, NRF1, or nontargeting control using Lipofectamine RNAiMAX (Invitrogen) in Opti-MEM medium (Gibco), following the manufacturer’s instructions. Medium was replaced after 24 hours, and cells were assayed 72 hours after transfection.

### Quantitative PCR.

Total RNA was extracted from cells using the PureLink RNA kit (Invitrogen) or from tissues using TRIzol Reagent (Invitrogen). RNA (500 ng–1 μg) was reverse-transcribed using HiScript II Reverse Transcriptase (Vazyme). qPCR was performed with Taq Pro universal SYBR (Vazyme) on a QuantStudio 6 Flex Real-Time PCR System (Applied Biosystems). Relative gene expression was calculated using the standard curve method and normalized to ARBP as the reference gene. Primer sequences are provided in Supplemental Table 1.

### mRNA stability.

Adipocytes were treated with 10 μM PF-739 (MedChem Express) or vehicle for 6 hours, followed by 5 μg/mL actinomycin D to block transcription. Cells were harvested at 0, 30, 60, 120, and 240 minutes after treatment for RNA extraction using TRIzol (Invitrogen). *Tbk1* mRNA was quantified by RT-qPCR, normalized to *Arbp*, and decay rates calculated by linear regression.

### Western blotting.

Cells and tissues were lysed in lysis buffer (50 mM Tris pH 7.4, 150 mM NaCl, 1.5 mM MgCl_2_, 10% glycerol, 0.2 mM EGTA, 0.2 mM EDTA, 1% Triton X-100) containing protease and phosphatase inhibitors (Thermo Fisher Scientific). Protein concentration was measured by BCA (Thermo Fisher Scientific). Equal protein amounts were separated by SDS-PAGE (Invitrogen) and transferred to PVDF membranes (MilliporeSigma). Membranes were blocked in 5% nonfat dry milk in TBST and probed with primary antibodies at 4°C overnight. HRP-conjugated secondary antibodies (Thermo Fisher Scientific) were used for detection via enhanced chemiluminescence (Pierce) and imaged with a ChemiDoc system (Li-Cor). Each target shown was probed on an independently loaded gel, unless otherwise noted.

### Single-nucleus RNA-seq of mature adipocytes.

Single-nucleus RNA-seq (snRNA-seq) data of mouse adipocytes were obtained from NCBI’s Gene Expression Omnibus (GSE176171) (38), and a processed Seurat (39) object of adipocyte snRNA-seq data was downloaded (https://gitlab.com/rosen-lab/white-adipose-atlas). Data quality and consistency were assessed using UMAP dimensionality reduction. Gene expression was visualized using the Seurat and ggplot2 packages in R with log-normalized expression. Statistical significance was determined by the Wilcoxon rank-sum test with FDR adjustment.

### Luciferase reporter assay.

The mouse TBK1 promoter region containing the predicted NRF1 binding site was amplified from mouse genomic DNA by PCR using the primers listed in Supplemental Table 1. PCR amplification was performed using PrimeSTAR Max DNA Polymerase (Takara, R045A). The pGL3-Basic vector (Promega, E1751) was double digested with KpnI and XhoI and purified prior to cloning. The amplified promoter fragment was inserted into the linearized pGL3-Basic vector by homologous recombination according to the manufacturer’s instructions (Takara, 638955). The recombination products were transformed into *E*. *coli* DH5α-competent cells, and positive clones were selected on Luria-Bertani agar plates containing ampicillin. Correct insertion and sequence integrity were confirmed by Sanger sequencing. Next, HEK293T cells were seeded in 24-well plates and transfected at 80% confluence using Lipofectamine 3000 (Thermo Fisher Scientific). Cells were cotransfected with Tbk–luciferase reporter plasmid, *Renilla* luciferase control plasmid, and expression plasmids for WT AMPK, kinase-dead AMPK, or PGC1α. At 48 hours after transfection, cells were lysed and luciferase activity was measured using the Dual-Luciferase Reporter assay system on a GloMax plate reader (Promega). Firefly luciferase activity was normalized to *Renilla* activity. Data are expressed as fold-change in relative luciferase activity compared with the control group.

### Body composition.

Fat and lean mass were measured in conscious, unrestrained mice using EchoMRI (Echo Medical Systems) according to the manufacturer’s instructions. Each scan lasted approximately 2 minutes, and animals were returned immediately to their home cages.

### Glucose tolerance test.

Glucose tolerance was assessed after a 16-hour fast. Baseline blood glucose was measured from tail vein blood before i.p. glucose injection (1.2 g/kg body weight in sterile saline). Subsequent glucose levels were measured at 30, 60, 90, and 120 minutes after injection using an Accu-Chek handheld glucometer.

### Blood and plasma analyses.

Blood glucose was measured from tail vein blood in either fasted or non-fasted conditions using a glucometer (Accu-Chek). Plasma NEFA levels were measured with the NEFA-HR (2) colorimetric assay kit (Fuji Film Wako) following the manufacturer’s instructions. Plasma glycerol concentrations were determined using the Free Glycerol reagent (Sigma-Aldrich) and calculated against a standard curve prepared in assay buffer. Plasma insulin was quantified using the Ultra-Sensitive Mouse Insulin ELISA kit (Crystal Chem). All plasma assays were performed in duplicate, and standards and controls were included on each plate.

### Liver triglycerides.

Liver triglyceride content was measured using a modified Folch extraction. Briefly, 30–80 mg of frozen liver was homogenized in buffer (50 mM Tris-HCl pH 8.0, 5 mM EDTA, 30 mM mannitol) with protease inhibitors, followed by addition of chloroform/methanol (2:1, v/v). The organic phase was collected, dried overnight, and resuspended in a butanol/Triton X-114/methanol mixture (9:5:1). Triglycerides were quantified using triglyceride reagent (Sigma-Aldrich) according to the manufacturer’s instructions.

### Ex vivo insulin stimulation in eWAT.

eWAT pads were dissected from euthanized mice, rinsed in ice-cold PBS, and minced into 5–10 mg fragments. The eWAT explants were preincubated in DMEM (Gibco) supplemented with 1% penicillin-streptomycin at 37°C, 5% CO_2_ for 1 hour. Next, 100 ng/mL insulin (Sigma-Aldrich) was added for 30 minutes, after which the explants were rapidly washed in ice-cold PBS and snap-frozen for Western blot analysis.

### Histology.

For H&E staining, tissues were fixed in 4% paraformaldehyde (Sigma-Aldrich) for 48 hours, transferred to 70% ethanol, and embedded in paraffin. The H&E staining and imaging of the tissues were performed by the UCSD Tissue Technology Shared Resource. Adipocyte size was quantified from 10 fields per mouse from at least 3 mice using Adiposoft (ImageJ, NIH) with manual correction for artifacts.

### CLS detection.

The F4/80 staining and imaging of iWAT and eWAT were performed by the UCSD Tissue Technology Shared Resource. Images from the microscope at a 40× original magnification were converted to grayscale, and a binary threshold was applied to segment cell membranes and contents. Each adipocyte was identified as a connected component within the binary image. The F4/80 stain was identified by locating regions where the red-to-blue intensity ratio in the original color image exceeded 1.05. A cell was classified as having a CLS if the F4/80-stained area surrounding its membrane constituted more than 30% of the total membrane area. The results were corrected manually for artifacts.

### Antibodies.

The following antibodies were purchased from Cell Signaling Technology: pT172 AMPKα (catalog 2531), pS485 AMPKα (catalog 4184), AMPKα (catalog 5831), pS79 ACC (catalog 3661), ACC (catalog 3676), pS555 ULK1 (catalog 5869), ULK1 (catalog 18938), pS172 TBK1 (catalog 5483), TBK1(catalog 3504), pS792 raptor (catalog 2083), raptor (catalog 2282), pY1150/1151 IRβ (catalog 3024), IRS1 (catalog 3407), pS473 Akt (catalog 4060), Akt (catalog 4691), NRF1 (catalog 46743), F4/80 (catalog 70076), VDAC (catalog 4661), histone H3 (catalog 9715), HSP90 (catalog 4877), and β-tubulin (catalog 2128). IRβ antibody (catalog 81465) was purchased from Santa Cruz Biotechnology. RalA antibody was purchased from BD Biosciences (catalog 610221). OXPHOS antibody (catalog 45-8099) was purchased from Thermo Fisher Scientific.

### Statistics.

Data are presented as mean ± SEM. Statistical significance was determined using 2-tailed Student’s *t* test or 1- or 2-way ANOVA with Tukey’s post hoc test as appropriate with GraphPad Prism 9. A *P* value less than 0.05 was considered statistically significant.

### Study approval.

All animal use was approved by the IACUC at UCSD (San Diego, California, USA).

### Data availability.

All data are available in the main text or in the Supporting Data Values file.

## Author contributions

ARS supervised and conceptualized the project and acquired funding. CW conceptualized and investigated the project. CW, YS, HK, HF, XF, SS, SZ, LC, AH, HC, SB, and PL performed the experiments. PW, MA, SMR, PZ, JV, and JYH supervised the project. All authors reviewed and edited the manuscript.

## Conflict of interest

ARS is a named inventor on a patent (patent 10214536) pertaining to the use of amlexanox and its analogs for the treatment of metabolic diseases. ARS is a cofounder of Elgia Therapeutics.

## Funding support

This work is the result of NIH funding, in whole or in part, and is subject to the NIH Public Access Policy. Through acceptance of this federal funding, the NIH has been given a right to make the work publicly available in PubMed Central.

- NIH/NIDDK: P30DK063491, R01DK122804, R01DK124496, R01DK125820, and R01DK128796 (to ARS).
- Cancer Institute Cancer Center Support grant: P30CA23100 (to UCSD Tissue Technology Shared Resource).
- National Research Foundation of Korea (NRF): 2021R1C1C2010446 (to JYH; funded by the Korean government [Ministry of Science and ICT]).
- Global-LAMP program of NRF: RS-2024-00441954 (to JYH; funded by the Ministry of Education).

## Supplementary Material

Supplemental data

Unedited blot and gel images

Supporting data values

## Version 1 05/08/2026

Electronic publication
