# Supplementary material for: A nutrient-responsive AMPK/TBK1 circuit restricts adipocyte catabolism
Source: JCI Insight. 2026 May 8;11(9):e200168. doi: 10.1172/jci.insight.200168 (PMC13232487; doi:10.1172/jci.insight.200168)
Supplement: Supplemental data [file jciinsight-11-200168-s258.pdf]

Supplemental Material:

Churaibhon Wisessaowapak[1], Yuliya Skorobogatko[1], Hyeonhui Kim[1], Xue Feng[1], Seunghwan Son[1], Haipeng Fu[1], Sitao Zhang[2], Pichaya Lertvilai[3], Lina Chang[1], Annie Hoang[1], Hetty Chen[1], Sarah Bedsted[1], Joseph M. Valentine[1], Jin Young Huh[1, 4], Peng Zhao[1, 5], Shannon M. Reilly[1, 6], Piyajit Watcharasit[7], Maryam Ahmadian[1], Alan R. Saltiel [1, 8]

[1] Division of Endocrinology and Metabolism, Department of Medicine and Pharmacology,

University of California San Diego, San Diego, CA, USA.

[2] Department of Cellular and Molecular Medicine, University of California San Diego, San Diego, CA, USA

[3] Marine Physical Lab, Scripps Institution of Oceanography, University of California San Diego, CA, USA

[4] Center for Nano Materials, Department of Life Science, Sogang University, Seoul, Republic of Korea

[5] Department of Biochemistry and Structural Biology,

University of Texas Health Science Center at San Antonio, San Antonio, TX, USA.

[6] Weill Center for Metabolic Health, Division of Endocrinology, Diabetes and Metabolism,

Department of Medicine, Weill Cornell Medicine, New York, NY, USA.

[7] Laboratory of Pharmacology, Chulabhorn Research Institute, Bangkok, Thailand.

[8] Correspondence: asaltiel@ucsd.edu

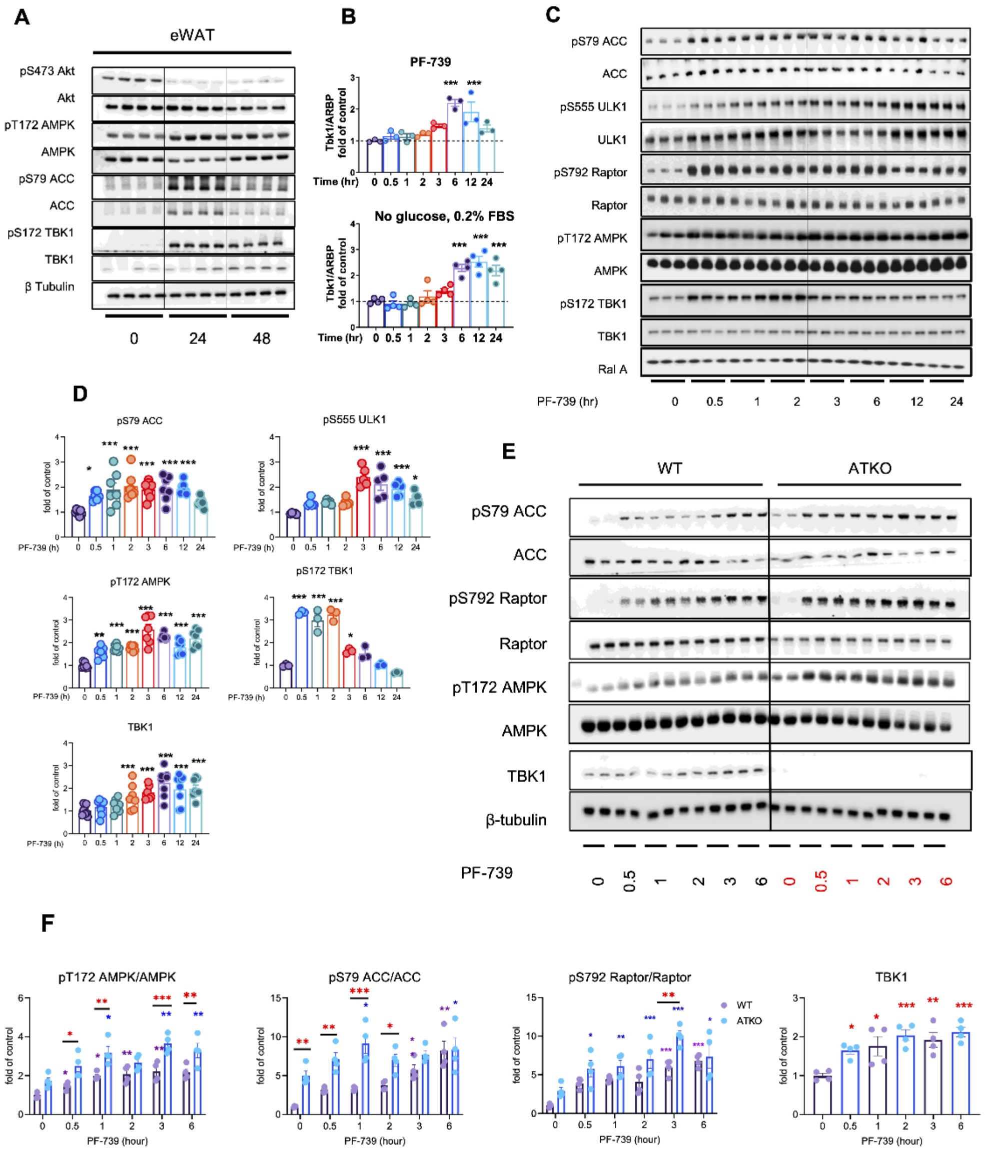

Supplemental Figure 1. **AMPK-TBK1 feedback loop regulates adipocyte catabolism during nutrient stress.**

(A) Immunoblot analysis of eWAT from ND mice fasted for 0, 24, or 48 h, probed for pS473 Akt, Akt, pT172 AMPK, AMPK, pS79 ACC, ACC, pS172 TBK1, TBK1, and β-tubulin.

(B-C) qPCR analysis of *Tbk1* mRNA in differentiated 3T3-L1 adipocytes treated with 10 µM PF-739 for the indicated times (A) or subjected to glucose and serum deprivation (0.2% FBS, no

glucose) for the indicated times (B). *n = 3-4*, one-way ANOVA with Tukey's multiple-comparison test.

(C) Immunoblot analysis of 3T3-L1 adipocytes treated with 10 μM PF-739 for the indicated times, probed for pS79 ACC, ACC, pS555 ULK1, ULK1, pS792 Raptor, Raptor, pT172 AMPK, AMPK, pS172 TBK1, TBK1, and RalA.

(D) Quantification of immunoblots in (C) for the indicated targets. *n = 3-7*, one-way ANOVA with Tukey's multiple-comparison test.

(E–F) Immunoblot analysis (E) and quantification (F) of primary preadipocytes differentiated *in vitro* (PPDIVs) from WT and *Tbk1*^{AKO} (ATKO) mice treated with 10 μM PF-739 for the indicated times. Blots were probed for pS79 ACC, pS792 Raptor, pT172 AMPK, and their respective total proteins to demonstrate the functional effect of TBK1 loss on diverse AMPK substrates. *n = 4*, two-way ANOVA with Tukey's multiple-comparison test.

Data are presented as mean ± SEM; each dot represents a biological replicate. *p < 0.05, **p < 0.01, ***p < 0.001. Each target shown was probed on an independently loaded gel unless otherwise noted.

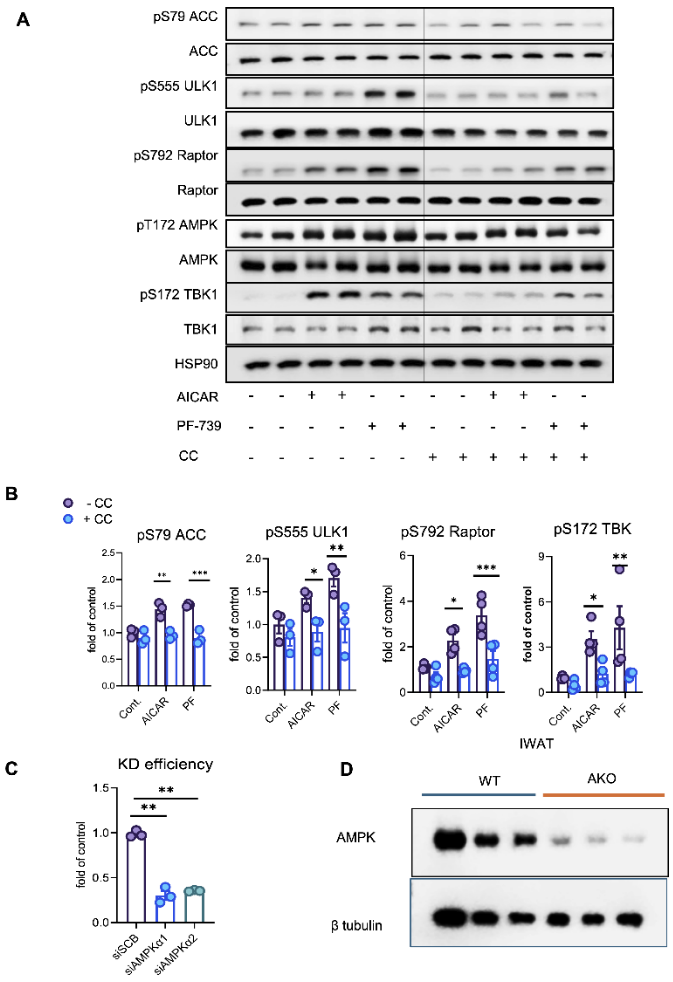

Supplemental Figure 2. Inhibition of AMPK suppresses TBK1 induction.

(A) Immunoblot analysis of differentiated 3T3-L1 adipocytes pretreated with 10 μM Compound C (CC) for 30 min, followed by stimulation with 500 μM AICAR or 10 μM PF-739 for 6 h. Blots were probed for pS79 ACC, ACC, pS555 ULK1, ULK1, pS792 Raptor, Raptor, pT172 AMPK, AMPK, pS172 TBK1, TBK1, and HSP90.

(B) Quantification of immunoblots in (A) for pS79 ACC/ACC, pS555 ULK1/ULK1, pS792 Raptor/Raptor, and pS172 TBK1/TBK1. *n = 3*, two-way ANOVA with Tukey's multiple-comparison test.

(C) qPCR analysis of *Prkaa1/2* (AMPKα1/α2) knockdown efficiency in differentiated 3T3-L1 adipocytes transfected with control siRNA (siSCB) or AMPKα1/α2 siRNA. $n = 3$, one-way ANOVA with Tukey's multiple-comparison test.

(D) Immunoblot analysis confirming the depletion of AMPK protein in inguinal white adipose tissue (iWAT) from adipocyte-specific *Prkaa1/2*^{AKO} (AAKO) mice compared to WT controls.

Data are presented as mean ± SEM; each dot represents a biological replicate. *p < 0.05, **p < 0.01, ***p < 0.001. Each target shown was probed on an independently loaded gel unless otherwise noted.

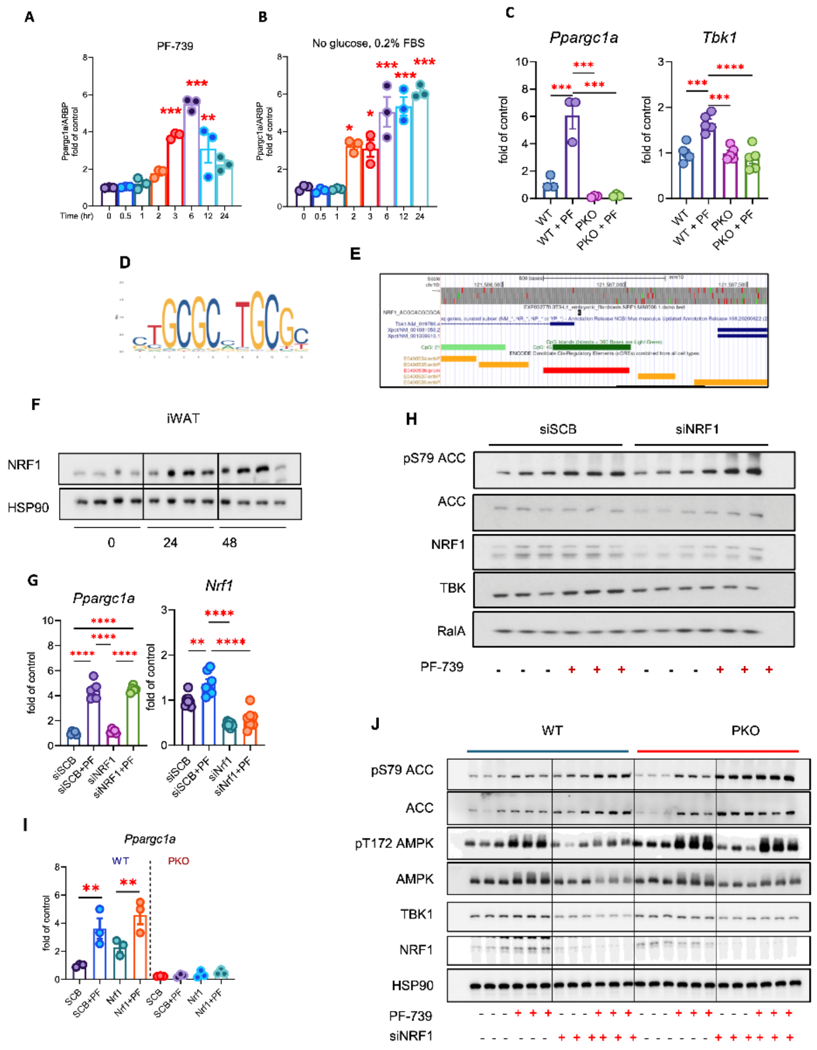

Supplemental Figure 3. The AMPK–PGC1α–NRF1 axis regulates *Tbk1* transcription.

(A–B) qPCR analysis of *Ppargc1a* mRNA in differentiated 3T3-L1 adipocytes treated with 10 μM PF-739 (A) or cultured in nutrient-deprived medium (0.2% FBS, no glucose) for the indicated times (B). *n = 3*, one-way ANOVA with Tukey's multiple-comparison test.

(C) qPCR analysis of *Ppargc1a* and *Tbk1* mRNA in mature adipocytes isolated from WT and *Ppargc1a*^{AKO} (PKO) mice treated with 10 μM PF-739 for 6 h. *n = 3-5*, one-way ANOVA with Tukey's multiple-comparison test.

(D) Predicted binding motif of NRF1 on the Murine *Tbk1* promoter.

(E) UCSC Genome Browser view of the Murine *Tbk1* locus on chromosome 10, showing RefSeq annotations, CpG islands, ENCODE candidate cis-regulatory elements (cCREs), and predicted NRF1 binding motifs near the transcription start site.

(F) Immunoblot analysis of NRF1 protein in iWAT from lean mice fasted for 24 or 48 h, with HSP90 as a loading control.

(G) qPCR analysis of *Ppargc1a* and *Nrf1* mRNA in 3T3-L1 adipocytes transfected with scramble or Nrf1 siRNA, with or without 10 μM PF-739 treatment for 6 h. $n = 3\text{-}5$, one-way ANOVA with Tukey's multiple-comparison test.

(H) Immunoblot analysis of 3T3-L1 adipocytes transfected with scramble or Nrf1 siRNA and treated with 10 μM PF-739, probed for pS79 ACC, NRF1, TBK1, and RalA.

(I) qPCR analysis of *Ppargc1a* mRNA in mature adipocytes from WT and *Ppargc1a* ^{AKO} (PKO) mice to validate genetic loss of the transcript. $n = 3$, one-way ANOVA with Tukey's multiple-comparison test.

(J) Immunoblot analysis of mature adipocytes from WT and *Ppargc1a* ^{AKO} mice transfected with NRF1 siRNA followed by 10 μM PF-739 treatment, probed for pS79 ACC, pT172 AMPK, TBK1, NRF1, and HSP90.

Data are presented as mean ± SEM; each dot represents a biological replicate. **p < 0.01, ***p < 0.001, ****p < 0.0001. Each target shown was probed on an independently loaded gel unless otherwise noted.

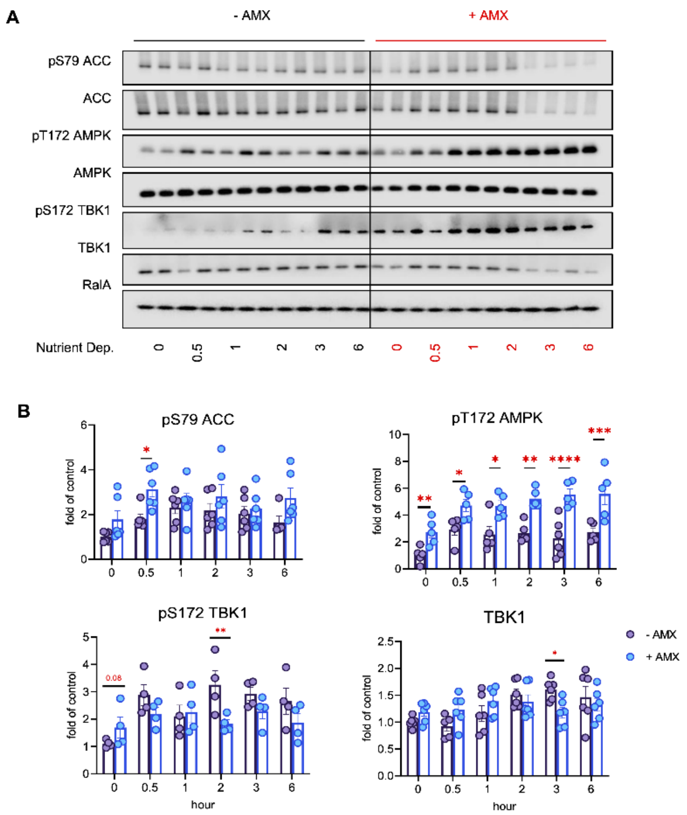

Supplemental Figure 4. TBK1 inhibition enhances AMPK activity in adipocytes during nutrient deprivation

(A) Immunoblots of differentiated 3T3-L1 adipocytes cultured in nutrient-deprived medium (no glucose, 0.2% FBS) for 0.5–6 h with or without 50 μM amlexanox (AMX). Blots for pS79 ACC, ACC, pT172 AMPK, AMPK, pS172 TBK1, TBK1, and RalA are shown.

(B) Quantification of pS79 ACC/ACC, pT172 AMPK/AMPK, and pS172 TBK1/TBK1 from (A), expressed as fold change relative to control. n = *4-6*, one-way ANOVA with Tukey's multiple-comparison test.

Data are presented as mean ± SEM; each dot represents a biological replicate. *p < 0.05, **p < 0.01, ***p < 0.001. Each target shown was probed on an independently loaded gel unless otherwise noted.

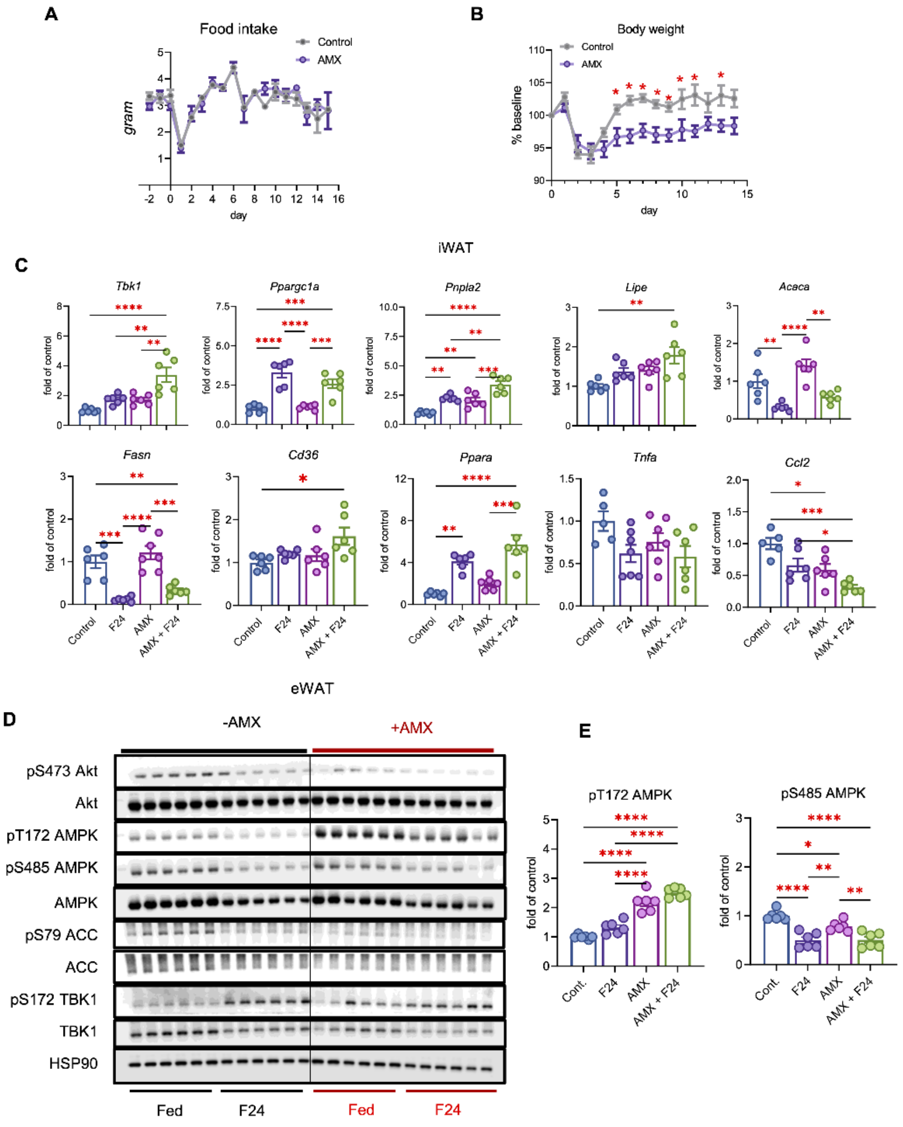

Supplemental Figure 5. Amlexanox enhances fasting-induced AMPK–TBK1 signaling and metabolic gene remodeling

(A–B) Body weight (A) and food intake (B) in lean mice treated with 25 mg/kg amlexanox (AMX) daily by oral gavage for 2 weeks. Pair-fed controls matched the reduced food intake of AMX-treated mice.

(C) qPCR of *Pnpla2, Lipe, Ppara, Ppargc1a, Fasn, Acaca, Tnfa*, and *Ccl2* in iWAT from lean mice treated with 25 mg/kg AMX then subjected to fasting for 24 h. *n = 4-6*, one-way ANOVA with Tukey's multiple-comparison test.

(D) Immunoblot analysis of iWAT from lean mice treated with vehicle or AMX for 2 weeks and subjected to 24 h fasting, probed for pS473 Akt, Akt, pT172 AMPK, pS485 AMPK, AMPK, pS79 ACC, ACC, pS172 TBK1, TBK1, and HSP90.

(E) Quantification of pT172 AMPK/AMPK, and pS485 AMPK/AMPK from (D). $n = 6$, one-way ANOVA with Tukey's multiple-comparison test.

Data are presented as mean ± SEM; each dot represents a biological replicate. *$p < 0.05$, **$p < 0.01$, ***$p < 0.001$. Each target shown was probed on an independently loaded gel unless otherwise noted.

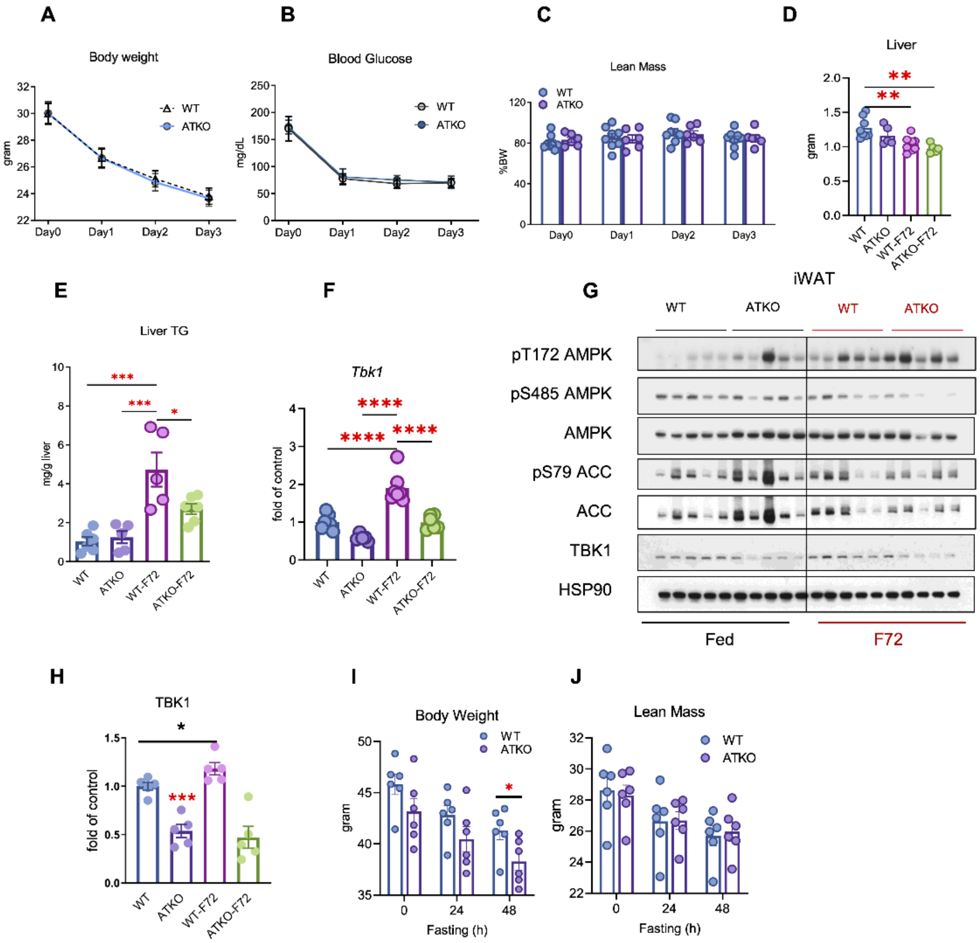

Supplemental Figure 6. TBK1 inhibition enhances AMPK activity and promotes adipose lipolysis during prolonged fasting.

(A–B) Body weight (A) and blood glucose (B) in WT and ATKO mice during 0–3 days of fasting. n= 6-8.

(C) Lean mass from WT and ATKO mice was measured by EchoMRI. WT; wild-type. ATKO; *Tbk1*^{AKO} mice. *n* = *6-8*, two-way ANOVA with Tukey's multiple-comparison test.

(D–E) Liver weight (D) and hepatic triglyceride content (E) in WT and ATKO mice after 72 h fasting. WT; wild-type. ATKO; *Tbk1*^{AKO} mice. *n* = *5-6*, one-way ANOVA with Tukey's multiple-comparison test.

(F) *Tbk1* mRNA expression in iWAT from WT and ATKO mice after 72 h fasting. WT; wild-type. ATKO; *Tbk1*^{AKO} mice. *n* = *6*, one-way ANOVA with Tukey's multiple-comparison test.

(G–H) Immunoblot analysis (G) and quantification (H) of TBK1 protein in iWAT from WT and ATKO mice after 72 h fasting. WT; wild-type. ATKO; *Tbk1*^{AKO} mice.

(I–J) Body weight (I) and lean mass (J) in HFD-fed WT and ATKO mice after 48 h fasting. WT; wild-type. ATKO; *Tbk1*^{AKO} mice. $n = 6$, two-way ANOVA with Tukey's multiple-comparison test. Data are presented as mean ± SEM; each dot represents a biological replicate. *p < 0.05, **p < 0.01, ***p < 0.001. Each target shown was probed on an independently loaded gel unless otherwise noted.

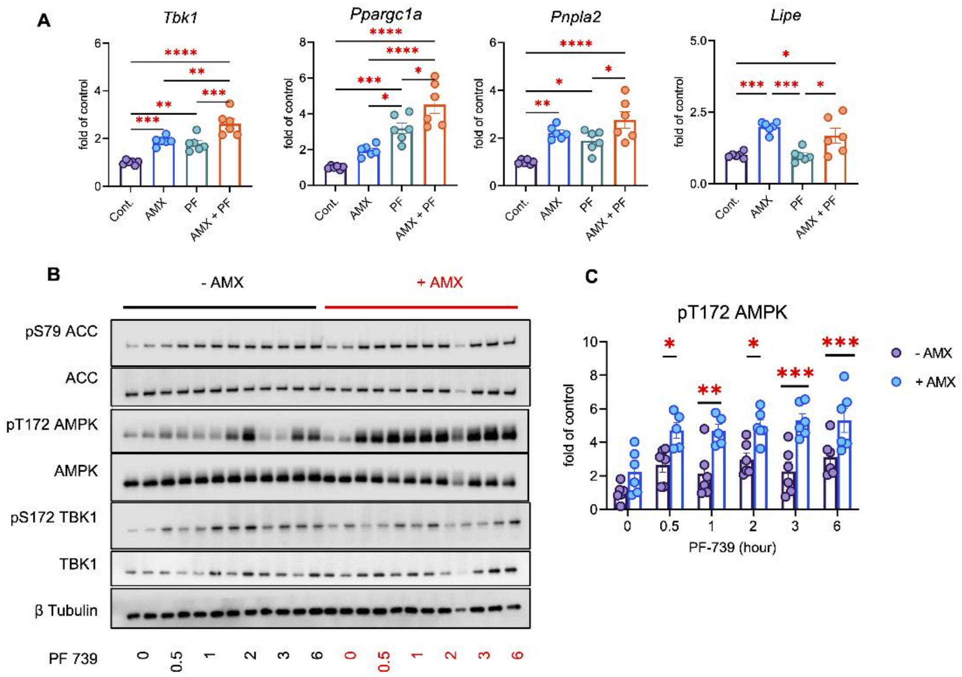

Supplemental Figure 7. Effects of combined TBK1 inhibition and AMPK activation on gene expression and AMPK signaling in adipocytes

(A) qPCR of Tbk1, Ppargc1a, Pnpla2, and Lipe in differentiated 3T3-L1 adipocytes treated with vehicle, 50 μM amlexanox (AMX), 10 μM PF-739, or both for 6 h. *n = 6*, one-way ANOVA with Tukey's multiple-comparison test.

(B) Immunoblots of pS79 ACC, ACC, pT172 AMPK, AMPK, pS172 TBK1, TBK1, and β-tubulin in adipocytes treated with 10 μM PF-739 for the indicated times with or without 50 μM AMX.

(C) Quantification of pT172 AMPK/AMPK from (B), expressed as fold of control. *n = 6*, two-way ANOVA with Tukey's multiple-comparison test.

Data are presented as mean ± SEM. *p < 0.05, **p < 0.01, ***p < 0.001, ****p < 0.0001. Each target shown was probed on an independently loaded gel unless otherwise noted.

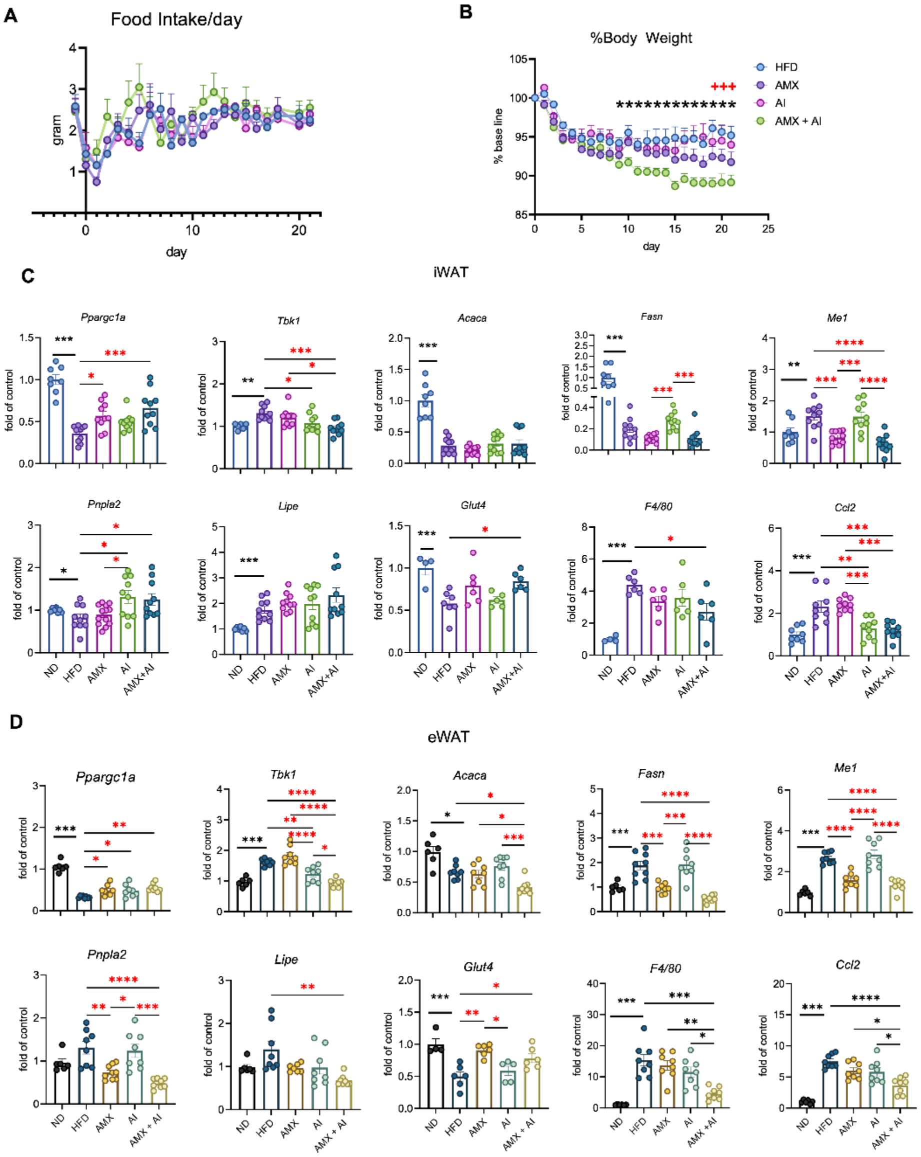

Supplemental Figure 8. Impact of TBK1 inhibition and AMPK activation on energy balance and adipose tissue gene expression in obese mice.

(A) Daily food intake during treatment. All groups were pair-fed to match the reduced intake of AMX-treated mice. n=8-10.

(B) % weight change from baseline in HFD-fed mice treated with 25 mg/kg amlexanox (AMX) daily, 100 mg/kg AICAR (AI) i.p. every other day, or both for 21 days. *Compared to HFD-fed

mice, [+]Compared to AMX-treated mice. *n = 8-10*, one-way ANOVA with Tukey's multiple-comparison test.

(C–D) qPCR of *Ppargc1a, Tbk1, Acaca, Fasn, Me1, Pnpla2, Lipe, Glut4, F4/80,* and *Ccl2* in iWAT (C) and eWAT (D) from ND-fed controls and treated HFD-fed mice. Expression is shown as fold of ND controls. *n = 8-10*, one-way ANOVA with Tukey's multiple-comparison test.

Data are presented as mean ± SEM. $^{*}p < 0.05$, $^{**}p < 0.01$, $^{***}p < 0.001$, $^{****}p < 0.0001$.

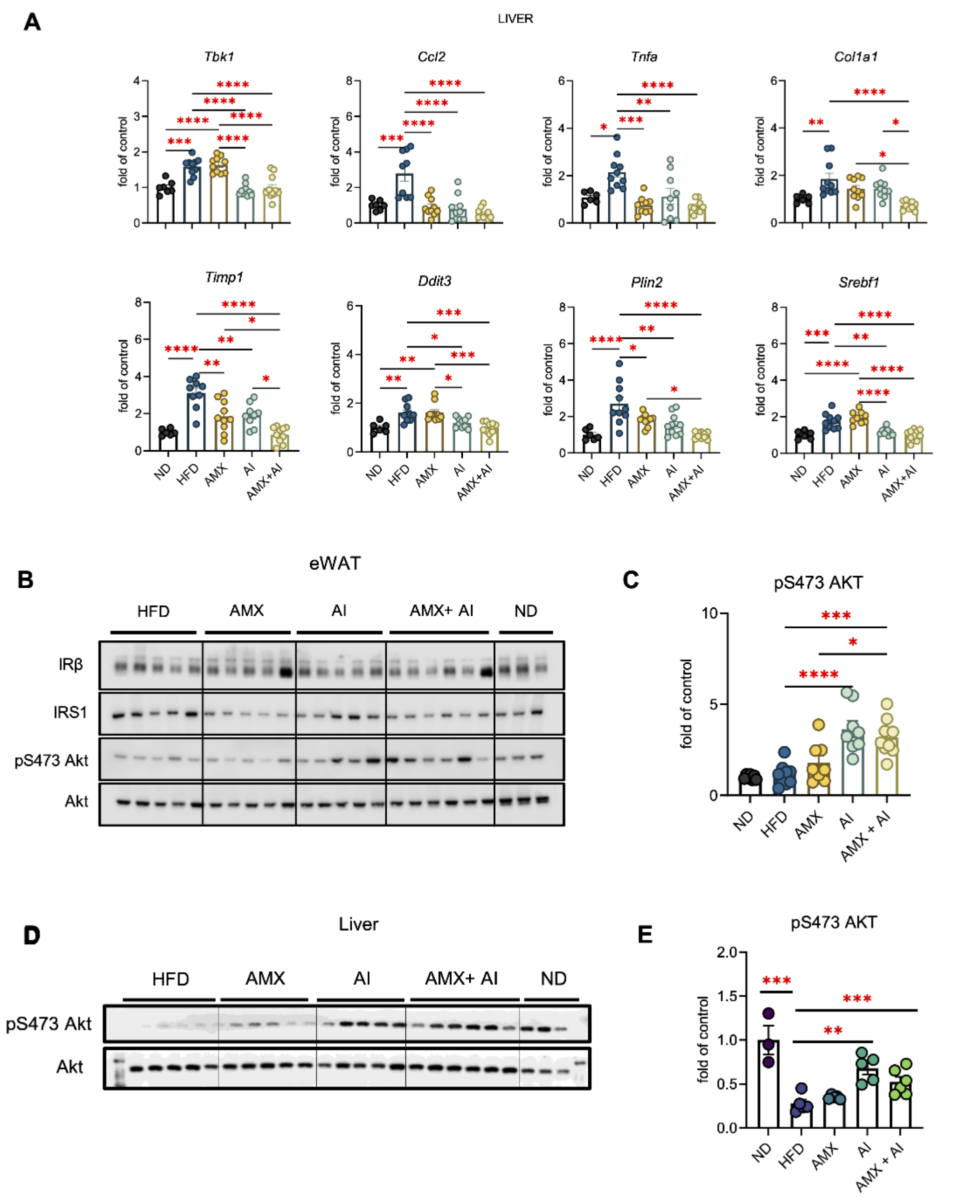

Supplemental Figure 9. TBK1 inhibition and AMPK activation modulate hepatic gene expression and insulin signaling in adipose tissue and liver

(A) qPCR of *Tbk1, Ccl2, Tnfα, Col1a1, Timp1, Ddit3, Plin1, and Srebf1* in liver from ND-fed or HFD-fed mice treated with 25 mg/kg amlexanox (AMX) daily, 100 mg/kg AICAR (AI) i.p. every other day, or both for 21 days. Expression normalized to *Arbp* and shown as fold of ND controls. *Compared to ND-fed mice; +compared to HFD-fed mice. *n = 8-10*, one-way ANOVA with Tukey's multiple-comparison test.

(B–C) Immunoblots of IRβ, IRS1, pS473 Akt, Akt and (C) the quantitation of pS473 Akt/Akt in eWAT, showing reduced Akt phosphorylation in HFD-fed mice, partially restored by AICAR and most strongly by AMX + AICAR. *n = 8-10*, one-way ANOVA with Tukey's multiple-comparison test.

(D–E) Immunoblots (D) and quantification (E) of pS473 Akt/Akt in liver from ND-fed and treated HFD-fed mice. *n = 3-6*, one-way ANOVA with Tukey's multiple-comparison test.

Data are presented as mean ± SEM. *$p < 0.05$, **$p < 0.01$, ***$p < 0.001$.

Supplemental table 1. Primer Sequences

**Gene**	**Forward (5'-3')**	**Reverse (5'-3')**
Acaca	GTTCTGTTGGACAACGCCTTCAC	GGAGTCACAGAAGCAGCCCATT
Arbp	GCTTCGTGTTCACCAAGGAGGA	GTCCTAGACCAGTGTTCTGAGC
Ccl2	GCTACAAGAGGATCACCAGCAG	GTCTGGACCCATTCCTTCTTGG
Col1a1	CCTCAGGGTATTGCTGGACAAC	CAGAAGGACCTTGTTTGCCAGG
Ddit3	GGAGGTCCTGTCCTCAGATGAA	GCTCCTCTGTCAGCCAAGCTAG
F4/80	CGTGTTGTTGGTGGCACTGTGA	CCACATCAGTGTTCCAGGAGAC
Fasn	GGAGGTGGTGATAGCCGGTAT	TGGGTAATCCATAGAGCCCAG
Glut4	GGTGTGGTCAATACGGTCTTCAC	AGCAGAGCCACGGTCATCAAGA
Lipe	GGCTCACAGTTACCATCTCACC	GAGTACCTTGCTGTCCTGTCC
Me1	AGAGCAGTGCTACAAGGTGACC	CCAAGAGCAACTCCAGGGAACA
Nrf1	GGCAACAGTAGCCACATTGGCT	GTCTGGATGGTCATTTCACCGC
Plin1	GAGAAGGTGGTAGAGTTCCTCC	GTGTGTCGAGAAAGAGTGTTGGC
Pnpla2	CAACGCCACTCACATCTACGG	TCACCAGGTTGAAGGAGGGAT
Ppara	ACCACTACGGAGTTCACGCATG	GAATCTTGCAGCTCCGATCACAC
Ppargc1a	CCACTTCAATCCACCCAGAAA	TATGGAGTGACATAGAGTGTGCT
Prkaa1	GGTGTACGGAAGGCAAAATGGC	CAGGATTCTTCCTTCGTACACGC
Prkaa2	CTGAAGCCAGAGAATGTGCTGC	GAGATGACCTCAGGTGCTGCAT
Srebf1	CGACTACATCCGCTTCTTGCAG	CCTCCATAGACACATCTGTGCC
Tbk1	GACAGCATAGAGATCACCAGTT	CAGAGCACCTCCAACCATC
Timp1	TCTTGGTTCCCTGGCGTACTCT	GTGAGTGTCACTCTCCAGTTTGC
Tnfa	ACGGCATGGATCTCAAAGAC	AGATAGCAAATCGGCTGACG
Tbk1 promoter - LUC	TTTCTCTATCGATAGGTACCACT CCCTTAGCTCCTCCCG	GATCGCAGATCTCGAGTCTTACCCC GCGAGTGTCC
