# Supplementary material for: A nutrient-responsive AMPK/TBK1 circuit restricts adipocyte catabolism
Source: JCI Insight. 2026 May 8;11(9):e200168. doi: 10.1172/jci.insight.200168 (PMC13232487; doi:10.1172/jci.insight.200168)

Uncropped blots

A nutrient-responsive AMPK–TBK1 circuit restricts adipocyte catabolism

Figure 1C- iWAT

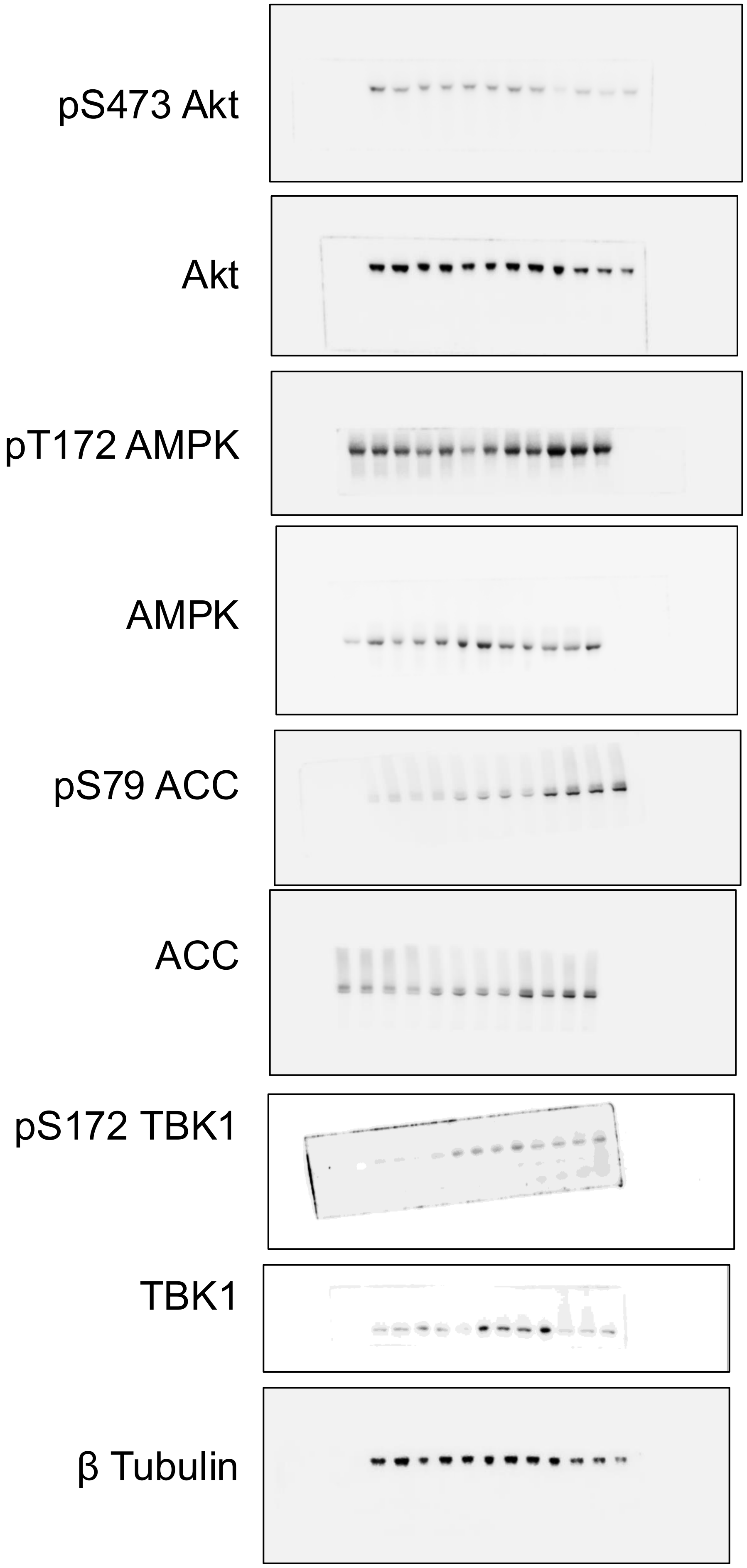

Figure 2B

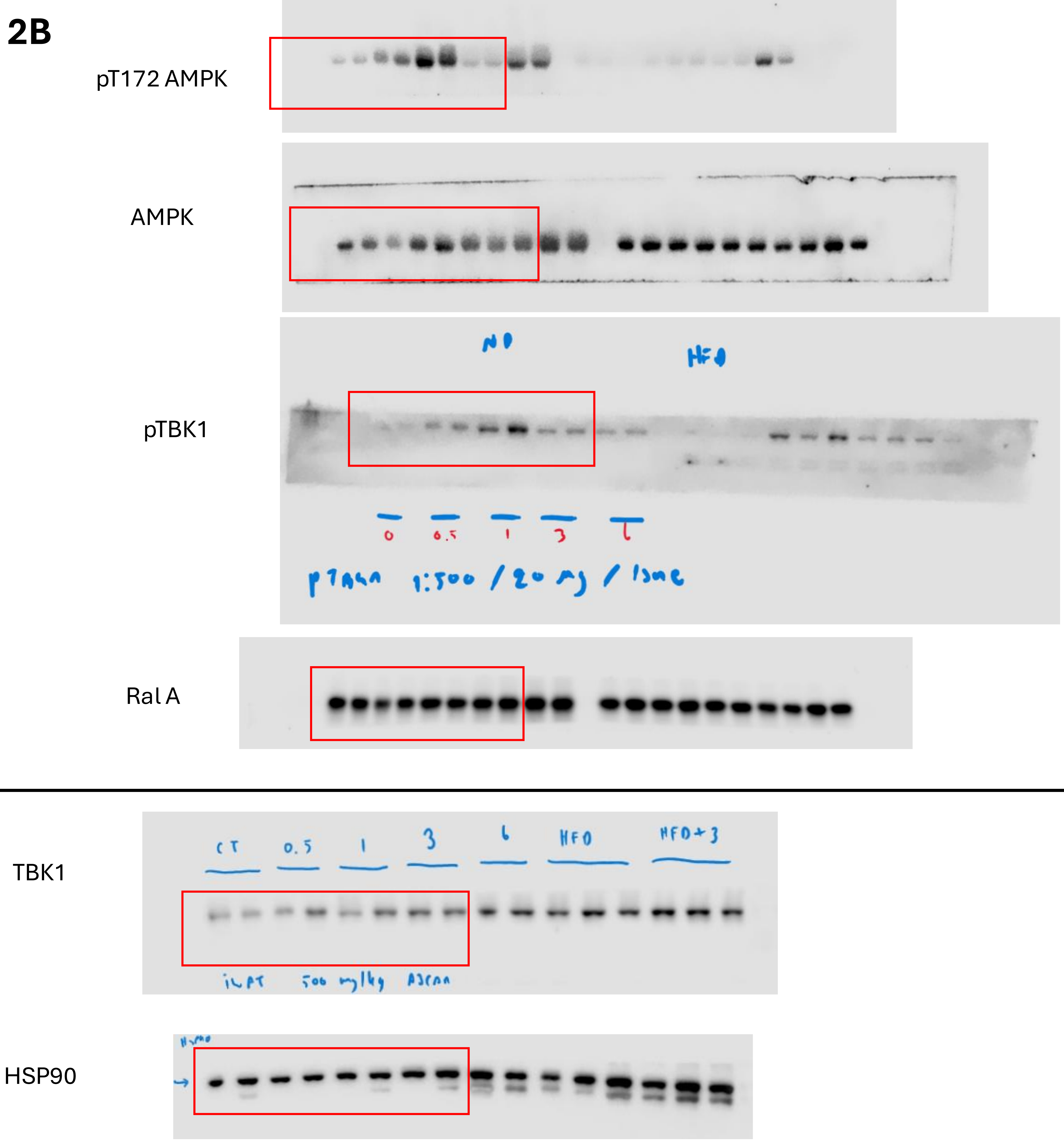
pT172 AMPK
AMPK
pTBK1
ND
HFD
0
0.5
1
3
6
Ral A
TBK1
CT
0.5
1
3
6
HFD
HFD+3
HSP90

Figure 3G

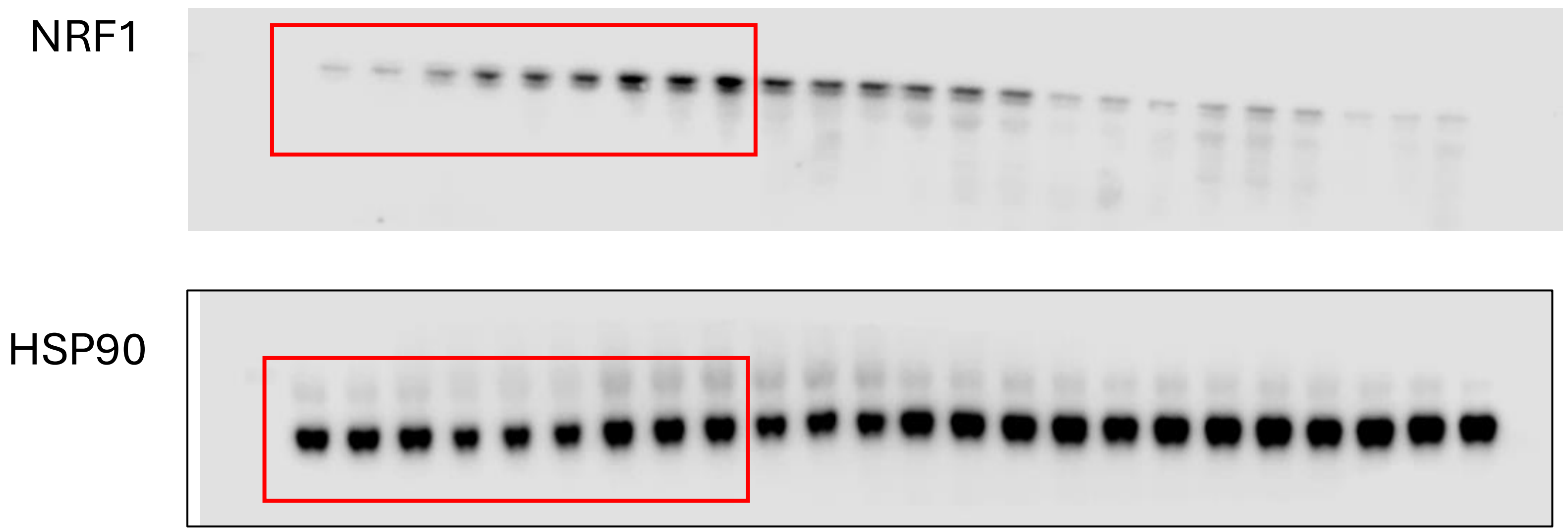

Figure 5F

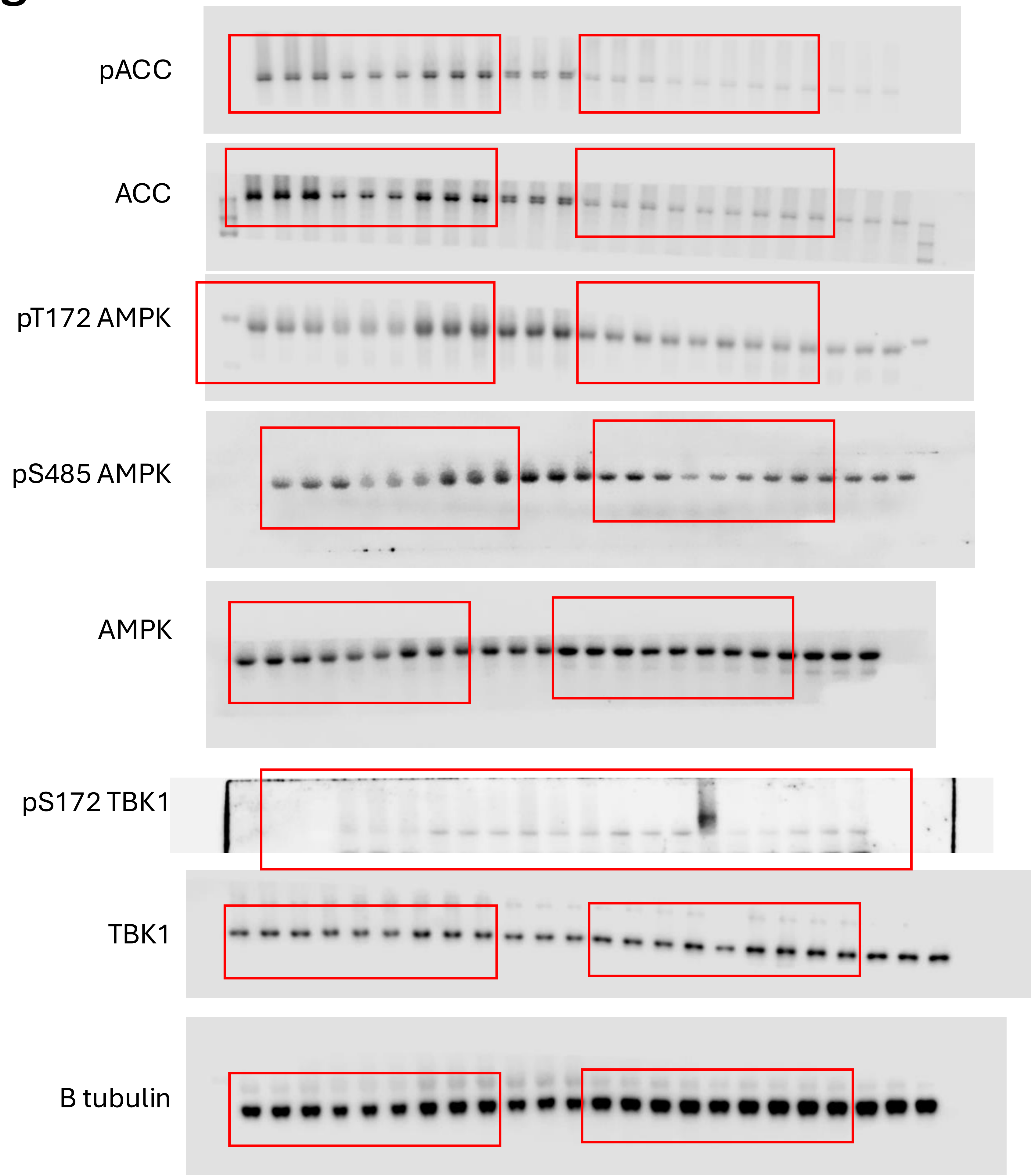
pACC
ACC
pT172 AMPK
pS485 AMPK
AMPK
pS172 TBK1
TBK1
B tubulin

Figure 6C

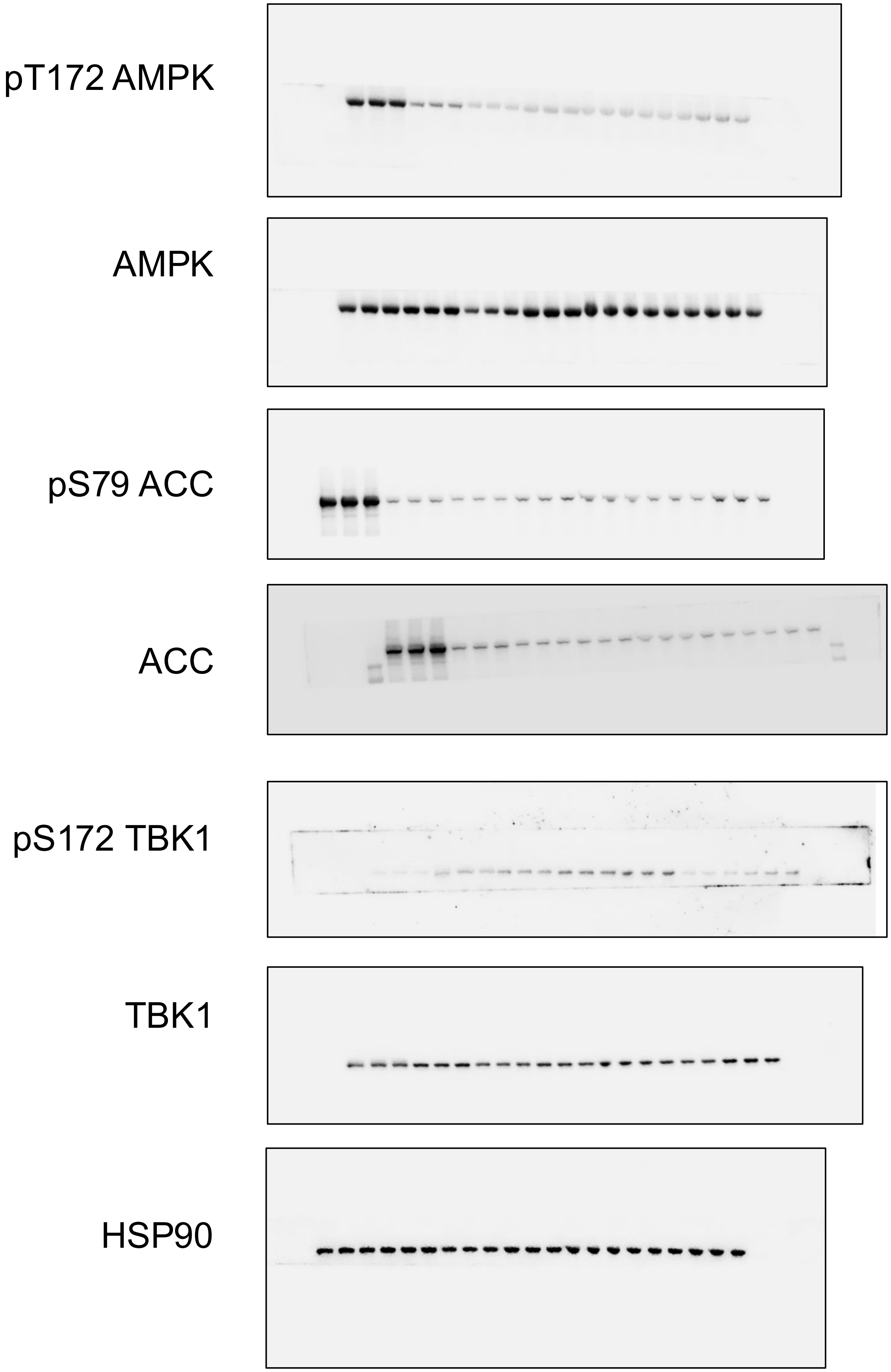

Figure 7E

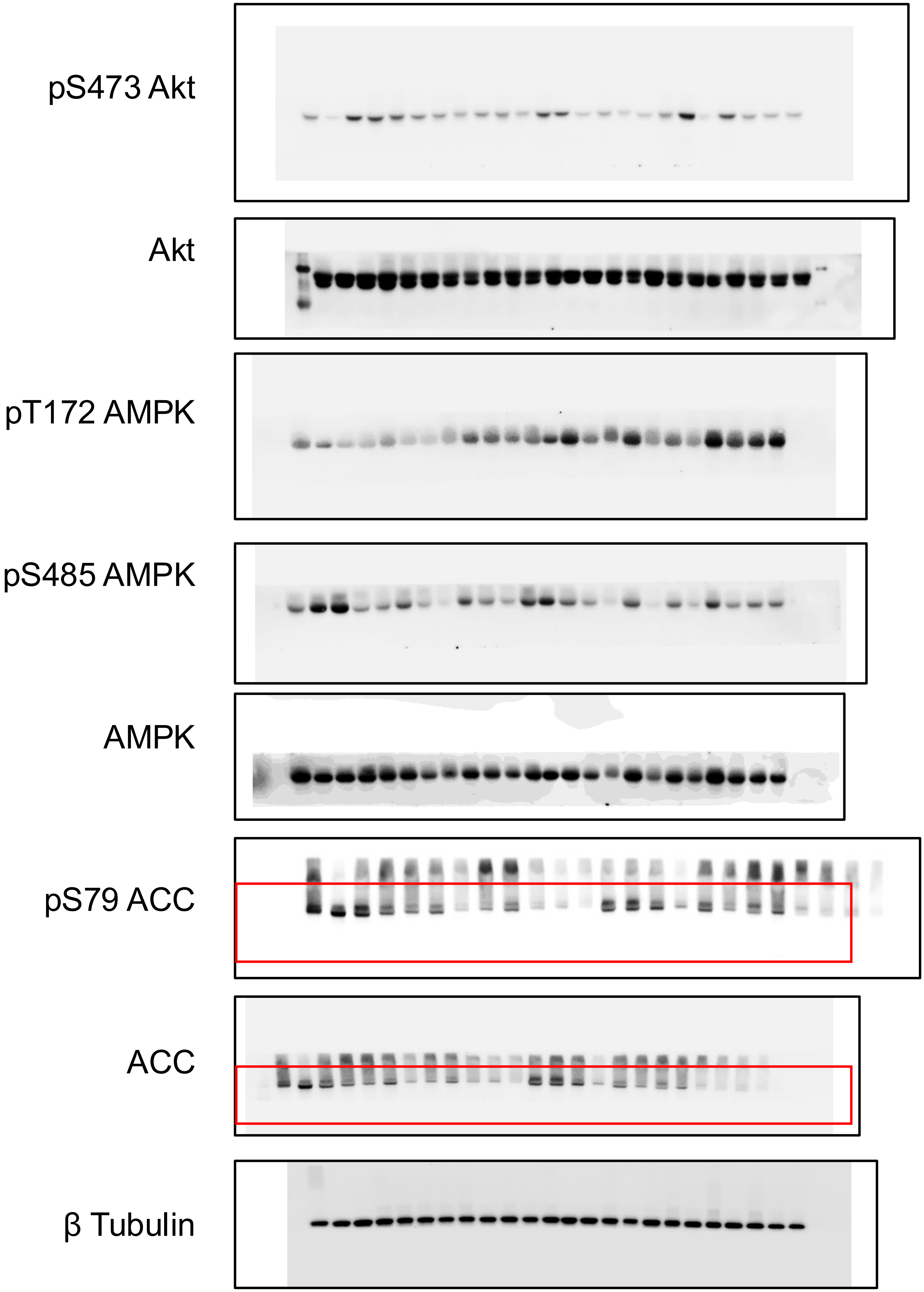
pS473 Akt
Akt
pT172 AMPK
pS485 AMPK
AMPK
pS79 ACC
ACC
β Tubulin

Figure 8H

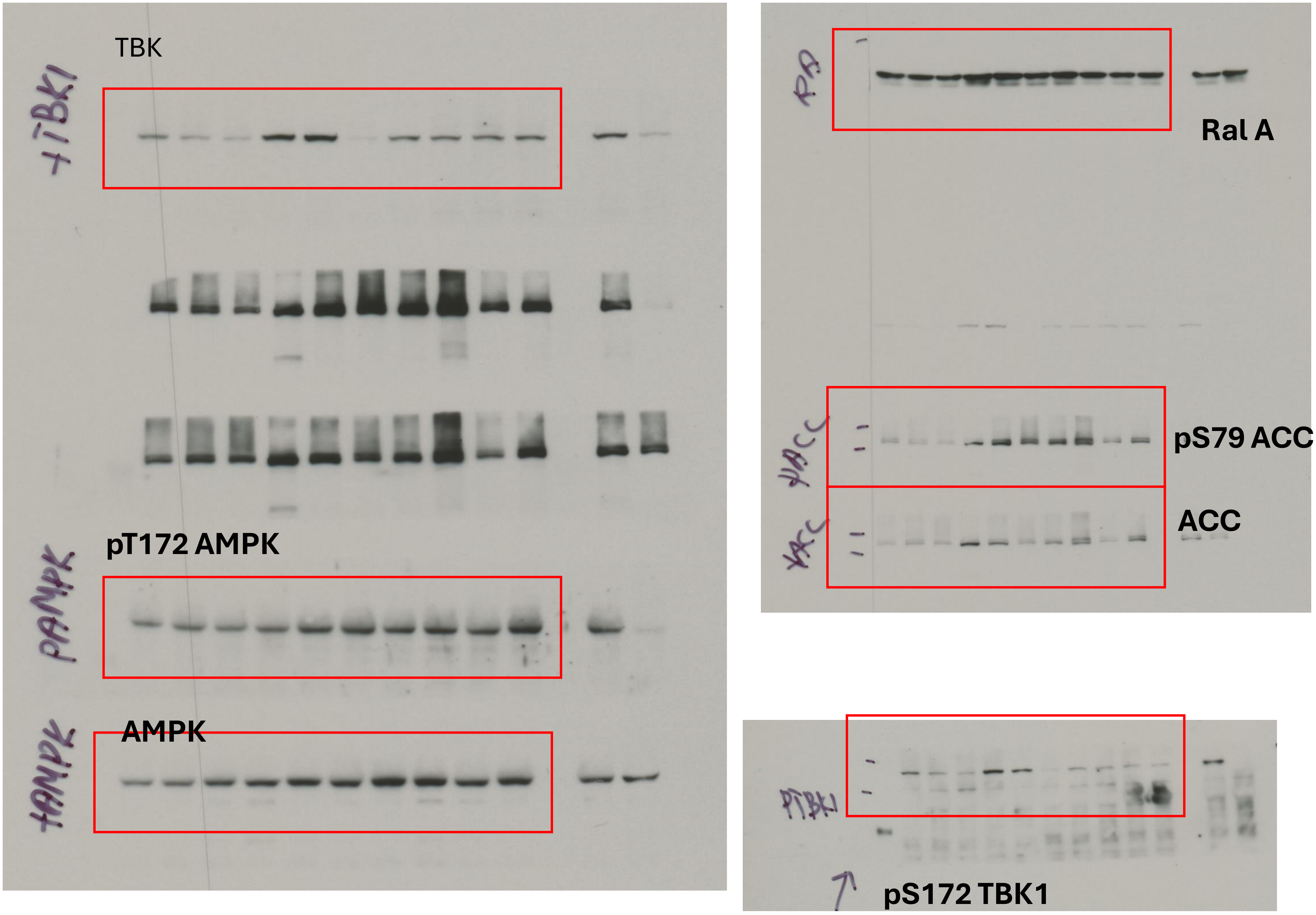

Figure 8J

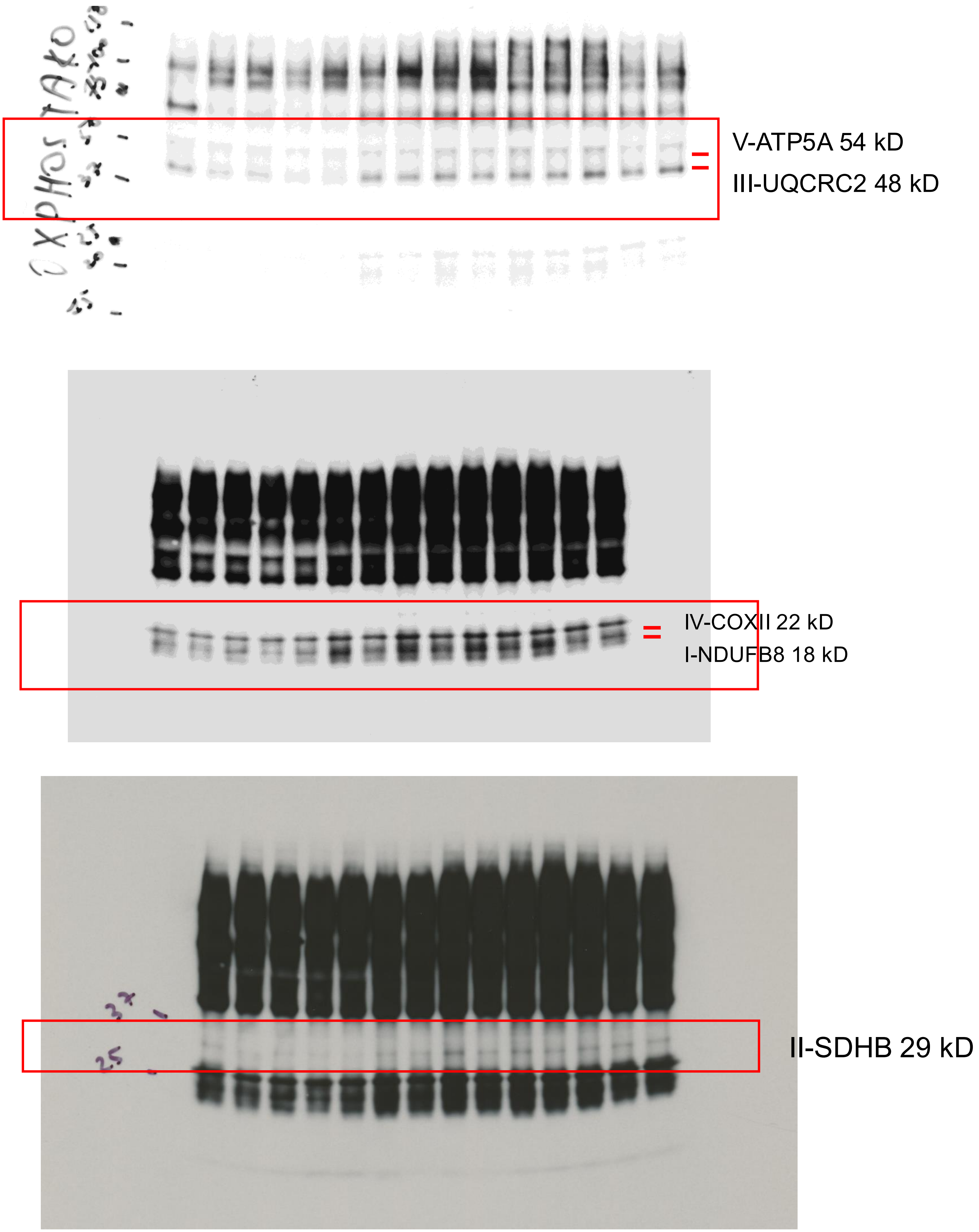
V-ATP5A 54 kD
III-UQCRC2 48 kD
IV-COXII 22 kD
I-NDUFB8 18 kD
II-SDHB 29 kD

Figure 11D

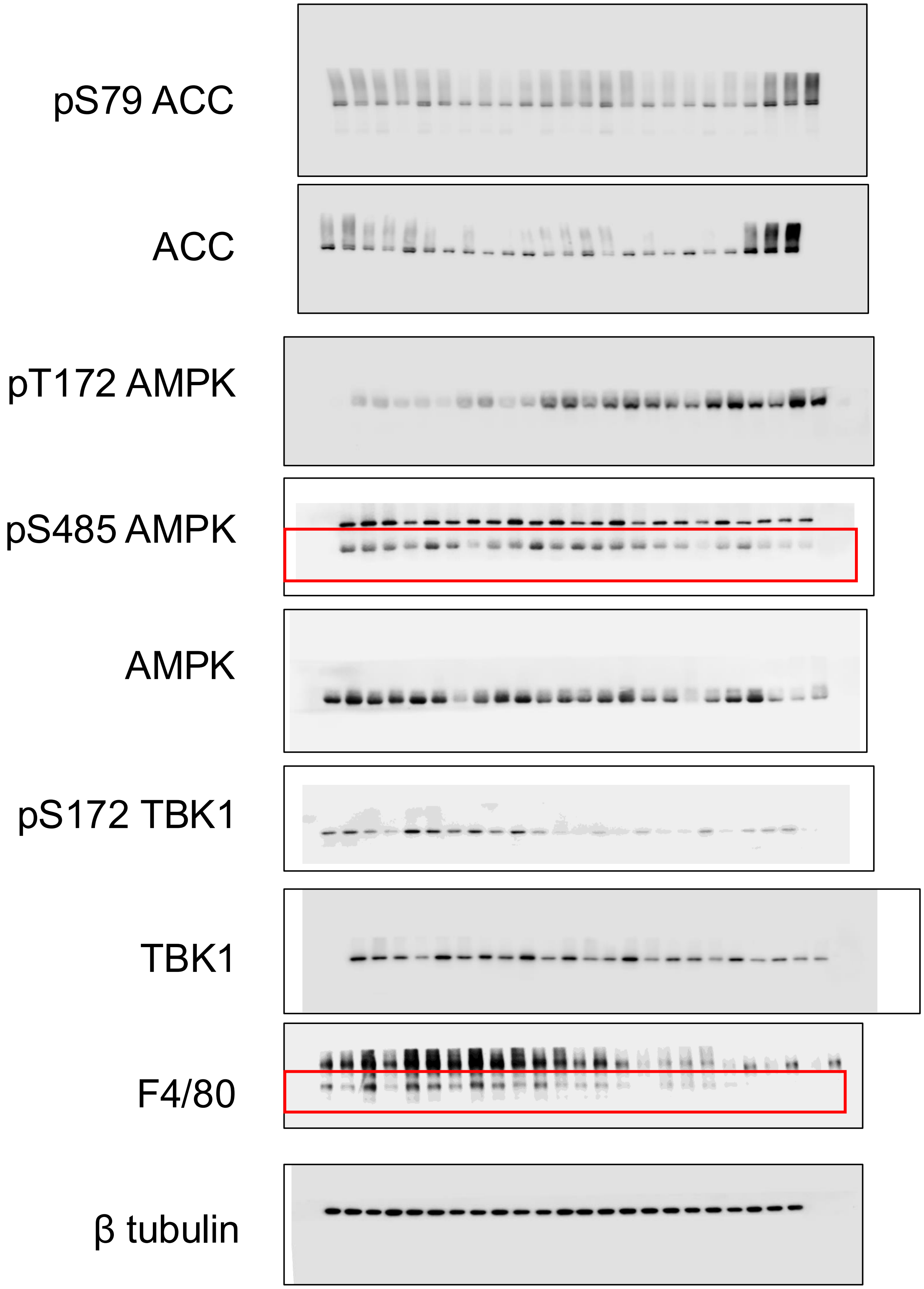
pS79 ACC
ACC
pT172 AMPK
pS485 AMPK
AMPK
pS172 TBK1
TBK1
F4/80
β tubulin

Figure 12 A

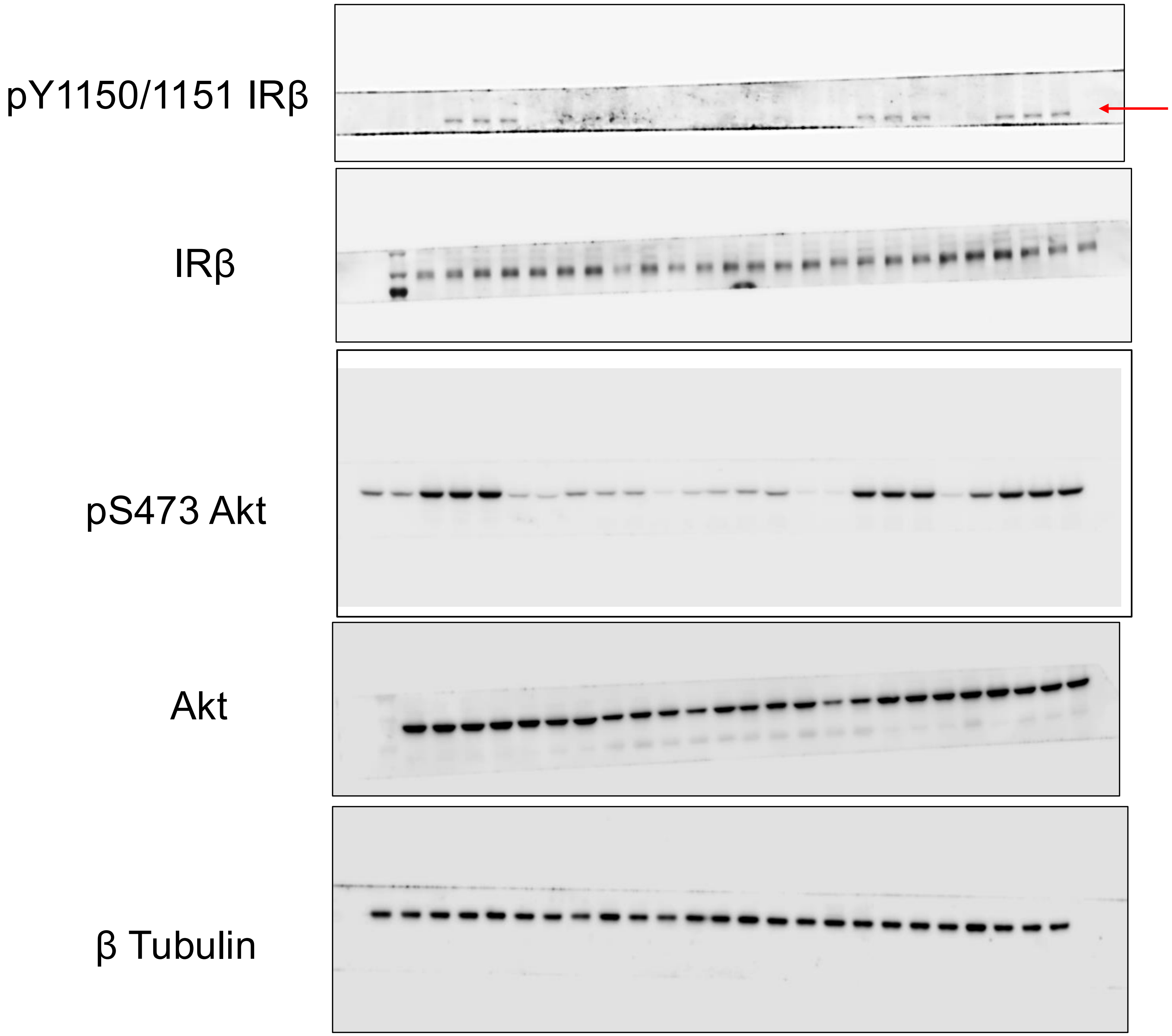

Figure 12C

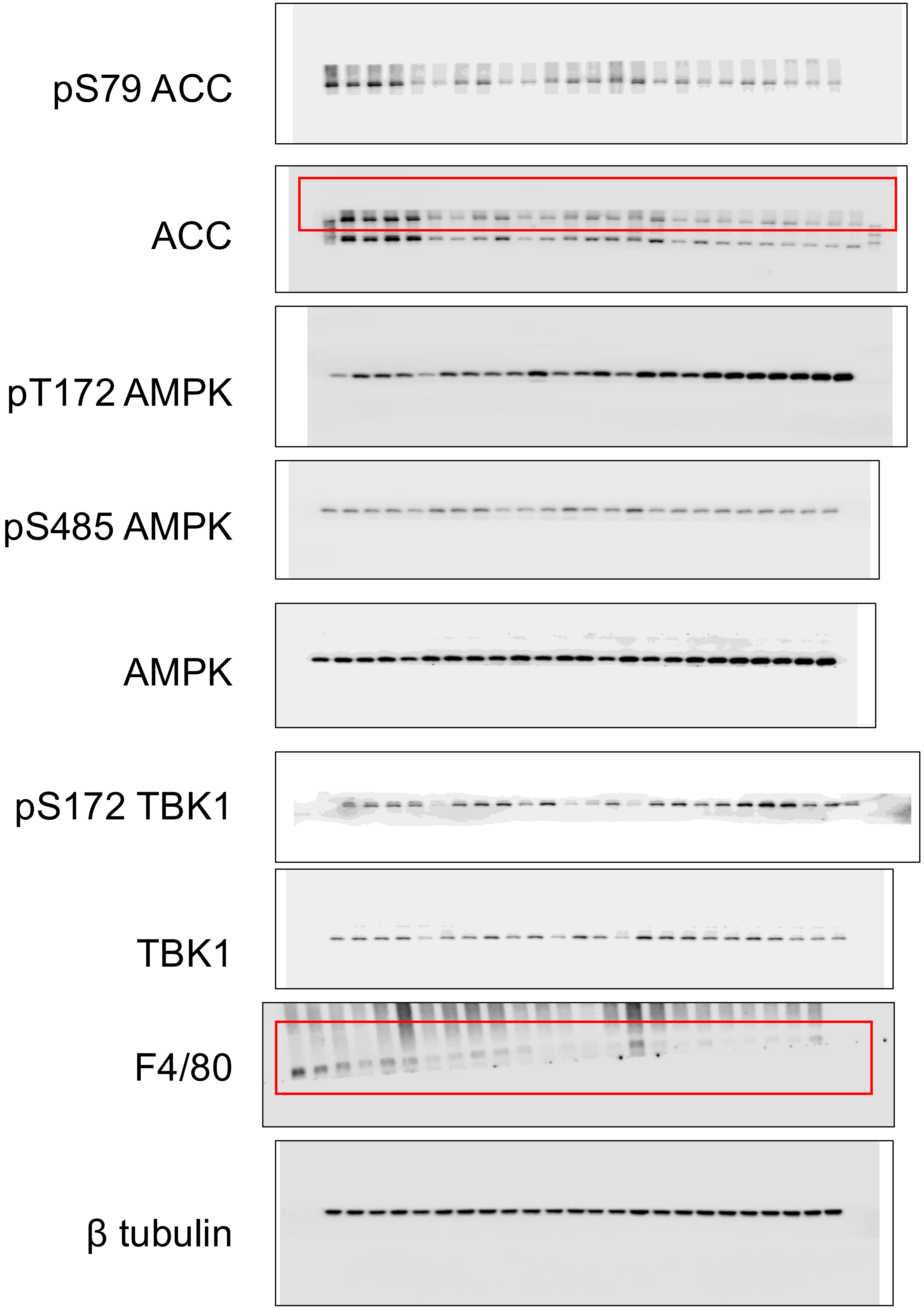
pS79 ACC
ACC
pT172 AMPK
pS485 AMPK
AMPK
pS172 TBK1
TBK1
F4/80
β tubulin

SUPPLEMENT FIG 1C

pS79 ACC

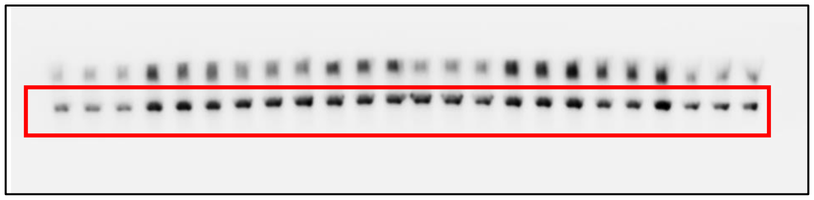

ACC

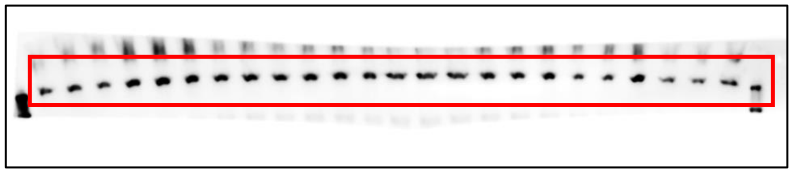

pS555 ULK1

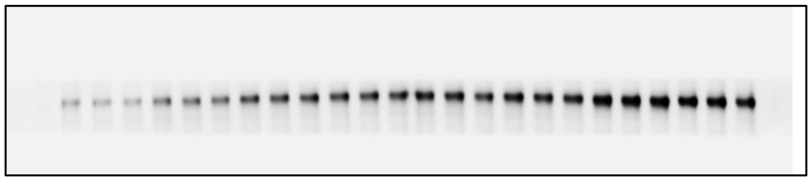

ULK1

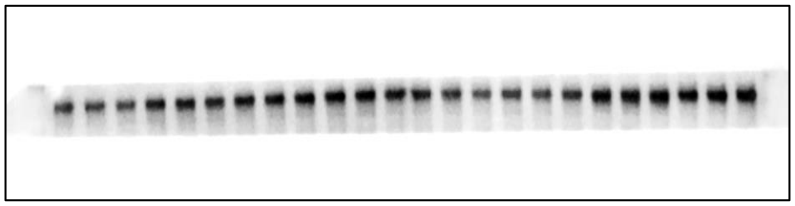

pS792 Raptor

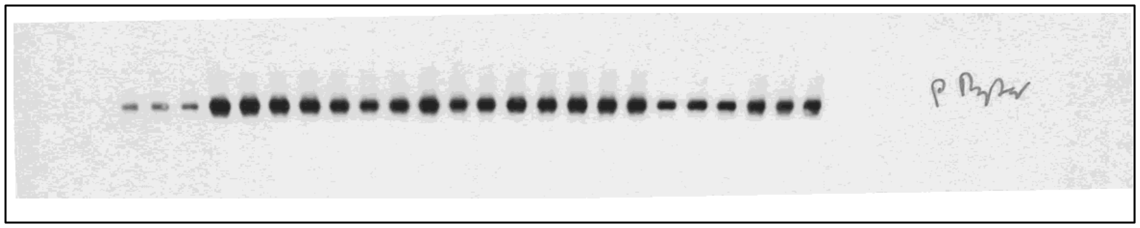

Raptor

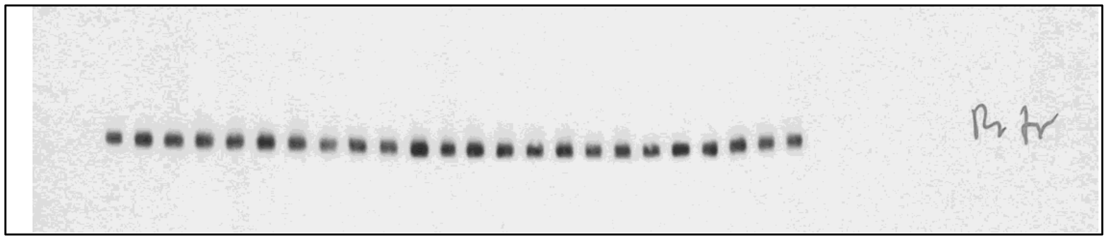

pT172 AMPK

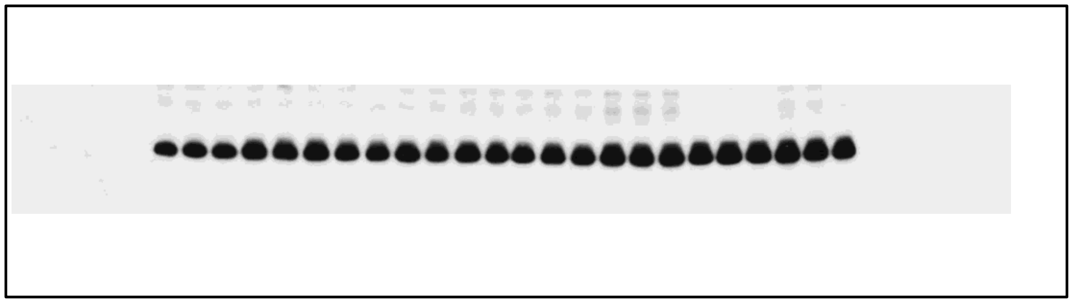

AMPK

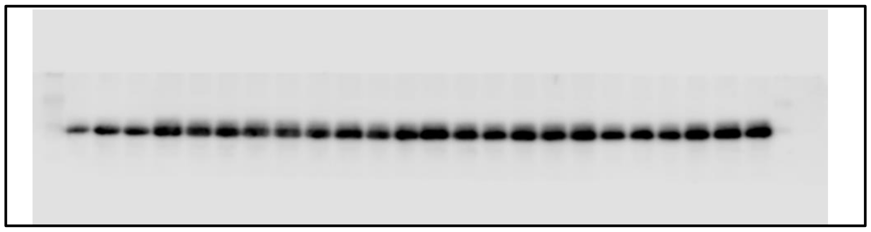

pS172 TBK1

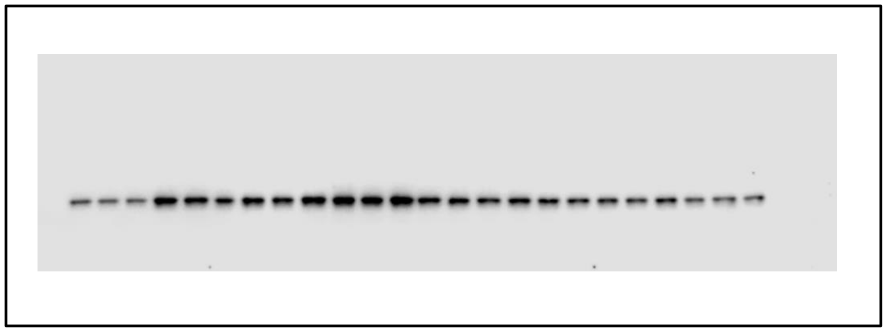

TBK1

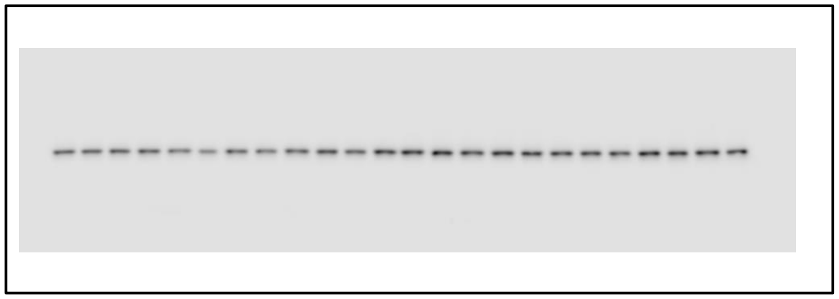

RalA

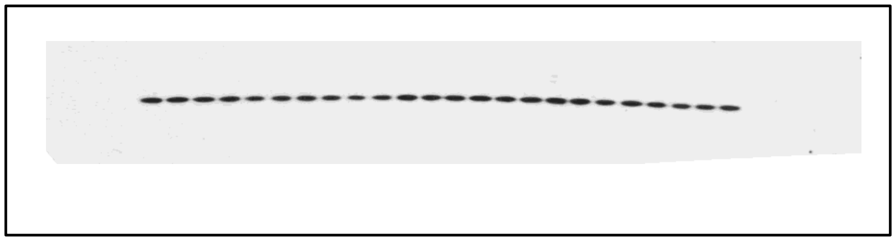

SUPPLEMENT FIG 1E

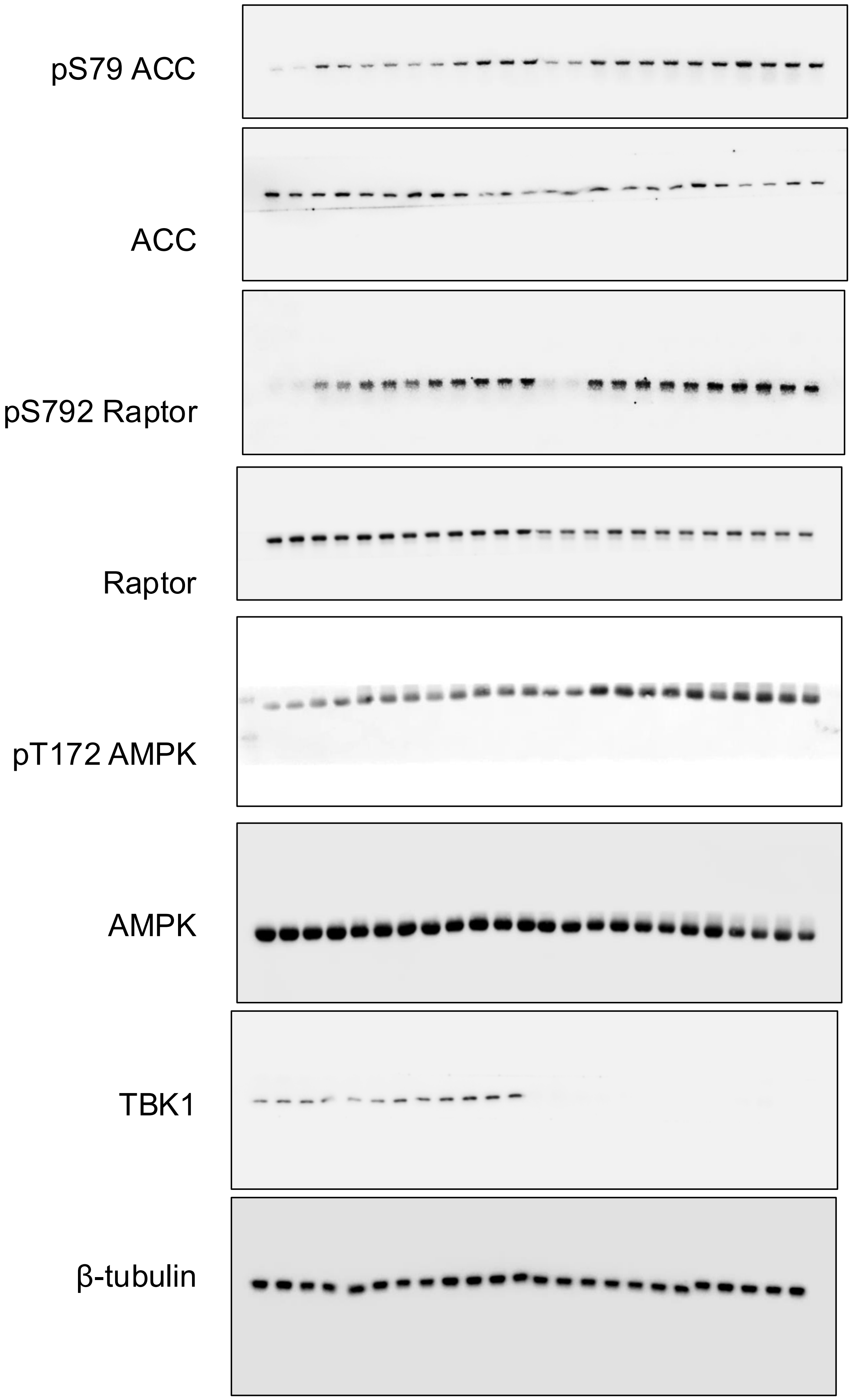

supplemental fig 3F

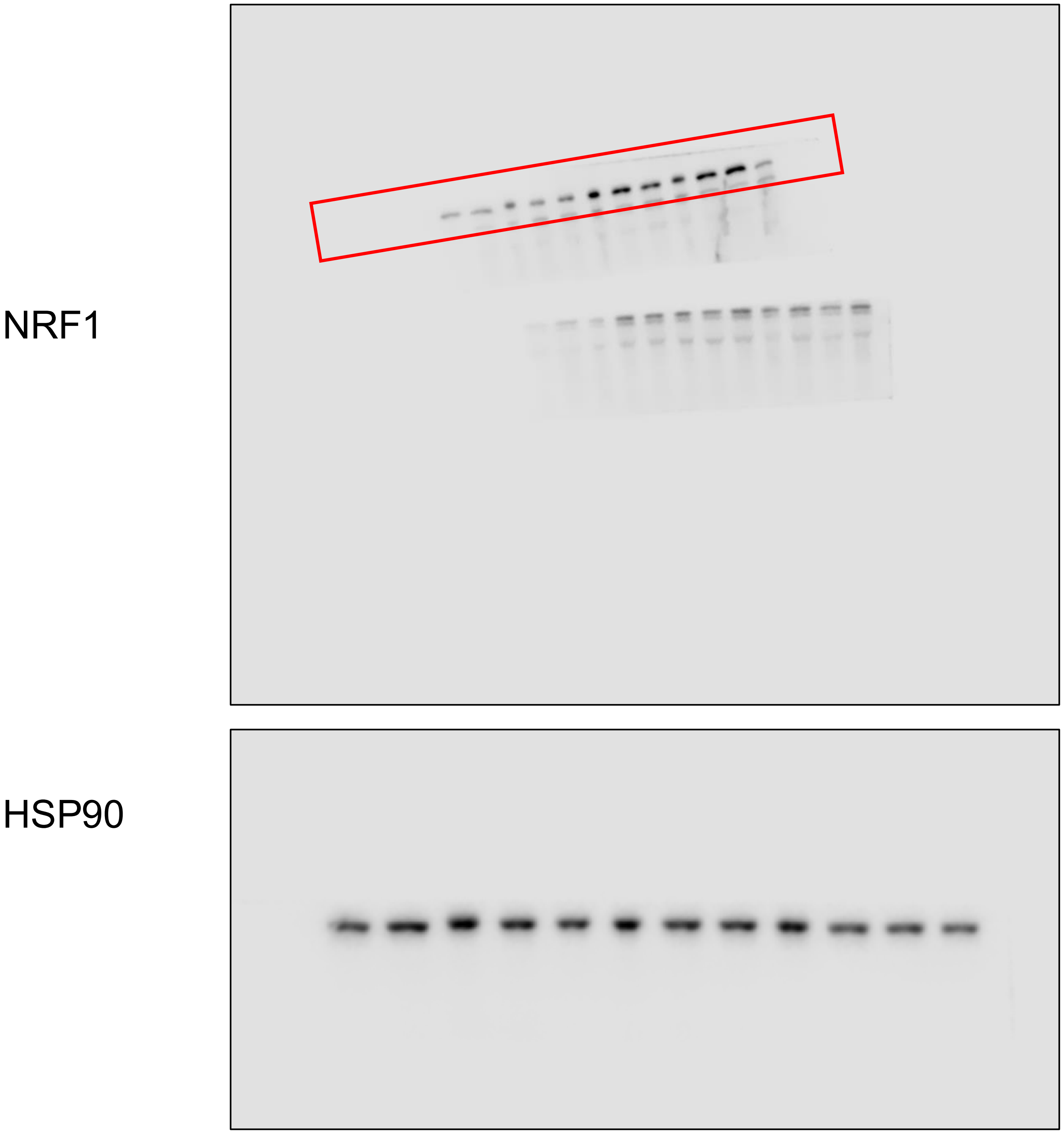

SUPPLEMENT FIG 2A

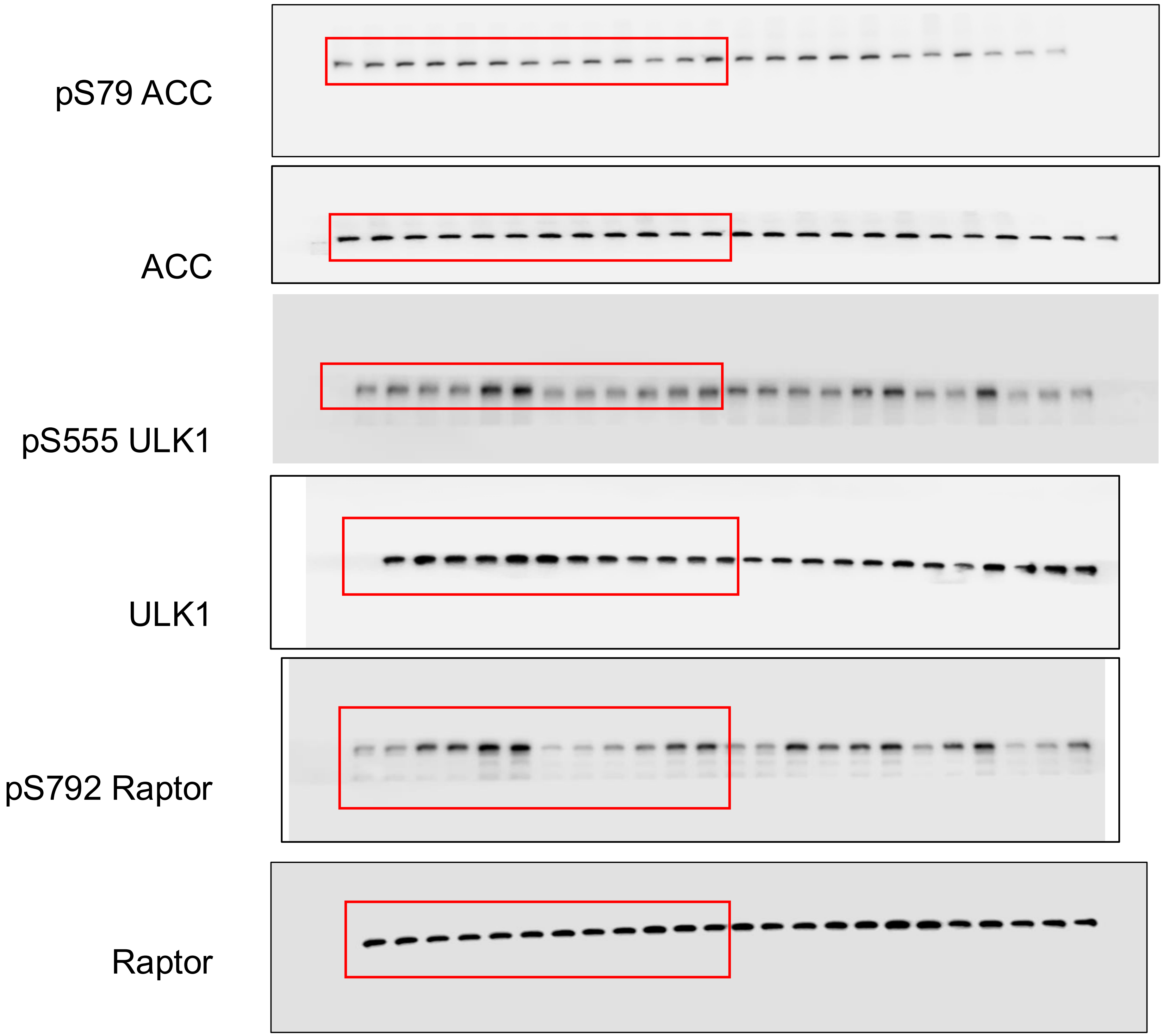

SUPPLEMENT FIG 2A-2

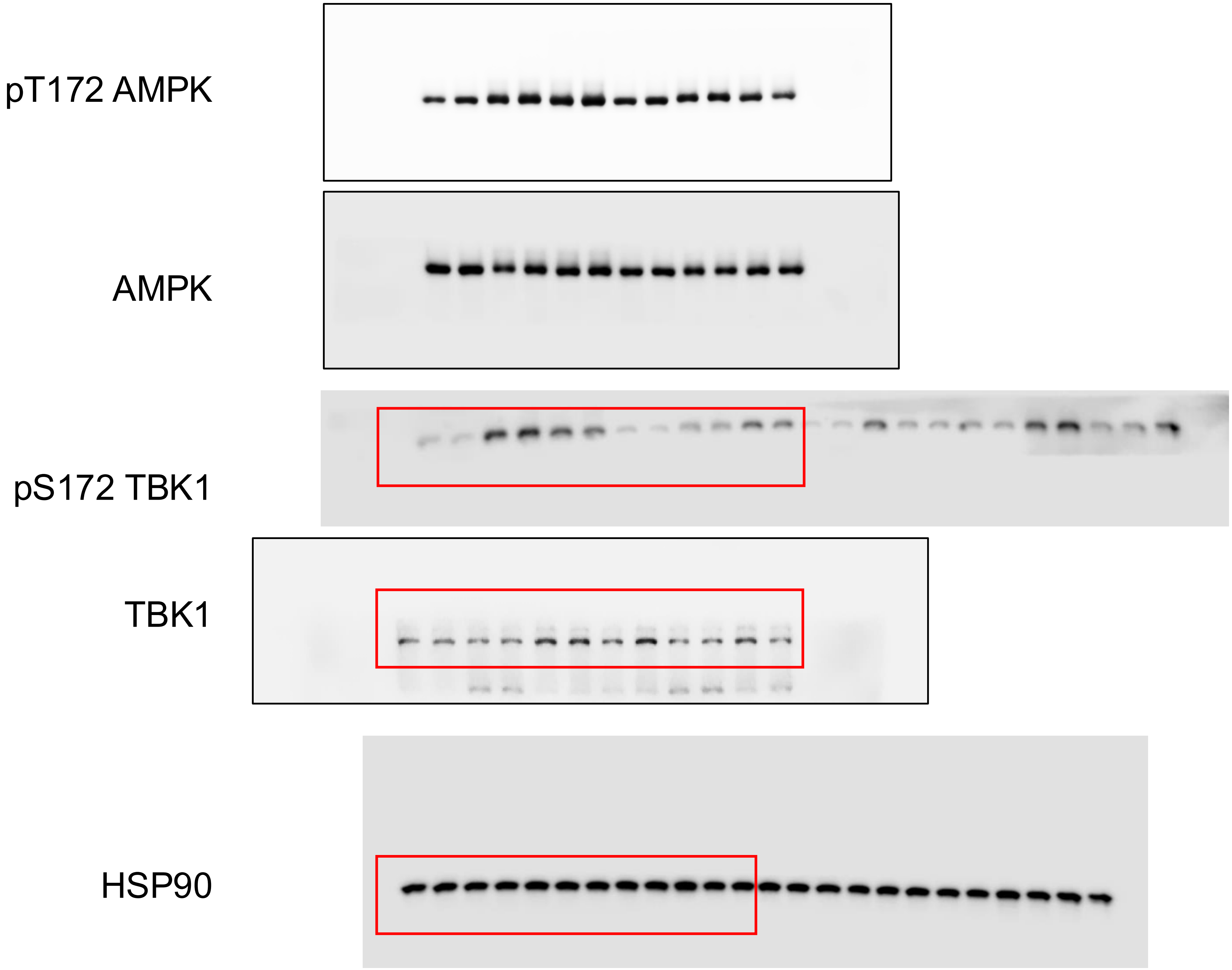

Supplemental Fig 2D

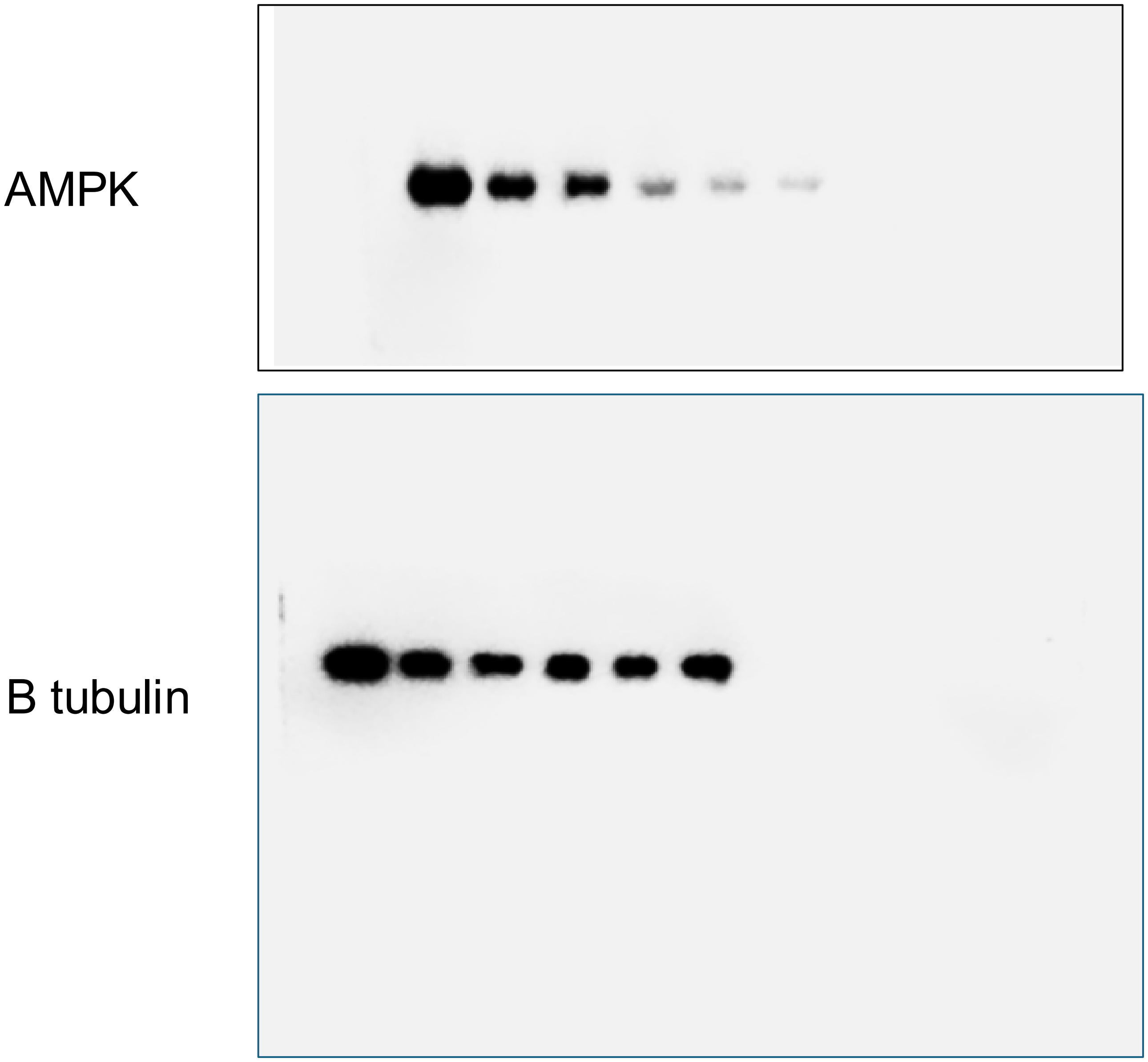

Sup Fig 3H

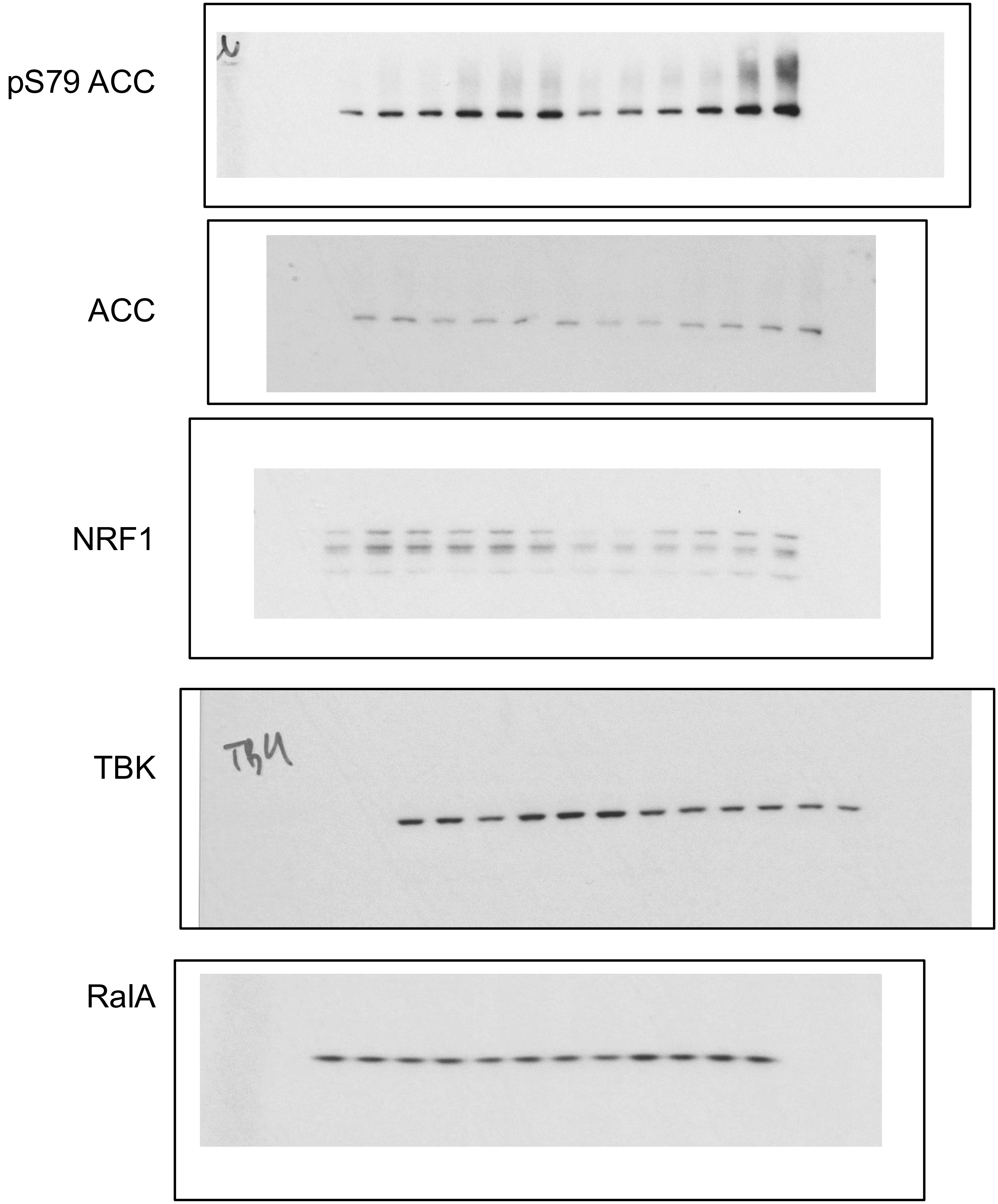

Sup Fig 3J

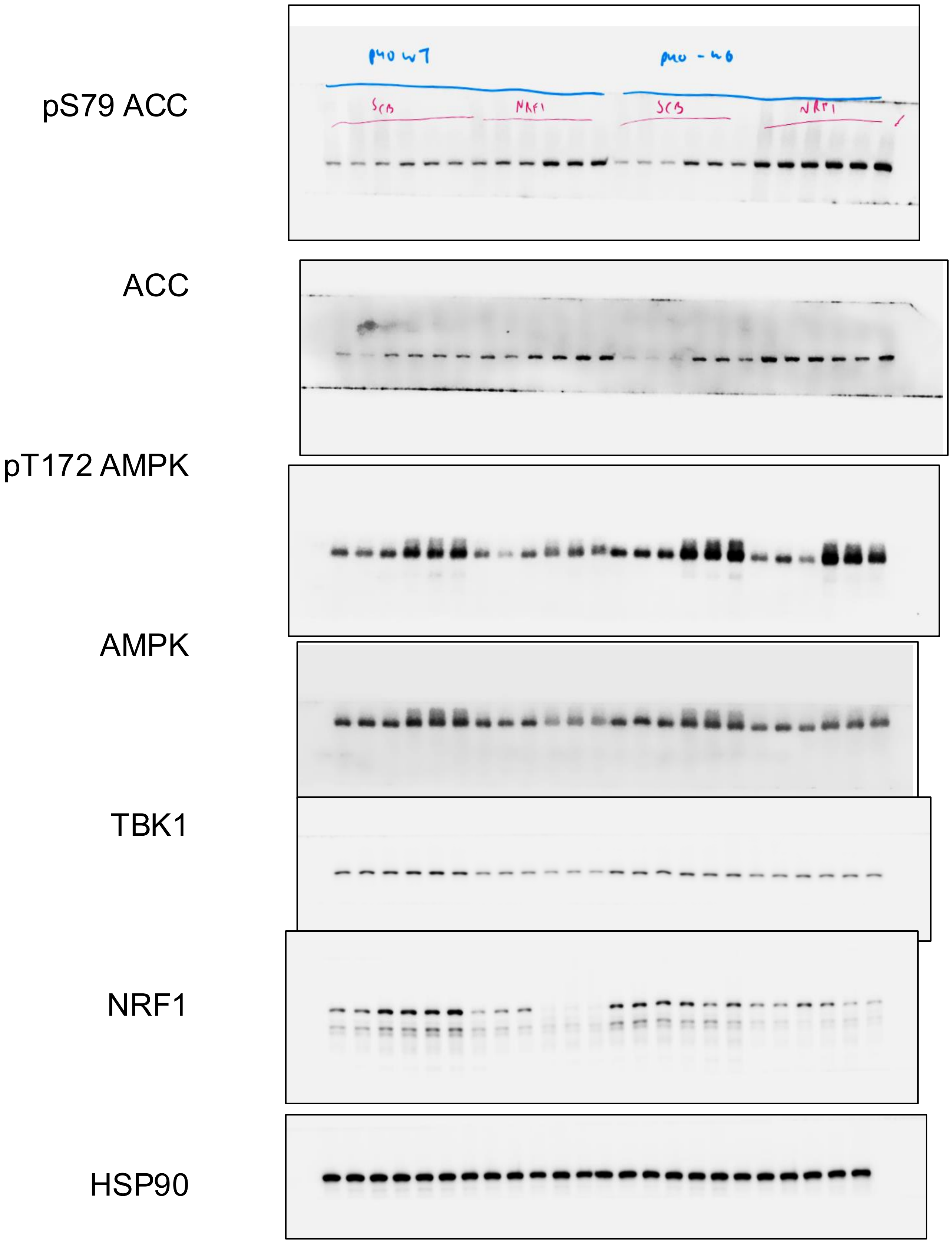

Supplemental Fig 4A

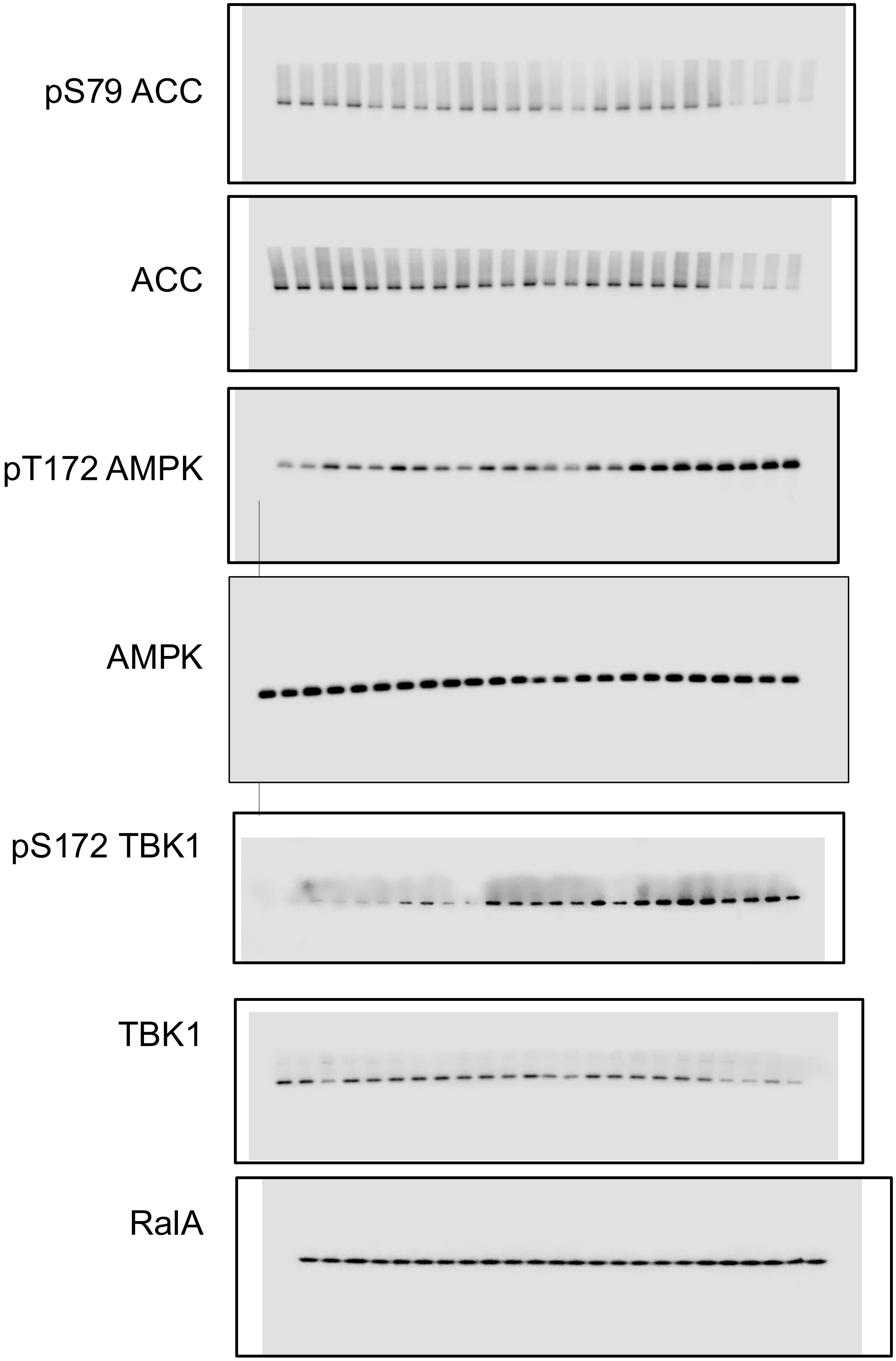

Supplementary fig 5D

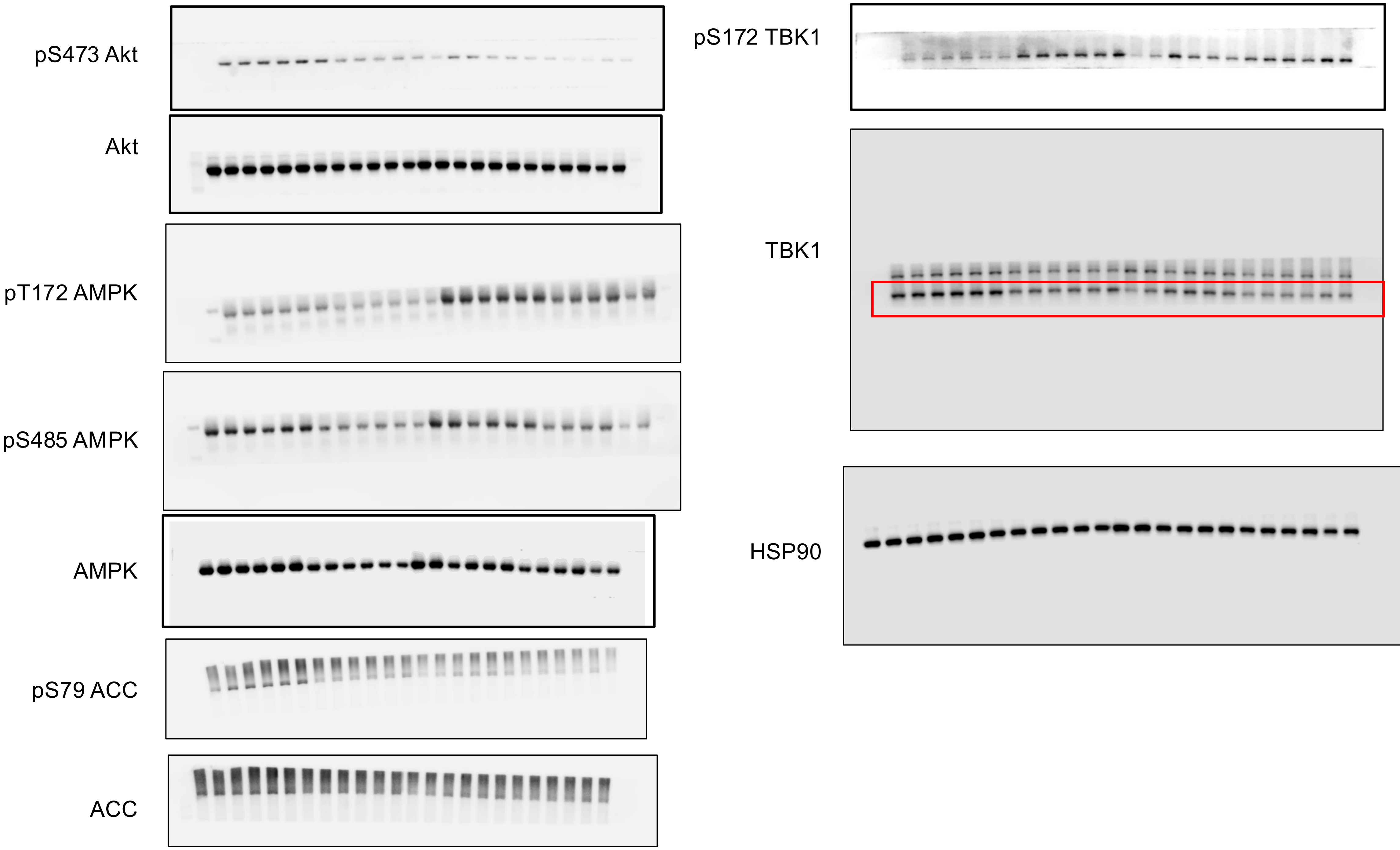

Supplementary fig 6G

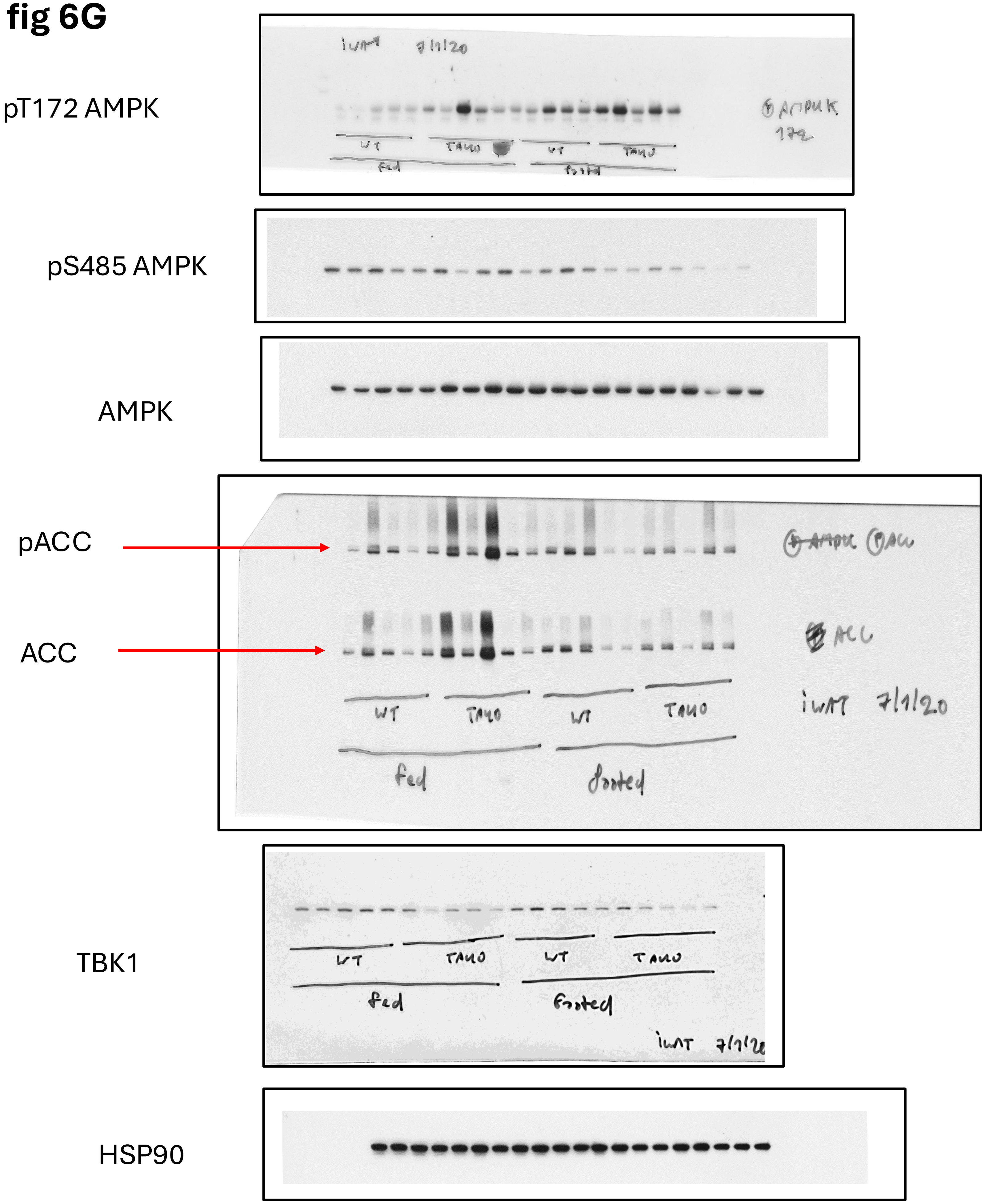

Supplementary fig 7B

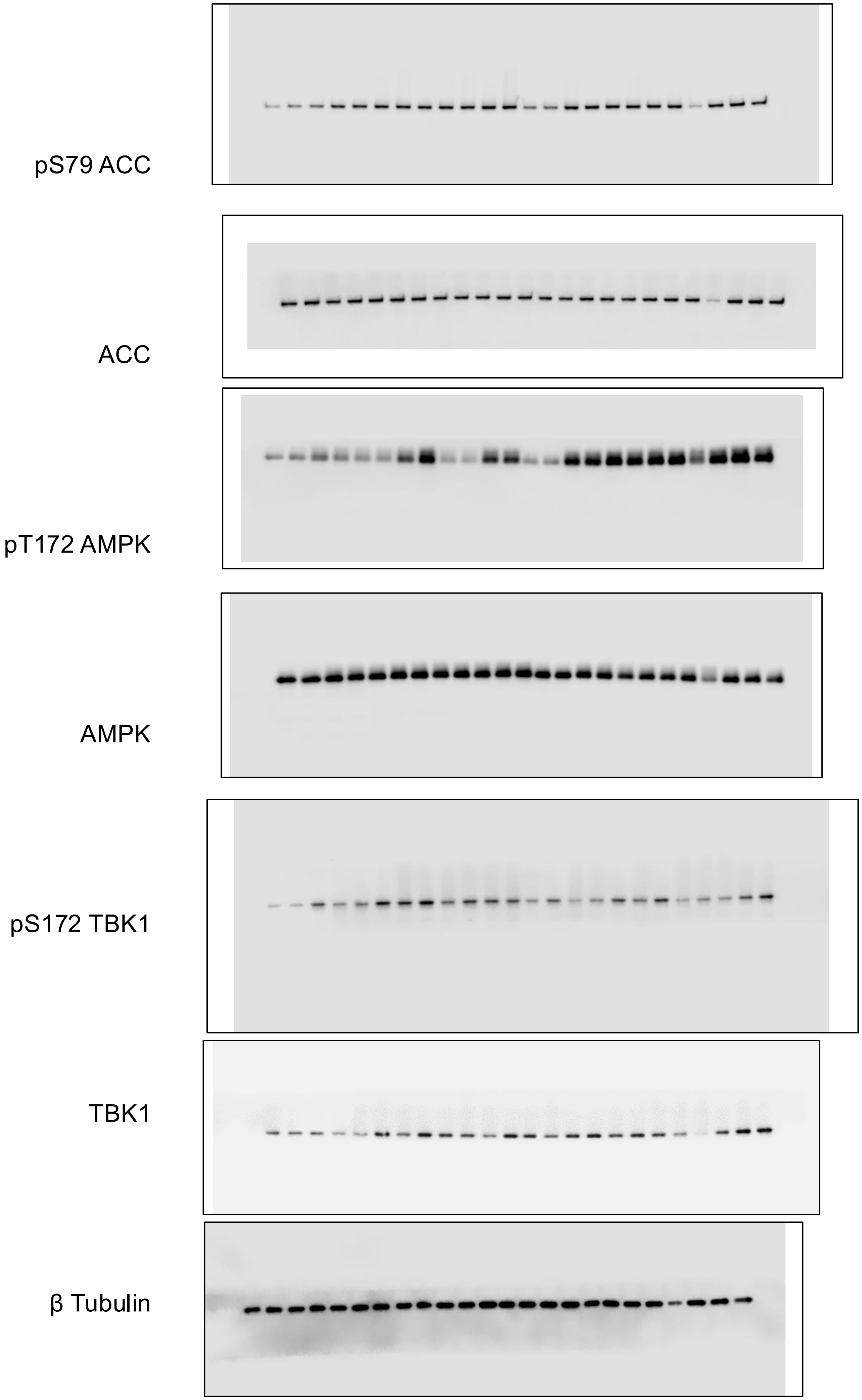

Figure 5F

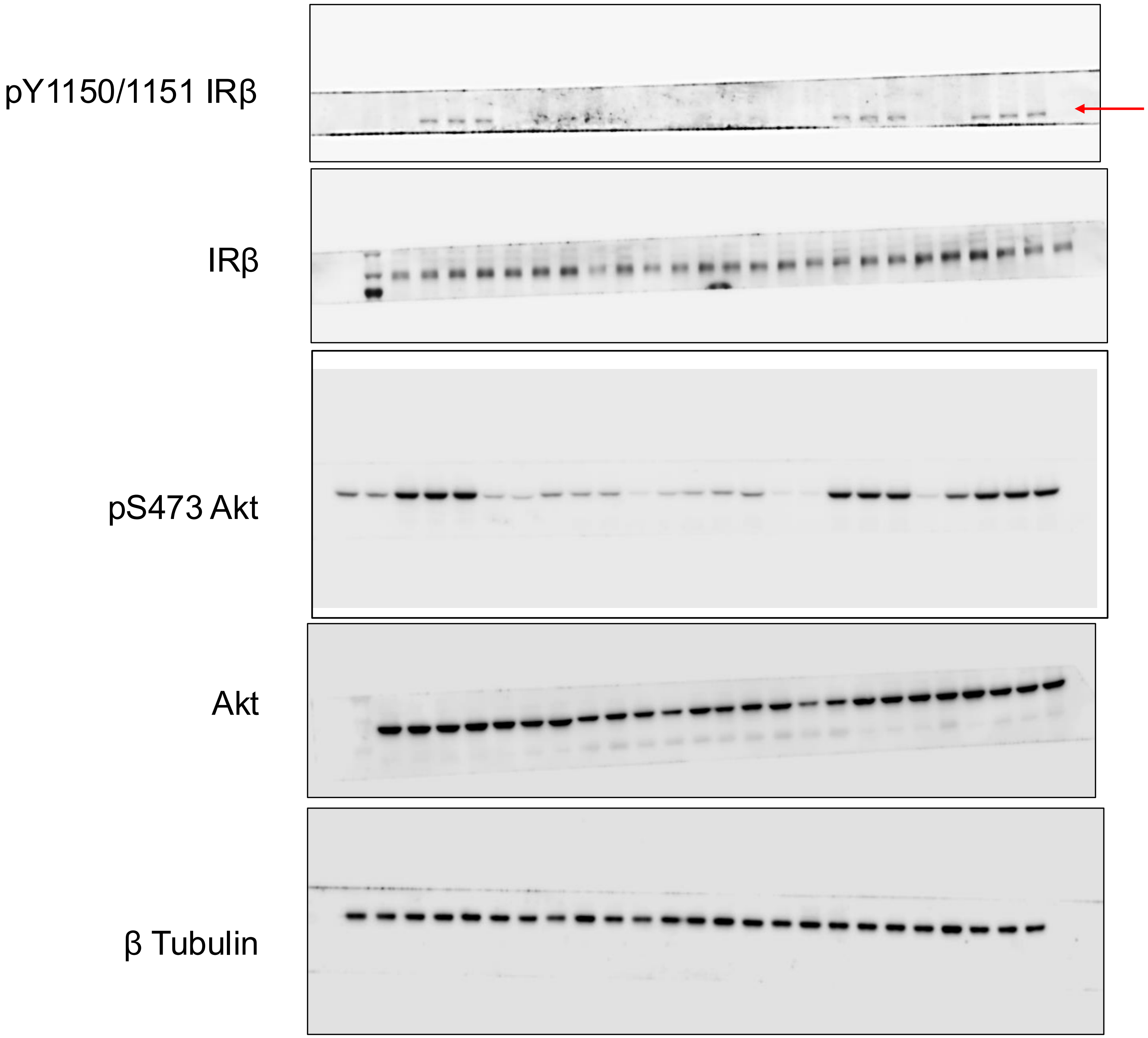

Supplementary figure 9B

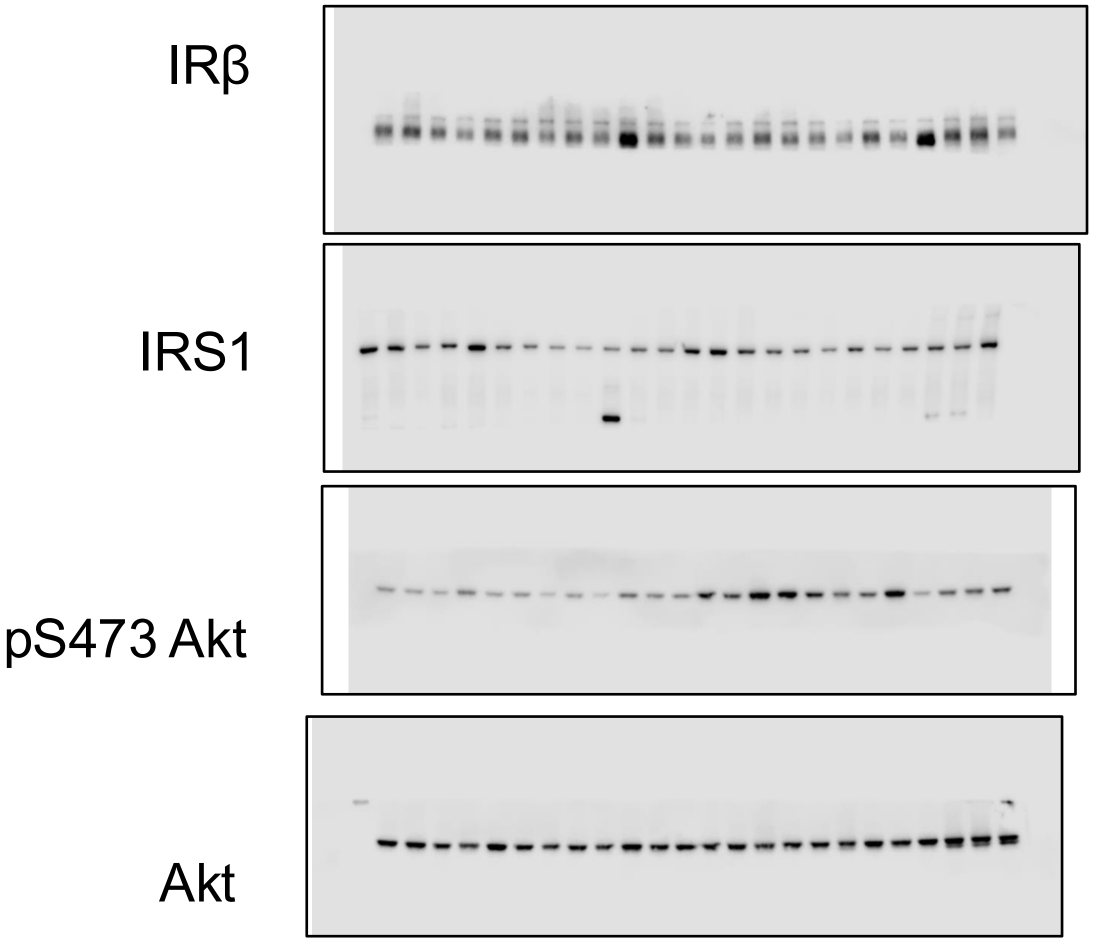

Supplementary figure 9D

pS473 Akt

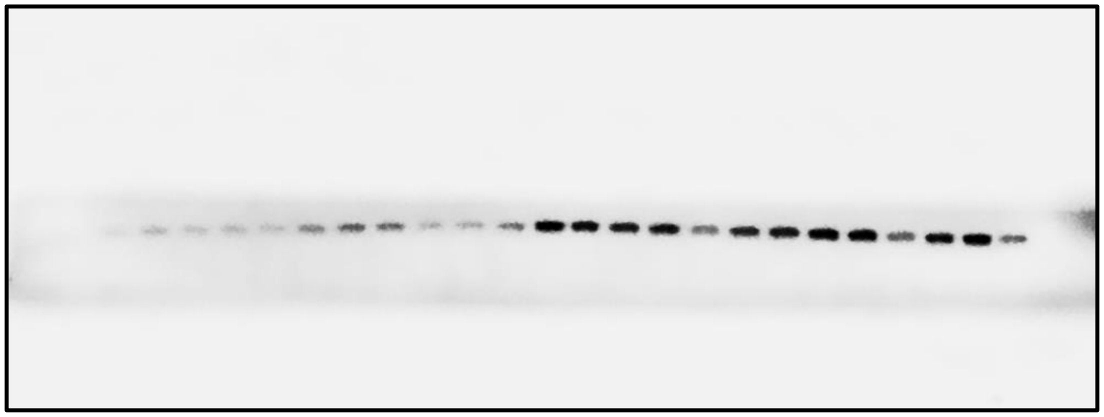

Akt

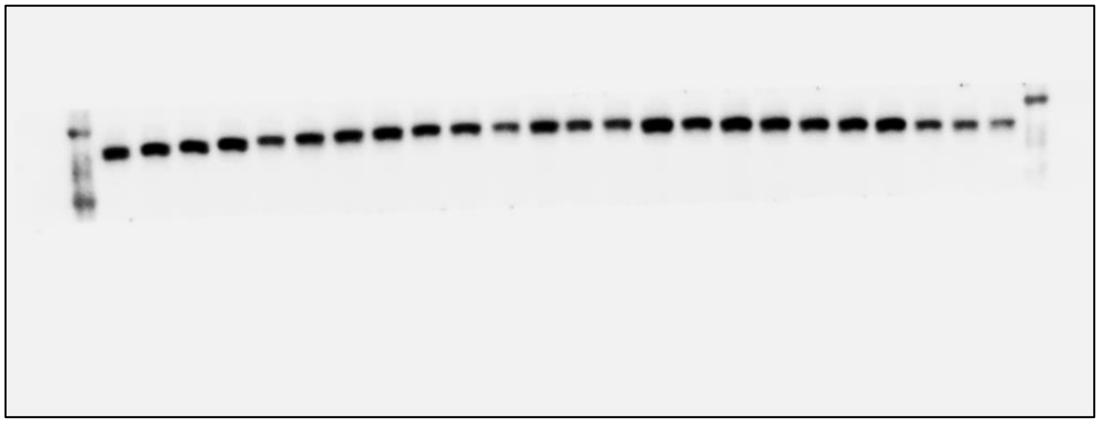

Supplement: Unedited blot and gel images [file jciinsight-11-200168-s259.pdf]
